# Personalized diagnosis in suspected myocardial infarction

**DOI:** 10.1007/s00392-023-02206-3

**Published:** 2023-05-02

**Authors:** Johannes Tobias Neumann, Raphael Twerenbold, Francisco Ojeda, Sally J. Aldous, Brandon R. Allen, Fred S. Apple, Hugo Babel, Robert H. Christenson, Louise Cullen, Eleonora Di Carluccio, Dimitrios Doudesis, Ulf Ekelund, Evangelos Giannitsis, Jaimi Greenslade, Kenji Inoue, Tomas Jernberg, Peter Kavsak, Till Keller, Kuan Ken Lee, Bertil Lindahl, Thiess Lorenz, Simon A. Mahler, Nicholas L. Mills, Arash Mokhtari, William Parsonage, John W. Pickering, Christopher J. Pemberton, Christoph Reich, A. Mark Richards, Yader Sandoval, Martin P. Than, Betül Toprak, Richard W. Troughton, Andrew Worster, Tanja Zeller, Andreas Ziegler, Stefan Blankenberg, the ARTEMIS study group, Emily Brownlee, Kai M. Eggers, Gavin Fincher, Norbert Frey, Niranjan Gaikwad, Vinay Gangathimmaiah, Emma Hall, Paul M. Haller, Christian Hamilton-Craig, Rebecca Hancock, Andrew Hobbins-King, Gerben Keijzers, Maryam Khorramshahi Bayat, Georgios Koliopanos, Jonas Lehmacher, Lina Ljung, Troy Madsen, Ehsan Mahmoodi, Ellyse McCormick, Bryn Mumma, Richard Nowak, Vanessa Blazquez, Siegfried Perez, Vazhma Qaderi, Isuru Ranasinghe, Alina Schock, Nils A. Sörensen, Andrew Staib, Laura Stephensen, Michael Weaver, R. Gentry Wilkerson, Anna Zournazi

**Affiliations:** 1 Department of Cardiology, University Heart and Vascular Center, University Medical Center Hamburg-Eppendorf, Martinistraße 52, 20246 Hamburg, Germany; 2 German Center for Cardiovascular Research (DZHK), Partner SiteHamburg/Kiel/Lübeck, Hamburg, Germany; 3 Population Health Research Department, University Heart and Vascular Center Hamburg, University Medical Center Hamburg-Eppendorf, Hamburg, Germany; 4 Department of Epidemiology and Preventive Medicine, School of Public Health and Preventive Medicine, Monash University, Melbourne, Australia; 5 University Center of Cardiovascular Science, University Heart and Vascular Center Hamburg, University Medical Center Hamburg-Eppendorf, Hamburg, Germany; 6 Department of Cardiology, Christchurch Hospital, Christchurch, New Zealand; 7 Department of Emergency Medicine, College of Medicine, University of Florida, Gainesville, FL USA; 8 Departments of Laboratory Medicine and Pathology, Hennepin Healthcare/HCMC and University of Minnesota, Minneapolis, MN USA; 9 Cardio-CARE, Medizincampus Davos, Davos, Switzerland; 10 Department of Pathology, University of Maryland School of Medicine, Baltimore, MD USA; 11 Department of Emergency Medicine, Royal Brisbane and Women’s Hospital, Herston, QLD Australia; 12 BHF Centre for Cardiovascular Science, University of Edinburgh, Edinburgh, UK; 13 Department of Internal and Emergency Medicine, Lund University, Skåne University Hospital, Lund, Sweden; 14 Department of Cardiology, Heidelberg University Hospital, Heidelberg, Germany; 15 Juntendo University Nerima Hospital, Tokyo, Japan; 16 Department of Clinical Sciences, Danderyd University Hospital, Karolinska Institutet, Stockholm, Sweden; 17 Department of Pathology and Molecular Medicine, McMaster University, Hamilton, ON Canada; 18 Department of Cardiology, Kerckhoff Heart and Thorax Center, Bad Nauheim, Germany; 19 Department of Medical Sciences and Uppsala Clinical Research Center, Uppsala University, Uppsala, Sweden; 20 Department of Emergency Medicine, Wake Forest School of Medicine, Winston-Salem, NC USA; 21 Department of Internal Medicine and Emergency Medicine and Department of Cardiology, Lund University, Skåne University Hospital, Lund, Sweden; 22 Australian Centre for Health Service Innovation, Queensland University of Technology, Kelvin Grove, Australia; 23 Department of Medicine, Christchurch and Emergency Department, University of Otago, Christchurch Hospital, Christchurch, New Zealand; 24 Department of Medicine, Christchurch Heart Institute, University of Otago, Christchurch, New Zealand; 25 Minneapolis Heart Institute, Abbott Northwestern Hospital, and Minneapolis Heart Institute Foundation, Minneapolis, MN USA; 26 Division of Emergency Medicine, McMaster University, Hamilton, ON Canada; 27 School of Mathematics, Statistics and Computer Science, University of KwaZulu-Natal, Pietermaritzburg, South Africa

**Keywords:** Acute myocardial infarction, Biomarker, Troponin, Machine learning, Probability, Super learner, Validation

## Abstract

**Background:**

In suspected myocardial infarction (MI), guidelines recommend using high-sensitivity cardiac troponin (hs-cTn)-based approaches. These require fixed assay-specific thresholds and timepoints, without directly integrating clinical information. Using machine-learning techniques including hs-cTn and clinical routine variables, we aimed to build a digital tool to directly estimate the individual probability of MI, allowing for numerous hs-cTn assays.

**Methods:**

In 2,575 patients presenting to the emergency department with suspected MI, two ensembles of machine-learning models using single or serial concentrations of six different hs-cTn assays were derived to estimate the individual MI probability (ARTEMIS model). Discriminative performance of the models was assessed using area under the receiver operating characteristic curve (AUC) and logLoss. Model performance was validated in an external cohort with 1688 patients and tested for global generalizability in 13 international cohorts with 23,411 patients.

**Results:**

Eleven routinely available variables including age, sex, cardiovascular risk factors, electrocardiography, and hs-cTn were included in the ARTEMIS models. In the validation and generalization cohorts, excellent discriminative performance was confirmed, superior to hs-cTn only. For the serial hs-cTn measurement model, AUC ranged from 0.92 to 0.98. Good calibration was observed. Using a single hs-cTn measurement, the ARTEMIS model allowed direct rule-out of MI with very high and similar safety but up to tripled efficiency compared to the guideline-recommended strategy.

**Conclusion:**

We developed and validated diagnostic models to accurately estimate the individual probability of MI, which allow for variable hs-cTn use and flexible timing of resampling. Their digital application may provide rapid, safe and efficient personalized patient care.

**Trial Registration numbers:**

Data of following cohorts were used for this project: BACC (www.clinicaltrials.gov; NCT02355457), stenoCardia (www.clinicaltrials.gov; NCT03227159), ADAPT-BSN (www.australianclinicaltrials.gov.au; ACTRN12611001069943), IMPACT (www.australianclinicaltrials.gov.au, ACTRN12611000206921), ADAPT-RCT (www.anzctr.org.au; ANZCTR12610000766011), EDACS-RCT (www.anzctr.org.au; ANZCTR12613000745741); DROP-ACS (https://www.umin.ac.jp, UMIN000030668); High-STEACS (www.clinicaltrials.gov; NCT01852123), LUND (www.clinicaltrials.gov; NCT05484544), RAPID-CPU (www.clinicaltrials.gov; NCT03111862), ROMI (www.clinicaltrials.gov; NCT01994577), SAMIE (https://anzctr.org.au; ACTRN12621000053820), SEIGE and SAFETY (www.clinicaltrials.gov; NCT04772157), STOP-CP (www.clinicaltrials.gov; NCT02984436),

UTROPIA (www.clinicaltrials.gov; NCT02060760).

**Graphical Abstract:**

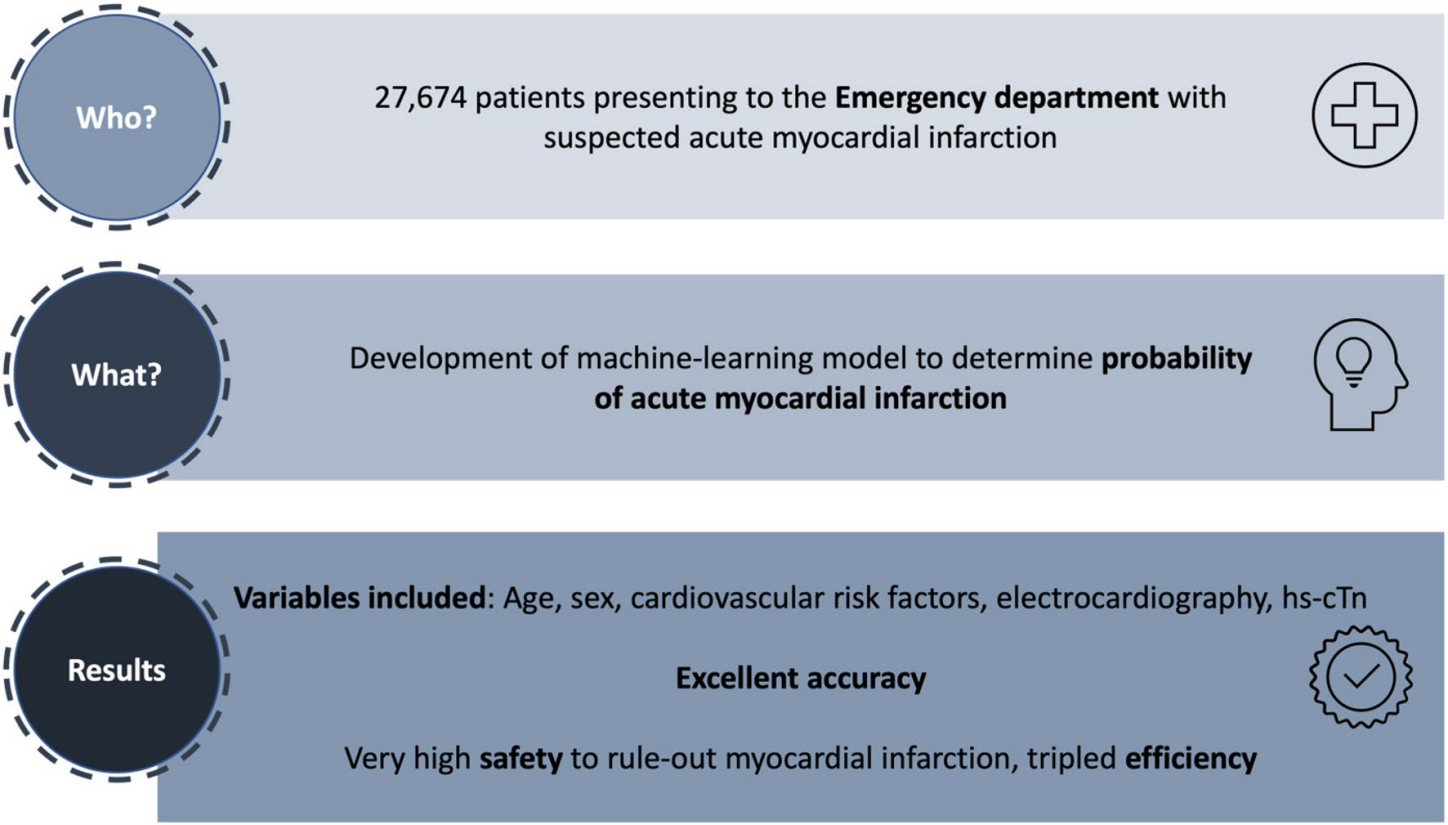

**Supplementary Information:**

The online version contains supplementary material available at 10.1007/s00392-023-02206-3.

## Introduction

Symptoms suggestive of myocardial infarction (MI) are a major reason for presentation to the emergency departments (ED) worldwide [1]. Measurement of cardiac troponin is crucial to diagnose or to rule out non-ST-elevation MI (NSTEMI) [2, 3]. For the management of patients with suspected NSTEMI, current guidelines recommend the application of high-sensitivity cardiac troponin (hs-cTn) assay-specific thresholds such as the 99th percentile or study-derived cut-offs for measurements obtained directly at presentation and, depending on the selected diagnostic approach, during serial sampling after one, two or three hours. [3–7]

Application of fixed assay-specific hc-cTn thresholds combined with predefined time points of serial sampling remains challenging in busy emergency settings with globally widely differing patients’ characteristics. Besides, in the context of suspected NSTEMI, clinicians do not interpret hs-cTn concentrations and thresholds in isolation, but in combination with ECG findings and clinical characteristics, such as chest pain onset time, cardiovascular risk factors, age, sex, and other comorbidities, which are largely neglected in most current diagnostic algorithms [8]. Thus, a diagnostic algorithm, simultaneously including various variables such as hs-cTn concentrations, their dynamic change during flexibly timed resampling, ECG findings as well as most relevant and immediately available clinical variables, constitutes an unmet clinical need in patients with suspected MI, both in the ED and in the ambulatory care setting.

Based on prior work [9], we derived and validated a machine-learning model, which estimates the individual probability of NSTEMI in patients presenting with symptoms indicative of MI. This model accounts for immediately available confounding clinical variables, allows for flexible timing of potential serial sampling and can be applied using most established hs-cTn assays, including point-of-care assays. We aimed to prove its clinical application in patients with suspected NSTEMI and [1] defined the model’s overall diagnostic accuracy, [2] assessed the clinical performance according to MI probability thresholds in heterogeneous clinical conditions, and [3] finally compared the model’s clinical utility against currently recommended assay-specific thresholds. Overall, this work shall pave the way towards the routine clinical implementation of medical decision support systems to improve a rapid, efficient and safe diagnostic process in patients with suspected MI.

## Methods

### Study design and populations

**Fig. 1:**
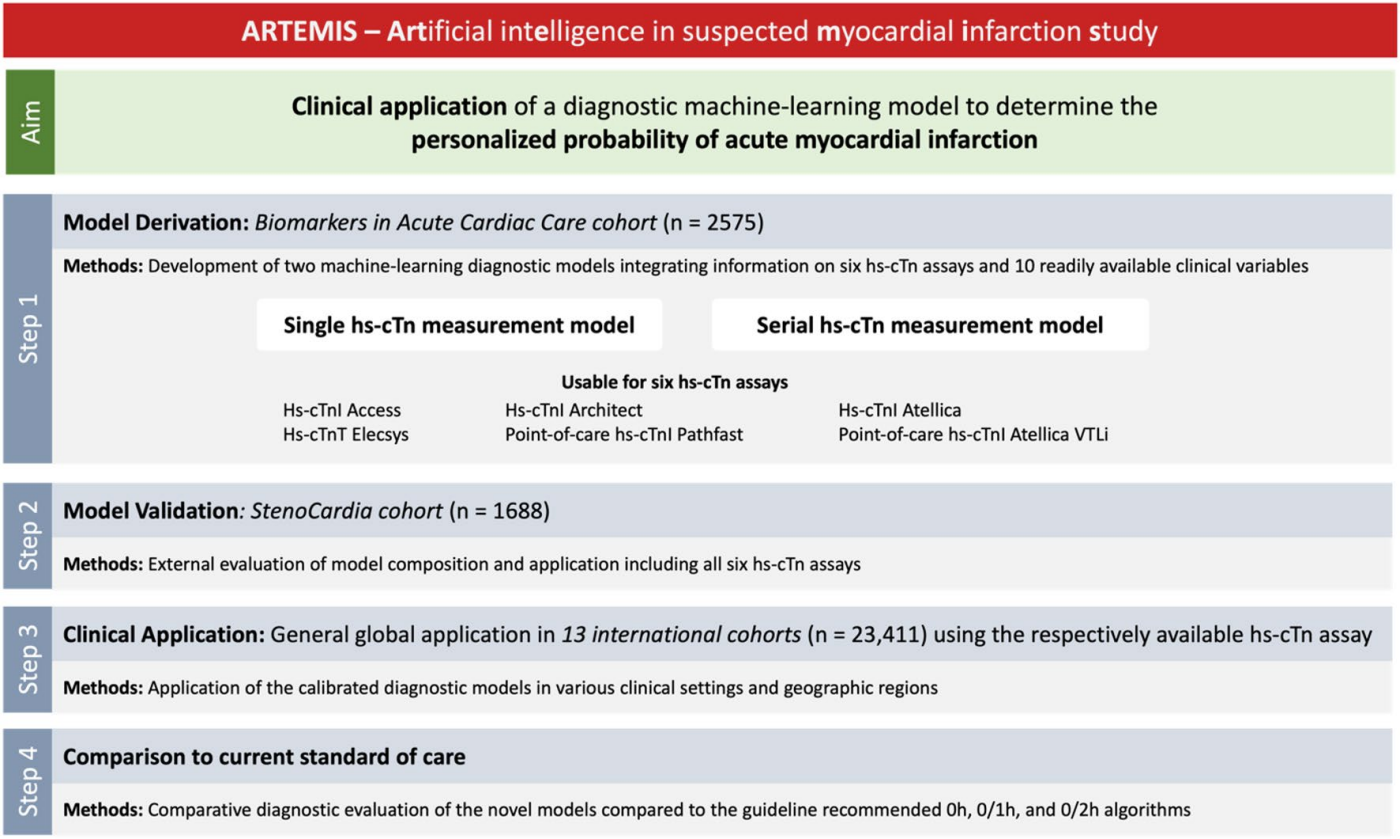
Study concept and diagnostic model development. This figure displays the overall study design including study populations, development of the diagnostic model, model validation and generalization, as well as comparison to the current standard of care

In the “Artificial intelligence in suspected myocardial infarction study “ (ARTEMIS), we derived and externally validated diagnostic models by estimating the probability of MI using machine learning (probability machines) in adult patients presenting to the ED with symptoms suggestive of MI. We excluded patients presenting with ST-segment elevation MI. The overall study concept is displayed in Fig. 1. Briefly, probability machines for MI were derived in the BACC (Biomarkers in Acute Cardiac Care; NCT02355457) study, which is an ongoing prospective observational diagnostic study performed at the University Heart & Vascular Center Hamburg, Germany [10, 11]. The probability machines were then externally validated in the stenoCardia (Study for Evaluation of New Onset Chest Pain and Rapid Diagnosis of Myocardial Necrosis; NCT03227159) cohort, which prospectively enrolled patients with suspected acute coronary syndrome at the EDs of the University Medical Center Mainz, the Federal Armed Forces Hospital Koblenz, and University Hospital Hamburg-Eppendorf between 2007 and 2009 in an observational fashion [12, 13]. To confirm the generalizability and global applicability of the newly developed and validated diagnostic models in clinically and geographically widely varying settings, anonymized individual-level data of thirteen additional cohorts from nine countries and four continents were transferred to the University Medical Center Hamburg-Eppendorf, Germany, to centrally apply the diagnostic models on the harmonized data in the global generalization dataset (see *Supplementary Appendix f*or detailed description).

All studies were carried out according to the principles of the Declaration of Helsinki and approved by the local ethics committees. Participation was voluntary; each patient gave written informed consent. The TRIPOD checklist for this study is provided in Table S1 in *Supplementary Appendix*.

### Adjudication of final diagnosis

The primary outcome of this study was the diagnosis of NSTEMI at time of ED presentation, which included type 1 and type 2 MI. In the derivation and validation dataset, the final diagnosis of MI was adjudicated after patient discharge by two cardiologists independently considering all available clinical, imaging, electrocardiographic and hs-cTn information. Cases in which the two initial adjudicators disagreed were reviewed by a third cardiologist. Detailed information on the adjudication process in each cohort including the generalization dataset may be found in the *Supplementary Appendix*.

### Outcome data

For prognostic evaluation, we collected data on incident MI, excluding the index events, as well as all-cause death within 30 days after ED presentation.

### Troponin measurements

Concentrations of cardiac troponin was measured by five hs-cTnI assays (Architect® i2000 platform by Abbott; Atellica® IM platform by Siemens Healthineers; Atellica VTLi® point-of-care device by Siemens Healthineers; Access® platform by Beckman Coulter; PATHFAST® Analyser by PHC) and one hs-cTnT assay (Elecsys® Cobas e411 platform by Roche Diagnostics) in blood samples collected at time of ED presentation and serially thereafter as part of routine clinical care or in batches of samples that had been stored at  – 80 °C. Targeted timing of the second blood draw differed between the various participating studies and ranged from one to three hours. Time elapsed between serial study blood sampling in the ED was documented. Additional information regarding the hs-cTn assays used in all ARTEMIS study cohorts is provided in the *Supplementary Appendix*.

### Clinical variables

In total, 18 patient-specific as well as hs-cTn-related variables readily available at time of ED presentation and all previously associated with myocardial infarction were considered for model development. The most important clinical variables were selected for the final model (see *Supplementary Appendix*).

### Statistical analysis and model development

A detailed statistical description is provided in the *Supplementary Appendix a*nd summarized in Figure S1. Briefly, for each of the six hs-cTn assays studied, we derived, validated, and globally applied two machine-learning diagnostic models, which estimate the individual probability of an acute MI in individuals presenting to the ED with suspected MI: One model was based on a single hs-cTn measurement obtained at time of ED presentation, the second model on two serial hs-cTn measurements. Modeling steps in the model derivation phase included multiple imputation of missing co-variables, cross-validation in all modeling and variable selection steps, and combination of multiple machines in a super learner with equal weights. Probability estimates of the super learner were calibrated in all validation and generalization studies.

The diagnostic performance of the models across the spectrum of possible MI probability thresholds was evaluated in one percent increments. Diagnostic performance measures were obtained from random effect meta-analyses and included negative and positive predictive value (NPV and PPV), sensitivity and specificity, proportion of patients below or above a given MI probability threshold as well as corresponding 30-day incidence of MI or death. Resulting tables and figures could be used to identify patients at low risk of MI suitable for outpatient management or those at high risk who are suitable to inpatient or invasive strategies. To illustrate the clinical applicability and to contrast the performance of the novel diagnostic model with the current state of the art approach, we compared the diagnostic performance measures of our diagnostic model with the 0 h, 0/1 h and 0/2 h strategy recommended by the ESC guideline [4].

To make the algorithm readily available and applicable to clinicians, a mobile application is currently constructed based on the present models, which are easily transferable to other systems. In a mid-term perspective, semi-automated integration of the diagnostic models into the local electronic health record systems as a medical support system is envisioned.

All statistical analyses were performed in R version 4.2.0 [14].

## Results

### Study populations

The models were developed in 2575 patients with suspected MI in the derivation cohort BACC and then applied in 1688 patients of the validation cohort stenoCardia as well as in 23,411 patients of the global generalization dataset. Baseline characteristics of the derivation, validation and global generalization cohorts can be found in Table 1 and Tables S2, S3, S4, S5. In the overall dataset, median age was 61 [50,73] years, 55.8% were male and 46.1% presented to the ED within the first three hours after symptom onset. Prevalence of MI ranged from 5.5 to 16.8% across the study cohorts. During follow-up, 643 (2.7%) incident cardiovascular death and 1007 Mis (4.8%) were observed.

**Table 1:** Baseline characteristics for derivation, validation, and generalization cohorts

	All patients	Derivation	Validation	Global generalization
Sample size	27,674	2575	1688	23,411
Age (years)	61.0 [50.0, 73.0]	64.0 [51.0, 75.0]	63.0 [52.0, 72.0]	61.0 [50.0, 73.0]
Sex (male) (%)	15,451 (55.8)	1638 (63.6)	1108 (65.6)	12,705 (54.3)
Heart rate (bpm)	76.0 [66.0, 88.0]	77.0 [67.0, 88.5]	70.0 [62.0, 81.0]	76.0 [66.0, 88.0]
Systolic BP (mmHg)	143.0 [128.0, 160.0]	147.0 [131.0, 163.0]	140.0 [129.0, 160.0]	143.0 [128.0, 160.0]
eGFR (mL/min for 1.73m^2^)	82.9 [63.4, 96.9]	76.9 [58.5, 92.3]	84.2 [69.0, 95.2]	83.4 [63.7, 97.4]
History of CAD (%)	8203 (29.8)	872 (33.9)	606 (36.9)	6725 (28.9)
History of heart failure (%)	2588 (11.5)	394 (15.3)	120 (7.5)	2074 (11.3)
History of atrial fibrillation (%)	1859 (13.0)	395 (15.3)	162 (9.8)	1302 (12.9)
Hypertension (%)	16,127 (59.0)	1681 (65.5)	1256 (74.4)	13,190 (57.1)
Hyperlipoproteinemia (%)	12,837 (48.4)	904 (35.1)	1236 (73.2)	10,697 (48.1)
Diabetes (%)	5404 (19.8)	326 (12.8)	303 (18.2)	4775 (20.7)
Ever smoker (%)	10,796 (43.1)	1187 (46.8)	865 (52.6)	8744 (42.0)
Family history of CAD (%)	8476 (40.0)	478 (19.3)	540 (33.2)	7458 (43.7)
Ischemic signs ECG (%)	4428 (18.4)	520 (20.8)	872 (52.1)	3036 (15.3)
Symptom onset < 3 h (%)	11,122 (46.1)	713 (29.4)	631 (37.4)	9778 (48.9)
Time between serial samples (min)	80.0 [60.0, 155.0]	60.0 [60.0, 63.0]	180.0 [162.0, 190.0]	90.0 [60.0, 148.0]
Final diagnosis of NSTEMI (%)	3249 (11.7)	368 (14.3)	283 (16.8)	2598 (11.1)
Follow-up cardiovascular death (%)	643 (2.7)	74 (3.4)	38 (2.3)	531 (2.7)
Follow-up MI (%)	1007 (4.8)	24 (1.1)	47 (2.8)	936 (5.5)
Hs-cTnI Access- First measurement (ng/L)	3.5 [2.3, 8.3]	5.3 [2.9, 15.8]	5.2 [2.3, 23.1]	3.0 [2.3, 6.0]
Hs-cTnI Access—Second measurement (ng/L)	4.0 [2.3, 10.3]	5.8 [3.0, 19.7]	7.2 [3.2, 38.6]	3.0 [2.3, 6.5]
Hs-cTnI Architect- First measurement (ng/L)	4.5 [2.0, 14.0]	5.7 [2.6, 16.1]	6.9 [3.5, 28.8]	4.0 [2.0, 12.0]
Hs-cTnI Architect—Second measurement (ng/L)	5.0 [2.2, 16.3]	5.9 [2.6, 19.4]	7.8 [3.6, 35.8]	4.1 [2.0, 14.0]
Hs-cTnI Atellica—First measurement (ng/L)	5.3 [2.5, 17.4]	5.7 [2.5, 19.9]	6.4 [3.0, 31.2]	4.8 [2.5, 14.6]
Hs-cTnI Atellica- Second measurement (ng/L)	6.4 [2.9, 22.0]	6.2 [2.7, 23.9]	8.0 [3.5, 41.2]	6.0 [2.8, 18.8]
Hs-cTnI Atellica VTLi- First measurement (ng/L)	7.6 [4.1, 16.0]	6.3 [3.8, 11.5]	-	7.8 [4.1, 16.8]
Hs-cTnI Atellica VTLi- Second measurement (ng/L)	7.9 [4.1, 17.2]	6.0 [3.8, 12.5]	-	8.2 [4.2, 18.4]
Hs-cTnT Elecsys—First measurement (ng/L)	9.0 [5.0, 20.0]	9.0 [5.0, 21.0]	9.2 [5.0, 20.3]	8.6 [5.0, 20.0]
Hs-cTnT Elecsys—Second measurement (ng/L)	9.0 [5.0, 22.0]	9.0 [5.0, 23.0]	8.1 [4.1, 23.3]	8.8 [5.0, 22.0]
Hs-cTnI Pathfast- First measurement (ng/L)	4.0 [2.3, 12.4]	3.7 [2.3, 12.2]	4.2 [2.3, 12.8]	-
Hs-cTnI Pathfast- Second measurement (ng/L)	4.3 [2.3, 15.5]	4.0 [2.3, 14.4]	5.1 [2.6, 20.3]	-

Data are presented as median [Q1, Q3] or number (proportion). *VTLi measurements were performed in a separate population of patients recruited to the BACC study. Detailed characteristics of these patients is provided in Table S2. Abbreviations: *BP*  blood pressure, *eGFR*  estimated glomerular filtration rate, *CAD*  coronary artery disease, *ECG * electrocardiogram, *MI*  myocardial infarction, *hs-cTn*  high-sensitivity cardiac troponin

**Fig. 2:**
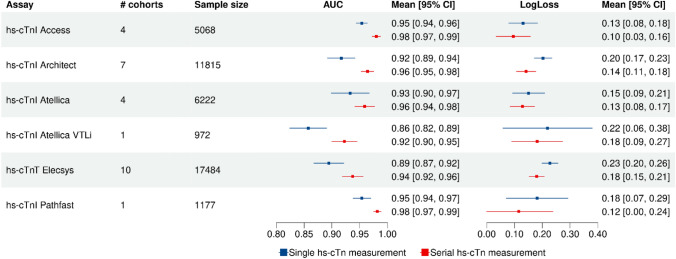
Discrimination measures using the diagnostic model based on a single and on a serial hs-cTn measurement per assay summarized across the validation and generalization cohorts. This figure summarizes the discrimination measures AUC and LogLoss with 95% CI for each hs-cTn assay using the diagnostic model with single and serial hs-cTn measurements. The displayed measured represent the summarized values from the validation and generalization cohorts. Detailed results from each cohort are displayed in Figure S5. Abbreviations: *AUC*  area under the curve, *CI*  confidence interval, *hs-cTn*  high-sensitivity troponin

Serial measurements of all hs-cTn assays were available in the derivation dataset, but availability of measurements varied among the validation and generalization cohorts (Figure S1). Overall, at time of ED presentation, hs-cTnT Elecsys was the most widely used assay with measurements available in 20,001 patients followed by hs-cTnI Architect in 14,255, hs-cTnI Atellica in 8332, hs-cTnI Access in 6946, hs-cTnI Pathfast in 3246 and hs-cTnI Atellica VTLi in 1088 patients Fig. 2.

### Model derivation

**Fig. 3:**
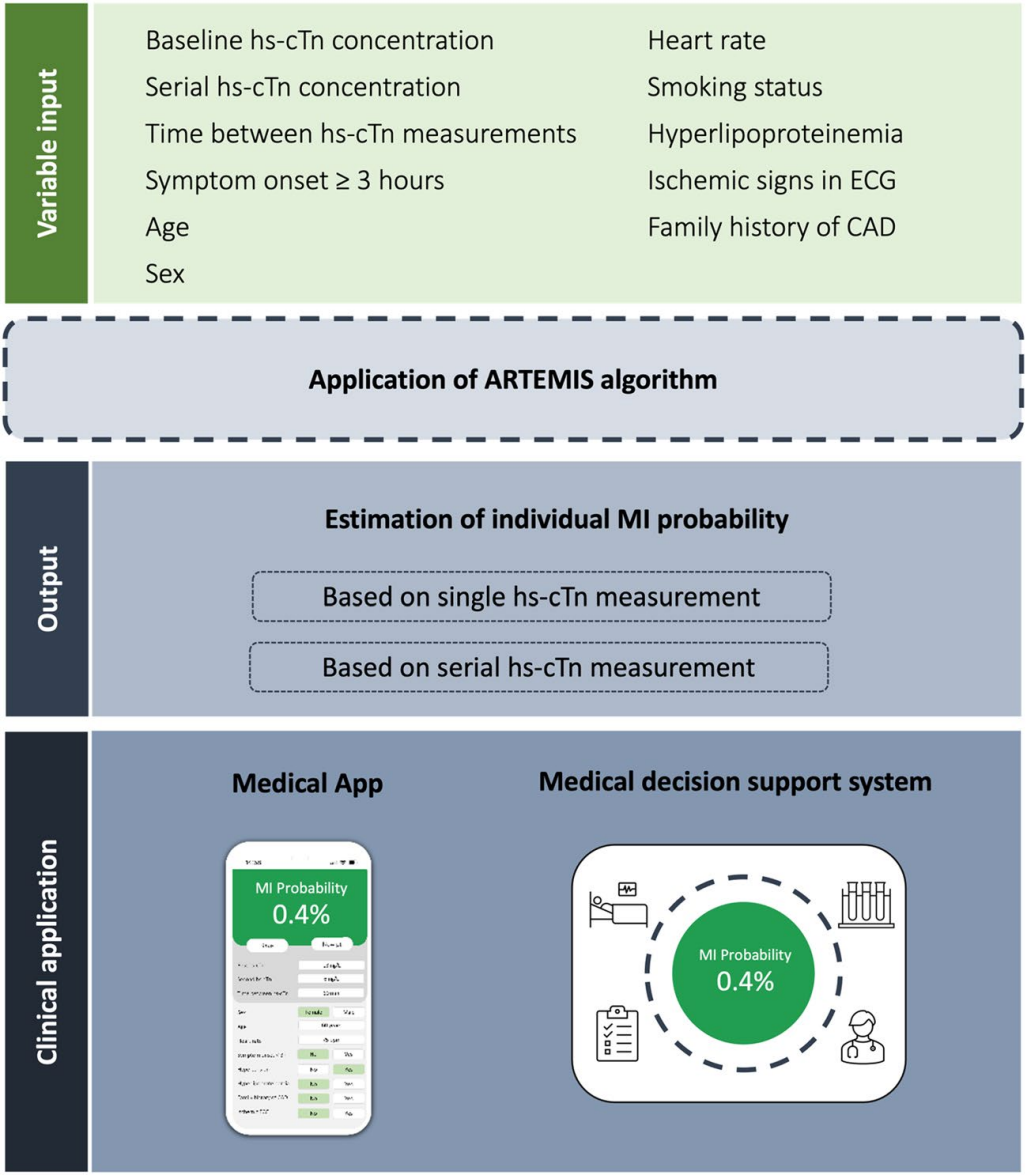
Diagnostic pathway in patients with suspected myocardial infarction—the machine-learning supported clinical application. This figure displays the clinical workflow to estimate the individual MI probability using the ARTEMIS diagnostic model. Abbreviations: *CAD*  coronary artery disease, *ECG*  electrocardiogram, *MI * myocardial infarction, *hs-cTn*  high-sensitivity cardiac troponin

Among 18 variables investigated, 9 variables for the single hs-cTn measurement and 8 variables for the serial hs-cTn measurement were selected (Table S6, Fig. 3). Based on these variables, four different learning machines were selected and combined to a super learner into each diagnostic model: For the single hs-cTn diagnostic model multivariable logistic regression with restricted cubic splines, gradient boosting, multivariate adaptive regression splines and elastic net were selected. For the serial hs-cTn diagnostic model multivariable logistic regression with restricted cubic splines, gradient boosting, multivariate adaptive regression splines and random forest were selected. Both diagnostic models provided a better performance compared to models based on hs-cTn alone, models including information on eGFR, or the full models (Figures S2, S3, S4). The machine-learning-based super learner outperformed classical multiple logistic regression for both the single and serial validation models (Figure S3). Specifically, it performed better than any single machine for the single hs-cTn troponin measurements. The diagnostic model using single or serial hs-cTn measurements showed high discriminative accuracies for each evaluated troponin assay (Figure S5).

### Model validation

In the validation dataset, the diagnostic model showed a better performance, compared to models based on hs-cTn alone, models including information on eGFR or a model including all offered clinical variables (Figures S2, S3, S4). Observed and predicted risks of MI were for all assays in the derivation data and after calibration in the validation data (Figure S6). When applying the diagnostic model based on a single or a serial hs-cTn measurement in the validation dataset, we observed an increase in AUC and a decrease in logLoss and Brier Score (Figure S5).

### Global generalization

In the global generalization dataset, observed and predicted risks of MI were again similar for all assays after re-calibration (Figure S7). The discriminative accuracy using the diagnostic model was high across all cohorts (Figure S5; Table S7). When summarizing the measures across the validation and generalization cohorts, the AUCs were similar for all hs-cTn assays applied (Fig. 2). In detail, the AUCs were 0.95 (95%CI 0.94–0.96) and 0.98 (95%CI 0.97–0.99) for the single and serial hs-cTn diagnostic model using the Access assay, and 0.92 (95%CI 0.89–0.94) and 0.96 (95%CI 0.95–0.98), for the Architect assay, respectively. For the Atellica assay, the AUC was 0.93 (95%CI 0.90–0.97) and 0.96 (95%CI 0.94–0.98), and 0.86 (95%CI 0.82–0.89) and 0.92 (95%CI 0.90–0.95), for the Atellica VTLi point-of-care assay, respectively. For the Elecsys assay, the AUC was 0.89 (95%CI 0.87–0.92) and 0.94 (95%CI 0.92–0.96) and the patient-near Pathfast assay revealed an AUC of 0.95 (95%CI 0.94–0.97) and 0.98 (95%CI 0.97–0.99), respectively.

### Clinical application

To illustrate the clinical usability, we calculated the diagnostic measures for each possible MI probability threshold. Across the range of thresholds, we observed a decreasing NPV and sensitivity with increasing MI probability, while PPV, specificity and 30-day mortality continuously increased (Figure S8, Tables S8, S9). As examples, the diagnostic measures to rule-out MI in individuals with a MI probability below 0.5%, below 1% and below 2% are depicted in Table 2 using both diagnostic models with single and serial hs-cTn measurements. When using single hs-cTn measurement and a MI probability of less than 0.5%, we observed very high NPVs of 99.6% or greater. In contrast, when using serial hs-cTn measurement and a MI probability of, e.g., less than 2%, we observed excellent diagnostic measures with NPV values of 99.5% or above and a proportion of at least 60% of the population. Importantly, these values were associated with a low risk of 30-day mortality ranging between 0.6–1.1%.

**Table 2:** Diagnostic measures of selected MI probability thresholds to rule-out of MI

MI probability < 0.5%	Single hs-cTn measurement model				Serial hs-cTn measurement model			
Hs-cTn assay	NPV (95%CI)	Sensitivity (95%CI)	Proportion (95%CI)	30d mortality (95%)	NPV (95%CI)	Sensitivity (95%CI)	Proportion (95%CI)	30d mortality (95%CI)
Access	99.7 (99.4, 99.8)	98.7 (98.3, 99.1)	44.4 (31.7, 57.9)	0.5 (0.2, 1.5)	99.8 (99.6, 99.9)	98.6 (98.0, 99.1)	67.5 (63.3, 71.4)	0.5 (0.2, 1.4)
Architect	99.6 (99.4, 99.8)	99.2 (97.4, 99.7)	30.0 (15.9, 49.4)	0.1 (0.0, 0.5)	99.8 (99.6, 99.9)	98.9 (97.6, 99.5)	59.5 (53.6, 65.1)	0.5 (0.3, 0.9)
Atellica	99.8 (99.6, 99.9)	99.1 (95.4, 99.8)	49.5 (41.5, 57.5)	0.3 (0.1, 0.6)	99.8 (99.6, 99.9)	98.8 (97.3, 99.5)	52.0 (42.7, 61.2)	0.4 (0.2, 0.7)
Atellica VTLi	99.7 (99.3, 100.0)	98.9 (98.2, 99.5)	32.5 (29.5, 35.4)	0.0 (NaN, NaN)	100.0 (NaN, NaN)	100.0 (NaN, NaN)	41.0 (37.8, 44.1)	0.5 (0.1, 0.9)
Elecsys	99.6 (99.2, 99.8)	99.1 (97.9, 99.6)	30.8 (20.8, 42.9)	0.3 (0.1, 1.2)	99.7 (99.3, 99.9)	99.0 (98.1, 99.5)	46.9 (32.3, 62.2)	0.6 (0.2, 1.6)
Pathfast	99.7 (99.4, 100.0)	99.4 (99.0, 99.8)	26.8 (24.3, 29.4)	0.3 (0.0, 0.6)	99.9 (99.7, 100.0)	99.4 (99.0, 99.8)	63.9 (61.1, 66.6)	0.4 (0.0, 0.8)

MI probability < 1%	Single hs-cTn measurement model				Serial hs-cTn measurement model			
Hs-cTn assay	NPV (95%CI)	Sensitivity (95%CI)	Proportion (95%CI)	30d mortality (95%)	NPV (95%CI)	Sensitivity (95%CI)	Proportion (95%CI)	30d mortality (95%CI)
Access	99.7 (99.5, 99.8)	98.2 (97.0, 98.9)	51.9 (38.8, 64.8)	0.4 (0.1, 1.3)	99.8 (99.5, 99.9)	98.3 (97.7, 98.8)	72.1 (68.5, 75.5)	0.7 (0.3, 1.7)
Architect	99.6 (99.3, 99.7)	98.5 (97.4, 99.2)	43.8 (34.4, 53.8)	0.2 (0.1, 0.5)	99.7 (99.5, 99.8)	98.6 (97.1, 99.3)	66.3 (58.6, 73.2)	0.7 (0.5, 1.0)
Atellica	99.8 (99.5, 99.9)	98.5 (95.4, 99.6)	54.9 (44.3, 65.2)	0.4 (0.1, 1.0)	99.8 (99.6, 99.9)	98.7 (96.5, 99.6)	59.9 (51.2, 68.0)	0.6 (0.3, 1.0)
Atellica VTLi	99.7 (99.4, 100.0)	98.9 (98.2, 99.5)	35.8 (32.8, 38.8)	0.3 (0.0, 0.6)	100.0 (NaN, NaN)	100.0 (NaN, NaN)	51.5 (48.3, 54.7)	0.6 (0.1, 1.1)
Elecsys	99.6 (99.3, 99.8)	98.5 (96.6, 99.4)	41.0 (31.1, 51.8)	0.4 (0.1, 1.8)	99.7 (99.3, 99.9)	98.7 (97.3, 99.4)	58.3 (46.3, 69.4)	0.7 (0.3, 1.9)
Pathfast	99.4 (98.7, 99.7)	97.6 (96.6, 98.4)	33.6 (7.7, 75.6)	0.3 (0.2, 0.5)	99.7 (99.4, 99.8)	99.2 (98.3, 99.6)	45.5 (11.0, 85.0)	0.5 (0.3, 0.8)

MI probability < 2%	Single hs-cTn measurement model				Serial hs-cTn measurement model			
Hs-cTn assay	NPV (95%CI)	Sensitivity (95%CI)	Proportion (95%CI)	30d mortality (95%)	NPV (95%CI)	Sensitivity (95%CI)	Proportion (95%CI)	30d mortality (95%CI)
Access	99.6 (99.3, 99.7)	97.2 (95.3, 98.4)	60.9 (47.9, 72.5)	0.3 (0.1, 1.2)	99.7 (99.5, 99.9)	98.0 (97.4, 98.5)	76.0 (72.0, 79.6)	1.1 (0.5, 2.2)
Architect	99.3 (99.0, 99.5)	97.1 (96.3, 97.8)	54.8 (45.3, 64.0)	0.4 (0.1, 1.3)	99.6 (99.4, 99.8)	98.3 (96.7, 99.1)	71.3 (64.0, 77.6)	0.7 (0.5, 1.0)
Atellica	99.6 (99.4, 99.8)	97.5 (90.2, 99.4)	60.7 (47.3, 72.6)	0.4 (0.2, 0.8)	99.7 (99.5, 99.8)	97.9 (94.6, 99.2)	67.3 (58.6, 74.9)	0.7 (0.5, 1.1)
Atellica VTLi	99.5 (99.0, 99.9)	97.7 (96.8, 98.7)	39.9 (36.8, 43.0)	0.5 (0.1, 1.0)	99.7 (99.3, 100.0)	97.9 (96.7, 99.0)	59.7 (56.5, 62.8)	0.6 (0.1, 1.1)
Elecsys	99.1 (97.7, 99.6)	96.5 (94.6, 97.8)	48.5 (35.1, 62.2)	0.5 (0.2, 1.2)	99.5 (99.3, 99.7)	97.6 (95.1, 98.9)	65.6 (55.3, 74.6)	0.9 (0.4, 2.2)
Pathfast	99.2 (98.8, 99.4)	97.6 (95.0, 98.8)	46.3 (17.0, 78.4)	0.5 (0.3, 0.8)	99.5 (99.1, 99.7)	98.1 (97.6, 98.5)	61.0 (32.9, 83.3)	0.6 (0.4, 0.9)

This table displays the diagnostic performance measures (NPV, proportion of individuals and 30-day mortality) using the MI probability as threshold. This table is based on data from the validation cohort stenocardia as well as the generalization cohorts ADAPT-BSN, ADPs-CH, FASTEST, LUND, RAPID-CPU, SAMIE, SEIGE & SAFETY, STOP-CP and UTROPIA. Abbreviations: *MI*  myocardial infarction, *hs-cTn*  high-sensitivity cardiac troponin, *NPV*  negative predictive value, *CI*  confidence interval, *NaN*  not a number

### Comparison to standard of care

Comparative analyses using a single hs-cTn measurement approach based on the ESC algorithms versus the ARTEMIS pathway are depicted in Table 3. Using the ARTEMIS pathway and considering an MI probability threshold < 0.5% to identify subjects eligible for direct rule-out of MI, the safety, quantified by NPV and sensitivity, was very high and similar when compared to the direct rule-out approach of the ESC algorithms. Importantly, however, the proportion of patients qualifying for direct and safe rule-out based on a single hs-cTn measurement was increased by factor two–three by our machine-based model, ranging between 30 and 49%, as compared to 14 and 15% using the direct rule-out approach provided by the ESC algorithms. Using an MI probability of > 50% as a direct rule-in criteria, high accuracy, quantified by the PPV and specificity, was achieved. The accuracy and proportions of direct rule-in were similar to the ESC algorithms. Furthermore, the observational zone after a single hs-cTn measurement was reduced for all hs-cTn assays by 10–33% when using the ARTEMIS pathway. For the serial hs-cTn measurement approach, a selection of possible ARTEMIS thresholds to define rule-out and rule-in of MI resulted in overall comparable diagnostic performances when directly compared to the ESC 0/1 h and 0/2 h algorithms (Table S10).

**Table 3:**
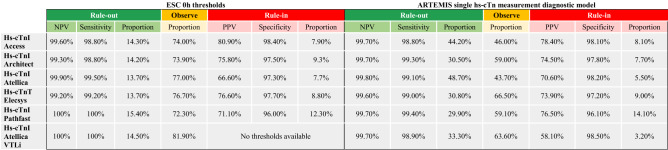
Diagnostic performance comparison of the direct rule-out or rule-in approach based on a single hs-cTn measurement of the ESC 0/1 h algorithms and the ARTEMIS diagnostic model

This table compares the diagnostic performance to directly rule-out or to rule-in MI using a single hs-cTn measurement with the ESC 0/1 h algorithms and the ARTEMIS diagnostic model. Global and cohort specific imputation of the necessary variables for ARTEMIS, with the exclusion of troponin measurements, was performed. Due to the meta-analytic background of the analyses, the proportions of rule-out, observe and rule-in zone due not sum up to 100%. Using the ARTEMIS model an MI probability < 0.5% to rule-out MI and MI probability > 50% to rule-in MI was used. Abbreviations: *MI*  myocardial infarction, *hs-cTn * high-sensitivity cardiac troponin, *NPV*  negative predictive value, *PPV*  positive predictive value

### Exemplary clinical use cases

The general workflow and the potential clinical application of the ARTEMIS pathway are displayed in Fig. 3 and Supplementary Appendix (Figure S10). The smart interpretation of cardiac troponin, which can be measured with a large variety of possible hs-cTn assays in ARTEMIS, in combination with other easily available clinical variables may inform the treating physicians in real time about the individual probability of MI in form of a mobile application or, if embedded in the local electronical medical health record system, as a medical decision support system. Hereby, ARTEMIS may guide safe, efficient and immediate medical decision in patients presenting with suspicion of MI.

## Discussion

Extending prior work [9], we derived, validated, and generalized a personalized diagnostic model to immediately, accurately, and safely quantify the risk probability of MI. From individual-level data contributed by more than 27,000 patients with suspected acute MI in four continents, nine countries and 14 prospectively established real world cohorts we applied various machine-based learning tools and developed a super learner model resulting in two diagnostic models. Their clinical application allows providers to determine the probability of MI with high diagnostic accuracy. The personalized model (1) works irrespective of which hs-cTn assay is used, (2) integrates the information of important and rapidly available clinical variables, (3) requires neither assay-specific cut-offs nor fixed timing of serial sampling, (4) can be applied after calibration in various clinical settings with widely varying pre-test probabilities and (5) offers a selection of risk probability thresholds (e.g., 0.5%, 1% or 2% MI probability) which allows for safe and immediate discharge in a very high proportion of patients.

While the application of hs-cTn assays improves visibility of even minor myocardial injury and allows for early detection of MI, the clinical management and decision-making became more challenging [4, 13, 15]. Consequently, various assay-specific hs-cTn algorithms have been developed and implemented to efficiently diagnose and triage patients with suspected MI [16–18]. Although these algorithms allow for major advances in rapid and safe clinical decision-making, they still rely on inflexible rules for the timing of hs-cTn resampling (1, 2 or 3 h) and apply assay-specific thresholds of mostly very low concentrations and do not account for clinical variables such as age, sex, risk factors, chest pain onset time, and others. In consequence, the assay-specific 0/1 h and 0/2 h or 0/3 h algorithms as suggested by the European Society of Cardiology for example, are not fully implemented in global clinical routine [4].

To accelerate the advantage of hs-cTn usage in clinical routine and enable—in interaction with hs-cTn point-of-care tests—a safe application also in ambulatory settings, we extend the concept of risk probabilities introduced recently [9] towards a highly accurate personalized diagnostic model. As the model was trained using eleven (selected out of an initial 18) clinical variables including time of chest pain onset, time between serial sampling, ECG, age, sex, and cardiovascular risk factors and nearly all hs-cTn tests currently available, it provides the highest possible diagnostic accuracy and allows for rapid and safe decision-making. Both, single and serial sampling models achieve excellent diagnostic accuracy and offer the opportunity to select rule-out thresholds which allow rapid and safe discharge in a high proportion of patients. To achieve the best balance between high safety and high efficacy, a low MI probability threshold (e.g., 0.5%, 1% or 2%) is recommended for rule-out after single or serial testing, respectively. Compared with previous data on the performance of the ESC 0/1 h algorithm reporting a rule-out proportion of 44–57%, the rule-out proportions achieved by the application of the thresholds of the diagnostic models are larger and range, e.g., for a serial rule-out cut-off < 2%, between 60 and 76% [18, 19]. This improvement is most apparent for a single measurement approach, which allows direct rule-out of MI in 30–49% of the overall population compared to 13–15% using the ESC algorithm [18–22].

As the model is based on heterogenous global data, it is calibrated for European, Australian, New Zealand, Northern American, and Japanese conditions and, therefore, can be generally applied. The model also integrates two point-of-care hs-cTn assays (Pathfast and Atellica VTLi). When hs-cTn point-of-care assays are used, the ARTEMIS model can be applied in outpatient settings and, therefore, might improve diagnostic accuracy and speed in outpatient care and could reduce the number of hospital admissions.

In general, machine-based learning diagnostic and prediction models need to fulfill high methodological, clinical and regulatory standards before being used by healthcare professionals in clinical practice [23]. A recent report raises 12 critical questions, all of which have been positively addressed by the current algorithm [23]. In particular, the sample size is appropriate, validation has been extensively performed, and the outcome variable is labeled reliable, replicable, and independent.

Prior work already introduced machine-learning concepts to provide an individualized and objective assessment of the likelihood of myocardial infarction [24]. It for the first time presented the concept of machine-based learning to improve the diagnostic accuracy of MI diagnosis and rule-out. Although this work paved the way towards modern diagnostic approaches and performs well in routine clinical practice [25], it relies on only two predefined clinical variables age and sex beyond hs-cTn, and it is restricted to one specific hs-cTnI assay. It further highlights the need for model calibration prior to application in the population, which was limited in this the first concept [25]. The ARTEMIS model had been calibrated for the heterogeneous clinical conditions globally but requires further calibration of the super learner for each clinical setting, in which it will be directly applied. In consequence, the concept and construction of the ARTEMIS model will enable both, the inclusion of any hs-cTn assay entering the market and local calibration to settings in which it will be clinically applied.

The integration of the selected, easily available variables including whatever hs-cTn test available, supports an app- or middleware-guided safe, efficient and immediate medical decision. Whereas the ARTEMIS pathway might be suitable for embedded middleware approaches, which enable the integration into the hospital-based electronic health record system, app-based solutions might be more suitable for ambulatory care or independent emergency settings.

Some limitations should be considered when interpreting the findings. First, the outcome diagnoses of MI were adjudicated in each cohort separately and were not based on a harmonized standard operating procedure. Second, our models were validated to estimate the individual risk of MI in patients with clinically suspected MI. This does not include other acute conditions, that may lead to acute chest pain, such as pulmonary embolism or aortic dissection. Therefore, the estimated MI probabilities must always be considered in the clinical context and should not be used as only basis for decision-making. Finally, our diagnostic models were derived, validated, and generalized using data from multiple prospective, diagnostic studies, but have not been prospectively tested in clinical routine. Therefore, to assess real-world performance not only in the ED but also in other clinical settings (e.g., in ambulatory care or in the preclinical setting in ambulances), prospective clinical trials directly applying the ARTEMIS diagnostic model and comparing against standard of care is of importance.

In conclusion, we developed, validated, and globally applied the easily applicable diagnostic ARTEMIS model considering immediately available variables to estimate the individual risk of MI in patients with suspected MI. The model can be used with most hs-cTn assays currently available and allows for rapid and safe discharge of a very high proportion of patients. Its digital application might improve routine clinical practice globally and enable a personalized diagnostic evaluation of suspected MI.

## Supplementary Information

Below is the link to the electronic supplementary material.

Supplementary file1: Table S7 (XLSX 64 KB)

Supplementary file2: Table S8 (XLSX 79 KB)

Supplementary file3: Table S9 (XLSX 78 KB)

Supplementary file4: Methods and Statistical Analyis (PDF 242 KB)

Supplementary file5: Supplementary Figures an tables (PDF 3514 KB)

## Data Availability

Due to study-specific regulations of each cohort dataset, individual level data may not be shared. Qualified researchers may contact the corresponding author to discuss potential options. For the derivation and validation cohorts, de-identified data may be made available upon request.
