# Supplementary material for: Personalized diagnosis in suspected myocardial infarction
Source: Clin Res Cardiol. 2023 May 2;112(9):1288–301. doi: 10.1007/s00392-023-02206-3 (PMC10449973; doi:10.1007/s00392-023-02206-3)
Supplement: Supplementary file 4 — Supplementary file4: Methods and Statistical Analyis (PDF 242 KB) [file 392_2023_2206_MOESM4_ESM.pdf]

Personalized diagnosis in suspected myocardial infarction

Supplementary Material:

Methods and Statistical Analysis

January 03, 2023

Version 4.3

Table of contents

TABLE OF CONTENTS 2

1. BRIEF OVERVIEW ON MODEL DEVELOPMENT AND VALIDATION 4

2. SOURCE OF DATA 5

2.1 MODEL DERIVATION DATA 5
2.2 EXTERNAL VALIDATION DATA 5
2.3 GENERALIZATION DATA 6
2.3.1 ADAPT-BSN 6
2.3.2 ADPS-CH 7
2.3.3 DROP-ACS 7
2.3.4 FASTEST 8
2.3.5 HIGH-STEACS 8
2.3.6 LUND 8
2.3.7 RAPID-CPU 9
2.3.8 ROMI 9
2.3.9 SAMIE 9
2.3.10 SEIGE AND SAFETY 10
2.3.11 STOP-CP 10
2.3.12 UTROPIA 10

3. VARIABLES USED FOR ANALYSES 11

3.1 OUTCOME VARIABLE 11
3.2 INDEPENDENT VARIABLES (CANDIDATE FEATURES) 11

4. MODEL DEVELOPMENT (DERIVATION) 13

5. VALIDATION OF MODEL 17

6. CALIBRATION 17

6.1 PROBABILITY ESTIMATION – UNCALIBRATED 17
6.2 PROBABILITY ESTIMATION – CALIBRATED 17
6.2.1 LOGISTIC REGRESSION WITH RESTRICTED CUBIC SPLINES (RCS) (LOGREG-RCS) 17

6.2.2 Logistic regression (logreg) 17
6.2.3 Elkan's approach 17

7. GENERALIZATION OF MODEL 18

7.1 Multiple imputation 18
7.1.1 Imputation when all variables were present 18
7.1.2 Imputation in the presence of missing variables 18
7.2 Performance measures 19
7.3 Classification performance 19
7.4 Meta-analysis: pooling of estimates across studies 19
7.5 Plots 19
7.6 Incorporation of the Siemens Atellica VTLi 19

8. SAMPLE SIZE CONSIDERATIONS 19

9. CALCULATION OF THE VARIANCE OF THE AUC 20

10. REFERENCES 22

1. Brief overview on model development and validation

Machine learning models were developed, validated, and generalized to estimate the individual probability of myocardial infarction (MI) in patients presenting to the emergency department (ED) having symptoms potentially indicative of MI. Separate models were estimated for each of the 6 investigated hs-cTn assays twice.

- Single hs-cTn measurement: the model uses troponin information from the first troponin measurement only plus patient-specific data.
- Serial hs-cTn measurement: the model uses troponin information from the first troponin measurement and a second troponin measurement with the same assay obtained at a later time point during ED stay plus patient-specific data.

Details of the estimation approach is provided in the subsequent sections of this supplement. In brief:

- All modeling steps were done with 10-fold cross-validation.
- We first imputed missing data using multiple imputation.(1)
- We employed 11 different learning machines for each of the hs-cTn assays within each cross-validation for both single and serial hs-cTn measurements and a total of 18 clinical variables were initially offered (full models).
- A clinical variable was included if it was selected by at least 5 machines (reduced models). This was thought to improve model stability.
- Finally, we selected the four best-performing non-redundant machines for single hs-cTn measurement as well as the four best-performing non-redundant machines for serial hs-cTn measurements and combined them into a super learner with equal weights for single and serial hs-cTn measurements separately to estimate MI probability. Individual MI probabilities estimated by both super learners were expressed as a number ranging theoretically between 0-100%.
- The performance of the single and serial hs-cTn measurement super learner model was primarily assessed by logLoss (lower values indicate better performance) and, as secondary performance measures, by the AUC (higher values indicate better performance) as well as the Brier score (lower values indicate better performance).
- We compared
  - the AUC, Brier score and logLoss of the reduced machines to
  - the full machines and to
  - machines using only hs-cTn measures without other clinical variables and to
  - machines including estimated glomerular filtration rate (eGFR) using the CKD-EPI formula.(2)
- We compared single machines with super learner machines.
- Comparisons were done with the Iman and Davenport version of the Friedman test followed by the Nemenyi post-hoc test.(3)

- The single and serial hs-cTn measurement models were validated in the external validation dataset and the generalization datasets after re-calibration using logistic regression with restricted cubic splines. Calibration curves were generated to assess model calibration.
- DerSimonian and Laird random effect meta-analyses were estimated for averaging results between generalization data sets.(4)
- Tables and figures were created to demonstrate the diagnostic performance across the spectrum of possible MI probability thresholds in one percent increments. Diagnostic performance measures included negative and positive predictive value (NPV and PPV), sensitivity and specificity, proportion of patients below or above a given MI probability threshold as well as corresponding 30-day incidence of MI or death. These tables and figures could be used to identify patients at low risk of MI suitable for outpatient management or those at high risk who are suitable to inpatient or invasive strategies.
- Statistical analyses were performed in R version 4.2.0.(5)

2. Source of Data

The procedure was divided into three steps: 1. derivation, i.e., model development, 2. external validation, and 3. generalization. Five different troponin measurements were used for model development and in the cohort used for external validation. One, two or three different troponin assays were measured in the generalization cohorts. Measurements for a 6th troponin assay together with one of the other 5 troponin assays was available in an additional set of patients from the derivation cohort. Patients with ST-segment elevation MI were excluded from all parts of this study.

2.1 Model derivation data

The Biomarkers in Acute Cardiac Care (BACC) study (6) was used for model development. BACC has been described previously (6). Briefly, we included 2719 patients presenting to the ED and chest pain unit of the University Heart Center Hamburg with suspected acute MI. All patients were enrolled between July 2013 and December 2019. The inclusion criteria were a suspected MI, age ≥ 18 years and the ability to provide written informed consent. We excluded all patients with ST-elevation MI (n = 144) from further analyses. An additional n = 135 BACC patients without ST-elevation MI was included for the integration of an additional assay into the machine learning model. The diagnosis was adjudicated in a blinded fashion by two physicians independently according to the fourth Universal definition of MI. In cases of disagreement a third physician was consulted and disagreements resolved. The BACC study was registered at www.clinicaltrials.gov (NCT02355457).

2.2 External validation data

The stenoCardia study (7) was used for model validation. The methodology, follow-up, and adjudication of outcomes in stenoCardia have been reported in detail previously (7).

Between January 2007 and December 2008, 1818 patients with suspected acute coronary syndrome were consecutively enrolled in an observational multicentre cohort at three German tertiary care centers (University Medicine Mainz, University Hospital Hamburg-Eppendorf and Federal Armed Forces Hospital Koblenz). We excluded individuals with ST-segment-elevation MI (n=130) because the electrocardiographic diagnosis requires immediate treatment and biomarker diagnosis plays a minor role. The stenoCardia study was registered at www.clinicaltrials.gov (NCT03227159).

2.3 Generalization data

Generalization was done using the cohorts listed in Table 1. A brief description of each cohort is provided below.

Table 1: Cohorts used in the generalization step of this project

Name of cohort	Country of origin	Troponin assay(s) measured	Sample size
ADAPT-BSN (8) and IMPACT Summarized as ADAPT-BSN	Australia	Access hs-cTnI<br>Architect hs-cTnI<br>Elecsys hs-cTnT	2,315
ADAPT-CH (8), ADAPT-RCT (9), EDACS-RCT (10), SPACE-24 (11) Summarized as ADPs-CH	New Zealand	Access hs-cTnI<br>Architect hs-cTnI<br>Elecsys hs-cTnT	3,119
DROP-ACS (12)	Japan, Taiwan	Elecsys hs-cTnT	1,420
FASTEST	Sweden	Architect hs-cTnI<br>Elecsys hs-cTnT	1,233
High-STEACS (13)	Scotland	Architect hs-cTnI	1,750
LUND (14)	Sweden	Elecsys hs-cTnT	1,164
Rapid-CPU (15)	Germany	Elecsys hs-cTnT	4,938
ROMI (16)	Canada	Architect hs-cTnI<br>Elecsys hs-cTnT	1,366
SAMIE	Australia	Access hs-cTnI<br>Atellica hs-cTnI	1,985
SEIGE and SAFETY	United States of America	Architect hs-cTnI<br>Atellica hs-cTnI<br>Atellica VTLi hs-cTnI	1,033
STOP-CP (17)	United States of America	Elecsys hs-cTnT	1,457
UTROPIA (18)	United States of America	Architect hs-cTnI	1,631

2.3.1 ADAPT-BSN

The 2-Hour Accelerated Diagnostic Protocol to Assess Patients With Chest Pain Symptoms Using Contemporary Troponins as the Only Biomarker (ADAPT) and the IMPACT study were prospective studies performed in 2008 and 2011. The primary aim of both studies was to evaluate accelerated chest pain pathways in the assessment of acute coronary syndrome. All patients ≥ 18 years presenting with suspected acute coronary syndrome to the ED at the Royal Brisbane and Women's Hospital, Australia were included. The adjudication was performed by a physician in first instance and a second

cardiologist for all patients with a cardiac endpoint. It was based on all available clinical and imaging parameters up to 30 days. The troponin assay used to adjudicate patient outcomes was the Beckman-Coulter enhanced AccuTnI assay in routine use at the time. Both studies were registered at www.australianclinicaltrials.gov.au (ACTRN12611001069943 and ACTRN12611000206921).

2.3.2 ADPs-CH

The ADAPT-CH study was prospectively performed in accordance with the ADAPT-BSN study (see above). The ADAPT-RCT, registered at www.anzctr.org.au (ANZCTR12610000766011), aimed at comparing the effectiveness of a rapid diagnostic pathway with a standard-care diagnostic pathway for the assessment of patients with possible cardiac chest pain in a usual clinical practice setting. ED patients, where the attending physician was investigating for possible acute coronary syndrome, were included. Two senior clinicians adjudicated independently for any major adverse cardiac event within 30 days. A third senior clinician adjudicated any disagreements with the first two clinicians. The ED Assessment of Chest Pain Score – Randomized Controlled Trial (EDACS-RCT, registered at www.anzctr.org.au, ANZCTR12613000745741) aimed to test for the existence and size of any beneficial effect of using the EDACS-ADP protocol in routine clinical care compared with the ADAPT-ADP protocol. SPACE-24 was an observational study with additional sampling time points. Cohort selection and gold standard diagnosis for EDACS-ADP and SPACE-24 were identical to the ADAPT-RCT. All studies were initiated and based in Christchurch Hospital, New Zealand with majority of recruitment from this center.

2.3.3 DROP-ACS

The Diagnostics and Reduction of Asian Patients with Acute Coronary Syndrome Cost Analysis Base on the 0/1-h Algorithm Using High- sensitivity Cardiac Troponin (DROP-ACS) study is prospective, international, multicenter, diagnostic, cohort investigation conducted at five sites in two countries (Japan and Taiwan) that began in November 2014. Briefly, we included patients presenting to the ED and chest pain. All patients were enrolled until June 2022. The inclusion criterion was adults (aged 30–89 years) presenting with chest pain related to a suspected cardiac cause. Implementation of the 0/1-h algorithm was left to the discretion of the attending physicians. The exclusion criteria were as follows: (1) STEMI; (2) chronic kidney disease (serum creatinine level > 3 mg/dL); (3) congestive heart failure, defined as the presence of hypoxia and typical pulmonary congestion confirmed on a chest radiograph; (4) ventricular tachycardia. Informed consent was obtained from all patients. The gold standard diagnosis was adjudicated in a blinded fashion by two physicians independently according to the fourth Universal definition of MI. In cases of disagreement a third physician referred. The DROP-ACS study was registered at https://www.umin.ac.jp (UMIN000030668).

2.3.4 FASTEST

The "Fast ASsessment of Thoracic pain in the Emergency department using high-Sensitive Troponins" (FASTEST) study is a prospective multicenter study divided into two phases. The study was performed at six different Swedish centers enrolling unselected patients with acute chest pain presenting to the ED. The primary objective of the study was to determine whether a diagnostic strategy based on early serial measurement of high-sensitive troponins and a simple risk score will reduce the admission rate in patients with symptoms suggestive of ACS. The inclusion criteria were age ≥18 years; chest pain suggestive of ACS with duration of ≥10 min and onset of last episode within 12 hours; willingness to have extra blood samples taken; and written informed consent. The exclusion criteria were ST-segment elevation or new left bundle branch block. All patients underwent standard clinical assessment including repeated measurements of cTn, in phase 1 at ED presentation, 2 hours and 6–24 hours and in phase 2 at presentation, 1 hour and 6-24 hours. At five centers, the Elecsys Troponin T high-sensitive assay (Roche) was used and at one center, the ARCHITECT STAT High Sensitive Troponin-I assay (Abbott). Plasma samples for biobanking were obtained at presentation, after 2h (phase 1), 1h (phase 2) and 6– 24 hours. After discharge from the hospital, all patients were followed for cardiac events by telephone contacts and, if necessary, by validation in patient records at 30 days. No patient was lost to follow-up. The index diagnosis and cardiovascular events up to 30 days were adjudicated by independent reviewers.

2.3.5 High-STEACS

The High-Sensitive Troponin in the Evaluation of Acute Coronary Syndrome (High-STEACS) sub-study prospectively enrolled patients with suspected acute coronary syndrome presenting to the Royal Infirmary of Edinburgh, Edinburgh, Scotland (clinical trials registration at www.clinicaltrials.gov, NCT01852123). All patients provided written informed consent. Patients were excluded if they had a previous presentation during the study period or were not resident in Scotland. Patients with ST-elevation MI were not included. The final diagnosis was adjudicated for all patients by two independent physicians, with consensus from a third physician where there was discrepancy. Patients were classified as having type 1 MI, type 2 MI or myocardial injury in accordance with the third universal definition of MI. Any hs-TnI concentration above the sex-specific 99th centile upper reference limit (16ng/L for women, 34ng/L for men) was considered evidence of myocardial necrosis. All patients were followed for at least one year for subsequent MI or cardiac death.

2.3.6 LUND

The Lund Chest Pain Study included patients presenting with nontraumatic chest pain to the ED of Skåne University Hospital in Lund (clinical trials registration at www.clinicaltrials.gov, NCT05484544). In this prospective observational study, clinical data, 1h hs-TnT, and ED physicians' assessment of the patient history and ECG were collected. The primary objective was to evaluate the diagnostic accuracy of the 1h

algorithm when used in conjunction with patient history and ECG, to predict MACE within 30 days as compared to using 1h hs-TnT alone. The goldstandard was a final adjudicated diagnosis of 30-day MACE, as decided by independent reviews by two cardiologists, and in case of disagreement, by a third cardiologist. The cardiologists were blinded to the 1h hs-TnT. The adjudicated diagnoses were based on all available clinical information from all hospitals in Sweden within 60 days from the index visit, such as patient history and results of blood samples, ECG, echocardiography, stress test, and coronary angiography.

2.3.7 Rapid-CPU

The RAPID-CPU consists of two study populations (registration at www.clinicaltrials.gov, NCT03111862). Consecutive patients presenting to the ED at University hospital Heidelberg, Germany, with acute symptoms suggestive of acute coronary syndrome were included in a retrospective registry study. Consecutive patients with suspected acute coronary syndrome were recruited between 1 July 2016 and 30 June 2017 (cohort 1) and between 31 June and 1 July 2018 (cohort 2). In both populations, a final adjudicated diagnosis was made by two independent cardiologists. A third cardiologist refereed in cases of disagreement. The adjudication was based on all available clinical and imaging parameters (e.g. ECG, Echo, MRI, Angiography), as well as hs-cTnT. The criteria for the diagnosis of MI were based on the fourth universal MI definition.

2.3.8 ROMI

After approval from the research ethics board, the Optimal Troponin Cutoffs for acute coronary syndrome in the ED (ROMI-3: Rule-out MI 3h; registration at www.clinicaltrials.gov, NCT01994577) study prospectively enrolled consecutive adults (18 years or older) who presented to the ED with symptoms suggestive of ACS and for whom the ED physician ordered cardiac troponin. The adjudication process was led by an emergency physician with at least 2 of the study authors independently adjudicating the outcomes with disagreements not resolved by consensus referred to a third reviewer. All outcome adjudicators were blinded to the hs-cTn results, with MI in the study population defined as per the Third Universal Definition with the contemporary Abbott cTnI assay.

2.3.9 SAMIE

The Suspected acute MI in Emergency (SAMIE) study included 2022 patients presenting to the Emergency Department of five Australian hospitals. All patients were enrolled between November 2020 and September 2021. The inclusion criteria were age ≥ 18 years and the treating clinician investigated for acute MI. Exclusion criteria were all patients who presented with ST-elevation MI who were directly transferred for cardiac catheterization, patients transferred from another hospital, previous enrolment within 30 days, pregnancy, unwillingness or inability to provide informed consent, and recruitment was considered inappropriate (e.g., palliative patient). The gold standard diagnosis was adjudicated in a blinded fashion by one cardiologist to the fourth Universal definition of MI. A second cardiologist reviewed all cases of type 2 MI, with a third clinician reviewing any

discrepancies. The SAMIE study was registered at https://anzctr.org.au (ACTRN12621000053820).

2.3.10 SEIGE and SAFETY

The SEIGE and SAFETY studies are prospective, observational studies of consecutive patients presenting to the ED in whom hs-cTnI measurements were obtained. The purpose of the study is to evaluate the clinical performance of the Siemens Atellica VTLi point of care hs-cTnI test system (SEIGE) and the Attelica IM central laboratory hs-cTnI assay (SAFETY) for the diagnosis and rule out of MI in patients presenting to the emergency department in whom serial cTnI measurements are obtained on clinical indication. All participants were enrolled between October 2020 and October 2021 at the Hennepin County Medical Center (Minneapolis, MN, USA). The gold standard diagnosis was adjudicated in a blinded fashion by two physicians independently according to the fourth Universal Definition of MI. The study was registered at www.clinicaltrials.gov (NCT04772157).

2.3.11 STOP-CP

The High Sensitivity Cardiac Troponin T to Optimize Chest Pain Risk Stratification (STOP CP) study has been described previously(17). Briefly, the study included 1457 patients presenting to the ED across eight sites in the United States with suspected acute coronary syndrome. All patients were enrolled between July 2013 and December 2019. The inclusion criteria were suspected ACS, age ≥ 21 years and the ability to provide written informed consent. The following patients were excluded: Patients with ST-segment elevation MI at ED presentation, systolic blood pressure <90 mm Hg, a life expectancy of <90 days, a noncardiac illness requiring admission, lack of capacity to provide consent, inability to be contacted for follow-up, non–English speaking, pregnancy, and prior enrollment in the current study. The gold standard diagnosis was adjudicated in a blinded fashion by two physicians independently according to the fourth Universal definition of MI. In cases of disagreement a third physician referred. The STOP-CP study was registered at www.clinicaltrials.gov (NCT02984436).

2.3.12 UTROPIA

The Use of TROPonin In Acute coronary syndromes (UTROPIA) study is an observational cohort study performed at the Hennepin County Medical Center, Minneapolis, Minnesota, United States. Patients presenting to the emergency department within the defined study period (February 2014 to June 2016) were considered for inclusion. Participants were included, when they had two or more cTnI values ordered for any clinical indication with specimen available for hs cTnI assay, 18 years of age or older, ECG done on admission and agreed to disclosure research. All cases with at least one hs-cTnI >99th percentile were adjudicated according to the Third Universal Definition of MI consensus recommendations by two clinicians following review of all available medical records including 12-lead ECG, echocardiography, angiography, hs-cTnI concentrations, and clinical presentation. Cases with an adjudication discrepancy were reviewed and

adjudicated by a third senior clinician. The study was registered at www.clinicaltrials.gov (NCT02060760).

3. Variables used for analyses

3.1 Outcome variable

The primary outcome was acute Non-ST-elevation MI at index presentation (yes/no). Individuals without a diagnosis were excluded.

Secondary outcome was the 30-day incidence of death or MI.

3.2 Independent variables (candidate features)

Both troponin-related variables (features; see Troponin measurements were log-transformed using the natural logarithm to reduce skewness. For the same reason, odd roots of the troponin rate were examined, and the 5th root was selected. Log troponin measurement features and limit of detection indicators were multiplied by 10 to reduce the magnitude of the coefficients obtained in logistic regression. One troponin measurement was randomly selected in case more than one troponin measurement was available at follow up.

Table 2) and non-troponin variables, termed patient-specific features were considered during model development (Table 3). Six different high-sensitivity troponin assays were available for model development (Table 4). Non-high-sensitivity troponin assays did not qualify for model development.

Troponin measurements were log-transformed using the natural logarithm to reduce skewness. For the same reason, odd roots of the troponin rate were examined, and the 5th root was selected. Log troponin measurement features and limit of detection indicators were multiplied by 10 to reduce the magnitude of the coefficients obtained in logistic regression. One troponin measurement was randomly selected in case more than one troponin measurement was available at follow up.

Table 2: Troponin-based candidate features

Troponin concentration at baseline
Troponin concentration at baseline ≥ than lower limit of quantification
Troponin concentration at second measurement time point (2nd troponin)
2nd troponin concentration ≥ lower limit of quantification
Troponin rate (change over time)
Time between blood samples for troponin measurements

Table 3: Non-troponin candidate features

Variable	Coding	Remarks
Age	years	
Sex	male/female	
Heart failure	yes/no	
History of coronary artery disease	yes/no	
Family history of coronary artery disease	yes/no	Father/Brother with MI ≤ 55 years, Mother/Sister with MI ≤ 65 years
Atrial fibrillation	yes/no	
Systolic blood pressure	mm Hg	
Heart rate	bpm	
Hypertension	yes/no	
Hyperlipoproteinemia	yes/no	
Diabetes	yes/no	
Ever smoker	yes/no	
Body mass index (BMI)	(kg/m²)	
Electrocardiogram (ECG) ischemic signs	yes/no	The ECG was determined to be ischemic, when ischemic signs, ventricular arrhythmias (VT or VF), high-grade (>1) atrio-ventricular block, right, left, or bifascicular bundle branch block were observed. Ischemic signs included: any ST-depression (≥0.05 mV) or T-wave inversion (≥0.1mV) in two contiguous leads, or ST-elevation (≥0.1 mV or ≥0.2 mV in V2/3)
Symptom onset time ≥ than 3 hours	yes/no	

Table 4: Troponin assays used in this project

**High-sensitivity troponin**	**Manufacturer**	**Product name**
I	Abbott	Architect
I	Beckman-Coulter	Access
I	Mitsubishi	Pathfast
I	Siemens	Atellica
I	Siemens	Atellica VTLi
T	Roche	Elecsys

4. Model development (derivation)

A five-step approach was taken to for model development using the BACC study:

1. **Imputation of missing data:** Missing data in BACC were imputed using multiple imputation with *mice* (19). Five imputed datasets were generated; one randomly selected imputed dataset was kept for analyses. Only those subjects were kept for troponin assay-specific analyses if at least one assay measurement was available for this subject.
2. **Full machines:** Different learning machines were computed using all candidate features as input. The machine learning methods used in this project are described in Table 5. The machines lr, lrbs, lrus, glmboost, lrrcs, en, mars, gbm, rf, rffs, and svm were computed using the candidate features defined above. Support vector machines (SVM) were only used for methodological comparisons.

   Performance measures The following three measures were calculated to determine the performance of machines and super learners:

   - LogLoss (primary measure): $LogLoss = -\frac{1}{n}\sum_{i=1}^{n}(y_i \ln(p_i) + (1 - y_i)\ln(1 - p_i))$. Its variance is given by $Var(LogLoss) = \frac{1}{n^2}\sum_{i=1}^{n}\left(\ln\left(1 - p_i p_i(1 - p_i)\ln\frac{p_i}{(1 - p_i)}\right)\right)$.

   - Brier score (secondary measure; 20): $BS = \mathbb{E}(\mathrm{y_i} - \mathbb{P}(y_i = 1|\boldsymbol{x}_\mathrm{i}))^2$. The variance expression has been provided by Bradley, Schwartz (20).

   - AUC (secondary measure): the definition of the AUC has been provided, e.g., by Wang and Guo (21). We stress that the AUC measures classification performance. The variance expression has been provided by Wang and Guo (21). R code for performing the variance calculations are available in Appendix C of Wang and Guo (21). The code is displayed in Section 9 of this document.

   Cross-validation procedure Estimation was done using 10-fold cross-validation (10CV). Specifically, the dataset was randomly divided into v = 10 equally sized parts, the so-called folds. Each fold was removed from the dataset in turn; all machines were fitted using the remaining 9 folds, and its performance was evaluated in the left-out fold. At the end of this procedure, 10 estimates of the machines and their performance measures were obtained. The average over the 10 estimates was taken as performance measure. Machines were only compared for the same troponin-assay, and the same 10 folds were used to allow fair comparison of the machines.

   When a machine was fitted on the selected 9 folds, all the computations required to obtain the machine were performed inside the 9 folds. In particular, if there were parameters that required tuning, this was done inside of the 9 folds, possibly with a new CV inside the 9 folds.

3. **Feature selection:** The number of features was reduced using the CV procedure described above. Six of the full machines were used for the feature selection: lrbs, lrus, glmboost, en, mars and rffs. Since the same 10 CV folds were utilized in all steps of model development, the CV procedure was also used for feature selection. The following criteria were used to select features that were subsequently used in the reduced machines:
   - If a feature was selected ≥ 5 times out of the 10 CV folds for ≥ 1 assay model, the feature was kept.
   - Identical features were selected across assays. This means that if a feature was selected for a specific assay, it was automatically kept for the other assays.
   - Age was kept.
   - Sex was kept.
   - If a troponin measurement was selected, the corresponding limit of quantification indicator for that measurement was kepty, and vice versa.
4. **Reduced learning machines:** Learning machines were recomputed using the selected features only. Analyses were restricted to machines without intrinsic feature selection. lrbs, lrus and rffs were thus not computed on the reduced feature set.
5. **Super learner:** The four learning machines with the best LogLoss performance on the reduced feature set across all assays were used to develop super learners (SL) with equal weights (Slew).
   For a single hs-cTn measurement model, scatterplots showed that cross-validated MI probabilities of en, glmboost and lr were almost identical. For this reason, only glmboost was selected because it performed slightly better than en and lr.
   An optimized SL was obtained weighting the machines using a convex combination of using a linear lasso with non-negative normalized coefficients. The LogLoss and 10CV was used to select the lasso penalization parameter.
   Slews combining the best two, three, and four reduced machines were developed, and their performance was evaluated using the LogLoss. Its performance was compared with the other reduced machines and the optimized SL. The SL with the best performance across troponin assays was selected as the machine for estimating the probability of AMI.
6. **Comparison of learning machines:** To demonstrate the adequacy of the approach taken, the performance of the Slew in the reduced model was compared to
   a) the single machines,
   b) the Slew of the full models,
   c) the Slew of the models including troponin only,
   d) the Slew of full models also including eGFR,
   e) the Slew of reduced models also including eGFR.
   For these comparisons across troponin assays, we used the method described by Demšar (22). In brief, we first used the Iman and Davenport modification of the Friedman test (23) as global test. This was followed by the Nemenyi post-hoc test (24).

Table 5: Machine learning approaches used in the project

Machine	R function (R package)	Description
lr	*glm* (*stats*)	Logistic regression with all features entering the model in a linear form
lrbs	*fastbw* (*rms*)	Logistic regression with backward elimination. Candidate features were modeled linearly, and backward elimination was performed. This procedure starts with a model including all candidate predictors and drops the least significant at the level α. This is repeated until no non-significant terms remain. The significance level α was set to 0.05.
lrus	*glm* (*stats*)	Logistic regression with univariate screening. Logistic regressions using each candidate feature as the only predictor were computed. The final logistic regression includes those variables with p-value less than 0.05 in the univariate models.
glmboost	*glmboost* (*mboost)*	Component-wise gradient boosting using univariate logistic regressions as the base learner **(25)**. All features were centered. The number of boosting iterations was tuned using 10-fold cross-validation. The maximum number of boosting iterations was set to 10000. The model is described in detail below.
lrrcs	*lrm, rcs* (*rms*)	Logistic regression with the continuous predictors modeled in a flexible manner using restricted cubic splines. Five knots placed at the 0.05, 0.275, 0.5, 0.725 and 0.95 quantiles of each continuous variable were used.
en	*glmnet* (*glmnet*); *tidymodels* for tuning	Elastic net logistic regression. The elastic net is a mixture of the ridge (L_2 penalization) and the lasso (L_1 penalization) **(26)**. As these two the elastic net shrinks the coefficients towards zero and like the lasso it performs feature selection. The elastic net mixing parameter and the penalization parameter were estimated by 10-fold cross-validation (10CV).

mars	*earth* (*earth*); *tidymodels* for tuning	Multivariate adaptive regression splines **(27)**. 10CV was used to select the following parameters: maximum degree of interactions and maximum number of terms in the model.
gbm	*xgboost* (*xgboost*); tidymodels for tuning	Gradient boosting machine with trees as the base learners. The following hyperparameters were estimated by minimization of the LogLoss using 10CV: depth of the tree, number of trees, learn rate, reduction in the loss function required to split further, proportion of candidate variables sampled at each split, proportion of observations used to fit each tree and minimum number of observations in a terminal node. The model is described in detail below.
rf	*ranger* (*ranger*)	Random forest (RF) in regression mode with subsampling and maximally selected rank statistics as split rule **(28)**. The number of variables to consider for splitting at each node (*mtry*), minimal terminal node size (*nodesize*) and the sample fraction *sample.fraction* were tuned. The *ranger* function from the *ranger* package was used **(29)**. The model is described in detail below.
rffs	*ranger, importance_pvalues* (*ranger*)	RF with feature selection in regression mode with subsampling and maximally selected rank statistics as split rule **(28).** First parameters mtry, nodesize and *sample.fraction* tuned. Second, permutation p-values were computed using 1000 permutations **(30)**. Features were kept if $p < 0.05$. Third, a new RF was computed tuning the same parameters as before.
svm	*ksvm* (*kernlab*); tidymodels for tuning	Support vector machines with radial basis function kernel. Minimization of the LogLoss using 10CV was used to select the parameters cost of constraints violations and the inverse kernel width for the radial basis function
sl		Super learner (SL). The SL merges different machines by assessing their performance and then generating an optimal convex combination of the predicted probabilities from the different machines

	**(31)**. Ten-fold cross-validation and the LogLoss were used to select the optimal combination.
slew	Super learner with equal weights. This simpler version of the SL uses a convex combination with identical weights.

5. Validation of model

The assay-specific models were applied in stenoCardia for validation. Multiple imputation was performed as in the BACC data. Probability estimates were recalibrated using the four different approaches described in the next Section 6.

6. Calibration

Four calibration approaches were employed in both the validation and the generalization step: no calibration, logistic calibration, logistic calibration with restricted cubic splines and Elkan's approach to recalibration.

The performance was evaluated using the measures described in Section 4, item 2.

6.1 Probability estimation – uncalibrated

Probability estimates were obtained for the validation data and for each of the *m* imputed datasets from generalization. Probability estimates were calculated for the three machines single machines included in the Slew and the Slew for the single hs-cTn measurement model, and all four machines included in the Slew and the Slew for the serial hs-cTn measurement model.

6.2 Probability estimation – calibrated

6.2.1 Logistic regression with restricted cubic splines (RCS) (logreg-RCS)

To calibrate probability estimates, we used logistic regression with restricted cubic splines (RCS) with k = 4 knots (32). In case of numerical instabilities, we reduced the number the knots. The location of the knots were chosen using the quantiles of the data (32).

6.2.2 Logistic regression (logreg)

For sake of comparison with logreg-RCS we also used logistic regression calibration, i.e., a logistic regression was fitted to the outcome using the linear predictor from the probability of NSTEMI as explanatory variable.

6.2.3 Elkan's approach

The calibrated probability can be estimated using the prevalence of MI in the old and new populations using the update formula (33)

$$p' = P(y = 1|\boldsymbol{x}) = b' \frac{p(1-b)}{b(1-b) + b'(p-b)},$$

where b (b') is the prevalence in the old (new) population, and p (p') is the probability estimate in the old (new) population.

7. Generalization of model

The assay-specific models for the single and serial hs-cTn measurement models were applied to the international cohort studies described in Section 2.3. Calibration analyses were performed as described for stenoCardia.

7.1 Multiple imputation

For the imputation, the following MI strategy was adopted in conjunction with Rubin's rule (RR). The imputation strategy for each cohort of interest depended on whether all variables of interest were present and contained data in the dataset (Section 7.1.1), or if one or more variable of interest is missing (Section 7.1.2).

The imputation was implemented using the `mice` package, and perfect mean matching (PMM) with *m* = 100 imputations and *iter* = 15 iterations. *iter* = 50 was selected with *m* = 5 to visualize imputation quality. The variables from the reduced model were included in the multiple imputation, but adjudicated outcomes or incident cardiovascular events were not imputed.

Following the imputation of the cohorts, the imputed datasets were extracted individually or as a stacked dataset using the complete function.

The RR MI pools effect estimates across *m* imputed datasets is $\bar{\theta} = \frac{1}{m}\sum_{d=1}^{m}\theta_d$. Additionally, the pooled standard error was calculated using the within and between imputation variances. The within imputation variance, which reflects the sampling variance, is calculated as $Var_W = \frac{1}{m}\sum_{d=1}^{m}SE_d^2$, where SE_d^2 is the squared standard error (SE) estimated in each imputed dataset d.

The between imputation variance, which reflects extra variance due to the missing data, is calculated as

$$Var_B = \frac{\sum_{d=1}^{m}(\theta_d - \bar{\theta})^2}{m-1}.$$

The total variance is then given by

$$Var_{Total} = Var_W + Var_B + \frac{Var_B}{m}.$$

7.1.1 Imputation when all variables were present

When all variables were present within the cohort of interest, cohort-specific imputation of missing data was performed using data only from the cohort of interest.

7.1.2 Imputation in the presence of missing variables

When at least one variable was completely missing within the cohort of interest, global imputation was applied using the original data from all cohorts, in which the troponin assay of interest was measured.

7.2 Performance measures

For each cohort of interest, the LogLoss, Brier score and AUC were calculated. The point estimate and variance of the LogLoss, Brier score and AUC were pooled across the m imputed datasets using RR.

7.3 Classification performance

The classification performance was quantified by pooling the sensitivity, specificity, as well as the positive and negative predictive values. To quantify the variance of these parameters across the imputed datasets, the binomial variance estimator $\text{Var}(\hat{p}) = \frac{p(1-p)}{n}$ was used.

7.4 Meta-analysis: pooling of estimates across studies

Estimates were pooled across cohorts using random effect (RE) meta-analysis according to DerSimonian and Laird (4).

7.5 Plots

For visualization, local estimated scatterplot smoothing (LOESS) curves representing the MI and the calibrated predicted probability were created.

7.6 Incorporation of the Siemens Atellica VTLi

While the other five investigational hs-cTn assays were all available at time of model development, the Atellica VTLi was added to the super learners post-hoc by estimating a total least squares regression from the Atellica VTLi hs-cTnI to the Atellica hs-cTnI using 135 subjects from BACC with two troponin measurements available each on both assays. Total least squares (TLS) regression, also termed Deming regression, with intercept and slope was estimated to predict Siemens Atellica troponin concentrations from Siemens Atellica VTLi troponin concentrations. The single and serial hs-cTn measurement models were applied after predicting the Atellica troponin concentrations from the Atellica VTLi hs-cTn concentrations.

8. Sample size considerations

Sample size considerations were done for the derivation, validation, and generalization cohorts as well as the sum over all generalization cohorts using the approach of Riley et al.(34) To this end, sample sizes were fixed as well as the proportion of myocardial infarctions. The precision for the MI probability was estimated. In the derivation cohort, the precision was well below 0.015, for the validation it was 0.0170. Finally, each cohort from the generalization studies, the precision was between 0.01 and 0.023.

Table 6: Sample size estimation

Stage of study	Name of cohort	Sample size	Proportion of MIs	Precision for outcome proportion
**Derivation**	BACC	2,575	0.14	0.0135

**Validation**	stenoCardia	1,677	0.17	0.0180
**Generalization**	ADAPT-BSN	2,315	0.11	0.0155
	ADPs-CH	3,119	0.11	0.0132
	DROP-ACS	1,420	0.11	0.0200
	FASTEST	1,233	0.11	0.0195
	High-STEACS	1,750	0.11	0.0177
	LUND	1,164	0.11	0.0217
	Rapid-CPU	4,938	0.11	0.0105
	ROMI	1,366	0.11	0.0200
	SAMIE	1,985	0.11	0.0165
	SEIGE and SAFETY	1,033	0.11	0.0230
	STOP-CP	1,457	0.11	0.0194
	UTROPIA	1,631	0.11	0.0183
	Total	23,411	0.11	0.0050

9. Calculation of the variance of the AUC

The code displayed in this section follows the code provided by Wang and Guo (21).

```
#mydata: a data frame with a column called “Y” for the binary outcome variable
#myfit: a glm object (it can be changed to other types of model object)
#B: the number of partitions used to realize the variance estimator (see Model
1)
V_AUC_partition <- function(mydata, myfit, B)
{
#data subsets for positive and negative classes
pos_class <- subset(mydata,Y==1)
neg_class <- subset(mydata,Y==0)
m <- min(nrow(pos_class), nrow(neg_class))
phi_bar_b <-array(NA, B)
WPSS <- rep(NA,B)
for (b in 1:B)
{
#for each sampled partition
kernel <- array(NA, m)
pos_shuf <- sample(which(mydata$Y==1), m, replace=F)
neg_{s}huf <- sample(which(mydata$Y==0), m, replace=F)
prob <- predict.glm(myfit, type=“response”)
for(pair in 1:m)
{
#for each block of paired data based on the partition
kernel[pair] <- as.numeric(prob[pos_shuf[pair]] > prob[neg_shuf[pair]])
}
```

```
phi_bar_b[b] <- mean(kernel)
#compute the within-partition sum of squares

WPSS[b] <- (1/(m-1) )* sum ((kernel - ph_bar_b[b])^2)
}
phi_bar <- mean(phi_bar_b)
#compute the between-partition sum of squares
BPSS <- mean((phi_bar_b-phi_bar)^2)
V_AUC_part <- (1/m)*mean(WPSS) - BPSS
return(V_AUC_part)
}
```

10. References

1. Azur MJ, Stuart EA, Frangakis C, Leaf PJ. Multiple imputation by chained equations: what is it and how does it work? Int J Methods Psychiatr Res. 2011;20(1):40-9.
2. Levey AS, Stevens LA, Schmid CH, Zhang YL, Castro AF, 3rd, Feldman HI, et al. A new equation to estimate glomerular filtration rate. Ann Intern Med. 2009;150(9):604-12.
3. Demšar J. Statistical Comparisons of Classifiers over Multiple Data Sets. J Mach Learn Res. 2006;7:1–30.
4. DerSimonian R, Laird N. Meta-analysis in clinical trials revisited. Contemporary Clinical Trials. 2015;45:139-45.
5. R-Core-Team. R: A language and environment for statistical computing. Foundation for Statistical Computing, Vienna, Austria2013.
6. Neumann JT, Sörensen NA, Schwemer T, Ojeda F, Bourry R, Sciacca V, et al. Diagnosis of myocardial infarction using a high-sensitivity troponin I 1-hour algorithm. JAMA Cardiol. 2016;1(4):397-404.
7. Keller T, Zeller T, Peetz D, Tzikas S, Roth A, Czyz E, et al. Sensitive troponin I assay in early diagnosis of acute myocardial infarction. New England Journal of Medicine. 2009;361(9):868-77.
8. Than M, Cullen L, Aldous S, Parsonage WA, Reid CM, Greenslade J, et al. 2-Hour accelerated diagnostic protocol to assess patients with chest pain symptoms using contemporary troponins as the only biomarker: the ADAPT trial. J Am Coll Cardiol. 2012;59:2091-8.
9. Than M, Aldous S, Lord SJ, Goodacre S, Frampton CM, Troughton R, et al. A 2-hour diagnostic protocol for possible cardiac chest pain in the emergency department: a randomized clinical trial. JAMA Intern Med. 2014;174(1):51-8.
10. Than MP, Pickering JW, Aldous SJ, Cullen L, Frampton CM, Peacock WF, et al. Effectiveness of EDACS Versus ADAPT Accelerated Diagnostic Pathways for Chest Pain: A Pragmatic Randomized Controlled Trial Embedded Within Practice. Annals of emergency medicine. 2016;68(1):93-102 e1.
11. Pickering JW, Young JM, George PM, Pemberton CJ, Watson A, Aldous SJ, et al. Early kinetic profiles of troponin I and T measured by high-sensitivity assays in patients with myocardial infarction. Clin Chim Acta. 2020;505:15-25.
12. Shiozaki M, Inoue K, Suwa S, Lee CC, Chikata Y, Ishiura J, et al. Utility of the 0-hour/1-hour high-sensitivity cardiac troponin T algorithm in Asian patients with suspected non-ST elevation myocardial infarction. Int J Cardiol. 2017;249:32-5.
13. Chapman AR, Anand A, Boeddinghaus J, Ferry AV, Sandeman D, Adamson PD, et al. Comparison of the Efficacy and Safety of Early Rule-Out Pathways for Acute Myocardial Infarction. Circulation. 2017;135(17):1586-96.
14. Mokhtari A, Borna C, Gilje P, Tydén P, Lindahl B, Nilsson H-J, et al. A 1-h Combination Algorithm Allows Fast Rule-Out and Rule-In of Major Adverse Cardiac Events. J Am Coll Cardiol. 2016;67(13):1531-40.
15. Stoyanov KM, Biener M, Hund H, Mueller-Hennessen M, Vafaie M, Katus HA, et al. Effects of crowding in the emergency department on the diagnosis and management of suspected acute coronary syndrome using rapid algorithms: an observational study. BMJ open. 2020;10(10):e041757.

16. Shortt C, Ma J, Clayton N, Sherbino J, Whitlock R, Pare G, et al. Rule-In and Rule-Out of Myocardial Infarction Using Cardiac Troponin and Glycemic Biomarkers in Patients with Symptoms Suggestive of Acute Coronary Syndrome. Clin Chem. 2017;63(1):403-14.
17. Allen BR, Christenson RH, Cohen SA, Nowak R, Wilkerson RG, Mumma B, et al. Diagnostic Performance of High-Sensitivity Cardiac Troponin T Strategies and Clinical Variables in a Multisite US Cohort. Circulation. 2021;143(17):1659-72.
18. Sandoval Y, Smith SW, Shah AS, Anand A, Chapman AR, Love SA, et al. Rapid Rule-Out of Acute Myocardial Injury Using a Single High-Sensitivity Cardiac Troponin I Measurement. Clin Chem. 2016.
19. van Buuren S, Groothuis-Oudshoorn K. mice: multivariate imputation by chained qquations in R. Journal of Statistical Software. 2011;45(3):1-67.
20. Bradley AA, Schwartz SS, Hashino T. Sampling uncertainty and confidence intervals for the Brier score and Brier skill score. Weather and Forecasting. 2008;23(5):992-1006.
21. Wang Q, Guo A. An efficient variance estimator of AUC and its applications to binary classification. Statistics in Medicine. 2020;39(28):4281-300.
22. Demšar J. Statistical comparisons of classifiers over multiple data sets. Journal of Machine Learning Research. 2006;7(1):1-30.
23. Iman RL, Davenport JM. Approximations of the critical region of the Friedman statistic. Commun Stat Theor Meth. 1980;9(6):571-95.
24. Nemenyi PB. Distribution-free multiple comparisons. Princeton: Princeton University; 1963.
25. Hofner B, Mayr A, Robinzonov N, Schmid M. Model-based boosting in R: a hands-on tutorial using the R package mboost. Computational Statistics. 2014;29(1):3-35.
26. Zou H, Hastie T. Regularization and variable selection via the elastic net. Journal of the Royal Statistical Society, Series B. 2005;67(2):301-20.
27. Friedman JH. Multivariate adaptive regression splines. Annals of Statistics. 1991;19(1):1-67.
28. Wright MN, Dankowski T, Ziegler A. Unbiased split variable selection for random survival forests using maximally selected rank statistics. Statistics in Medicine. 2017;36(8):1272-84.
29. Wright MN, Ziegler A. ranger: a fast implementation of random forests for high dimensional data in C++ and R. Journal of Statistical Software. 2017;77(1):1.
30. Altmann A, Tolosi L, Sander O, Lengauer T. Permutation importance: a corrected feature importance measure. Bioinformatics. 2010;26(10):1340-7.
31. van der Laan MJ, Polley EC, Hubbard AE. Super learner. Statistical Applications in Genetics and Molecular Biology. 2007;6:Article25.
32. Harrell FE, Jr. Regression Modeling Strategies. With Applications to Linear Models, Logistic and Ordinal Regression, and Survival Analysis. 2nd ed ed. Cham: Springer International Publishing; 2015.
33. Elkan C. The foundations of cost-sensitive learning. IJCAI'01 Proceedings of the 17th international joint conference on Artificial intelligence. 2. San Francisco: Morgan Kaufmann Publishers Inc; 2001. p. 973-8.
34. Riley RD, Snell KI, Ensor J, Burke DL, Harrell FE, Jr., Moons KG, et al. Minimum sample size for developing a multivariable prediction model: PART II - binary and time-to-event outcomes. Stat Med. 2019;38(7):1276-96.
