# Supplementary material for: Personalized diagnosis in suspected myocardial infarction
Source: Clin Res Cardiol. 2023 May 2;112(9):1288–301. doi: 10.1007/s00392-023-02206-3 (PMC10449973; doi:10.1007/s00392-023-02206-3)
Supplement: Supplementary file 5 — Supplementary file5: Supplementary Figures an tables (PDF 3514 KB) [file 392_2023_2206_MOESM5_ESM.pdf]

Personalized diagnosis in suspected myocardial infarction

Supplementary Material:

Supplemental Figures and Tables

April 26, 2023

Version 4.5

1. SUPPLEMENTAL FIGURES.....4

FIGURE S1: FLOW-CHART OF STUDY DESIGN 4
FIGURE S2: PERFORMANCE COMPARISON OF THE SUPER LEARNER FROM THE REDUCED MODEL (REDUCED) AGAINST A MODEL USING TROPONIN ONLY (TROPONIN), AND THE FULL MODEL (FULL) AVERAGED ACROSS ALL HS-CTN ASSAYS IN THE DERIVATION COHORT (UPPER PANEL) AND THE VALIDATION COHORT (LOWER PANEL). 5
FIGURE S3: PERFORMANCE COMPARISON OF THE SUPER LEARNER FROM THE REDUCED MODEL (SLEW) AGAINST SINGLE LEARNING MACHINES (LR, LRRCS, EN, GLMBOOST, MARS, GBM, SVM, RF) AVERAGED ACROSS ALL HS-CTN ASSAYS IN THE DERIVATION COHORT (UPPER PANEL) AND THE VALIDATION COHORT (LOWER PANEL)..... 6
FIGURE S4: PERFORMANCE COMPARISON OF THE SUPER LEARNER FROM THE REDUCED MODEL (REDUCED) AGAINST THE FULL MODEL AND THE REDUCED MODEL INCLUDING EGFR (FULL EGFR AND REDUCED EGFR, RESPECTIVELY) AVERAGED ACROSS ALL HS-CTN ASSAYS IN THE DERIVATION COHORT (UPPER PANEL) AND THE VALIDATION COHORT (LOWER PANEL). 7
FIGURE S5: DISCRIMINATION MEASURES USING THE SUPER-LEARNER BASED ON A SINGLE AND ON A SERIAL HS-CTN MEASUREMENT PER ASSAY 8
FIGURE S7: CALIBRATION PLOT FOR SINGLE AND SERIAL HS-CTN MEASUREMENT PER ASSAY AND THE GENERALIZATION DATASETS 12
FIGURE S8: DIAGNOSTIC ACCURACY USING THE MYOCARDIAL INFARCTION PROBABILITY AS THRESHOLD 14
FIGURE S9: SCATTERPLOT AND DIAGNOSTIC PERFORMANCE USING ATELLICA VTLI 15

2. SUPPLEMENTAL TABLES 17

TABLE S1: TRIPOD CHECKLIST..... 17
TABLE S2: BASELINE CHARACTERISTICS FOR DERIVATION, VALIDATION AND GENERALIZATION COHORTS INCLUDING INFORMATION ON MISSINGNESS 19
TABLE S3: BASELINE CHARACTERISTICS FOR ALL GENERALIZATION COHORTS 20
TABLE S4: BASELINE CHARACTERISTICS FOR ALL GENERALIZATION COHORTS STRATIFIED BY MI DIAGNOSIS 22
TABLE S5: DETAILED BASELINE CHARACTERISTICS FOR ALL COHORTS OF PARAMETERS USED IN THE MODELS 25
TABLE S6: VARIABLES AND MACHINES SELECTED FOR THE SINGLE AND THE SERIAL HS-CTN MEASUREMENT MODELS 29
TABLE S7: DISCRIMINATION MEASURES (LOGLOSS, AUC, BS) FOR SINGLE AND SERIAL HS-CTN MEASUREMENT FOR EACH COHORT AND THE GLOBAL DATASET PER ASSAY, UNCALIBRATED AND CALIBRATED USING LOGISTIC REGRESSION, CALIBRATION USING LOGISTIC REGRESSION WITH RESTRICTED CUBIC SPLINES AND BY ELKAN CALIBRATION. 30
TABLE S8: DIAGNOSTIC PERFORMANCE MEASURES FOR USING DIFFERENT MI PROBABILITIES AS THRESHOLDS FOR THE SINGLE HS-CTN MEASUREMENT MODEL 31

TABLE S9: DIAGNOSTIC PERFORMANCE MEASURES FOR USING DIFFERENT MI PROBABILITIES AS THRESHOLDS FOR THE SERIAL HS-CTN MEASUREMENT MODEL 32
TABLE S10: COMPARISON OF ESC 0H, 0/1H AND 0/2H ALGORITHMS WITH THE ARTEMIS PATHWAY USING A SINGLE OR A SERIAL HS-CTN MEASUREMENT. 33

1. Supplemental Figures

Figure S1: Flow-chart of study design

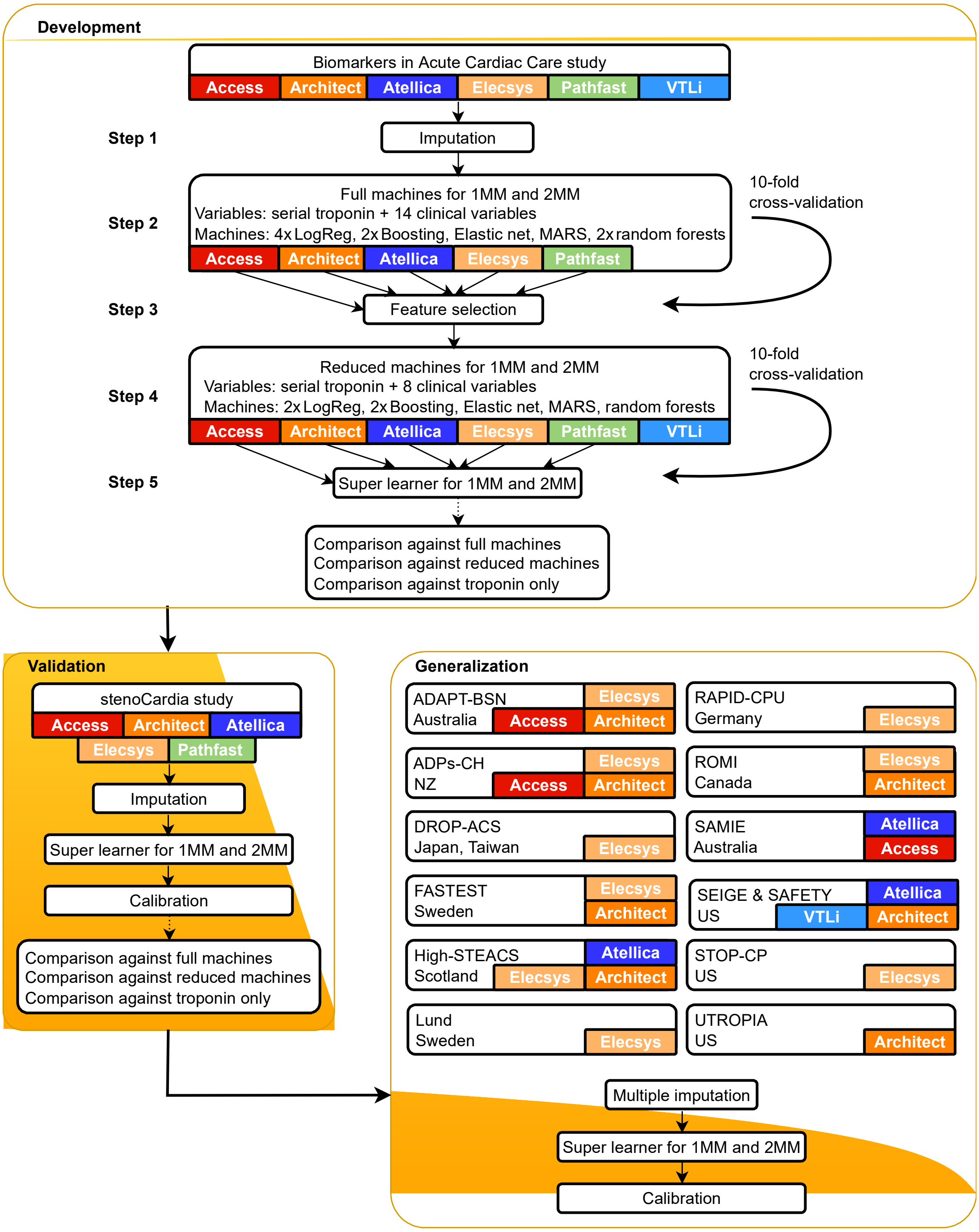

This figure illustrates the workflow of the entire study design. Abbreviations: 1 MM = single hs-cTn measurement, 2 MM = serial hs-cTn measurement

Figure S2: Performance comparison of the super learner from the reduced model (reduced) against a model using troponin only (troponin), and the full model (full) averaged across all hs-cTn assays in the derivation cohort (upper panel) and the validation cohort (lower panel).

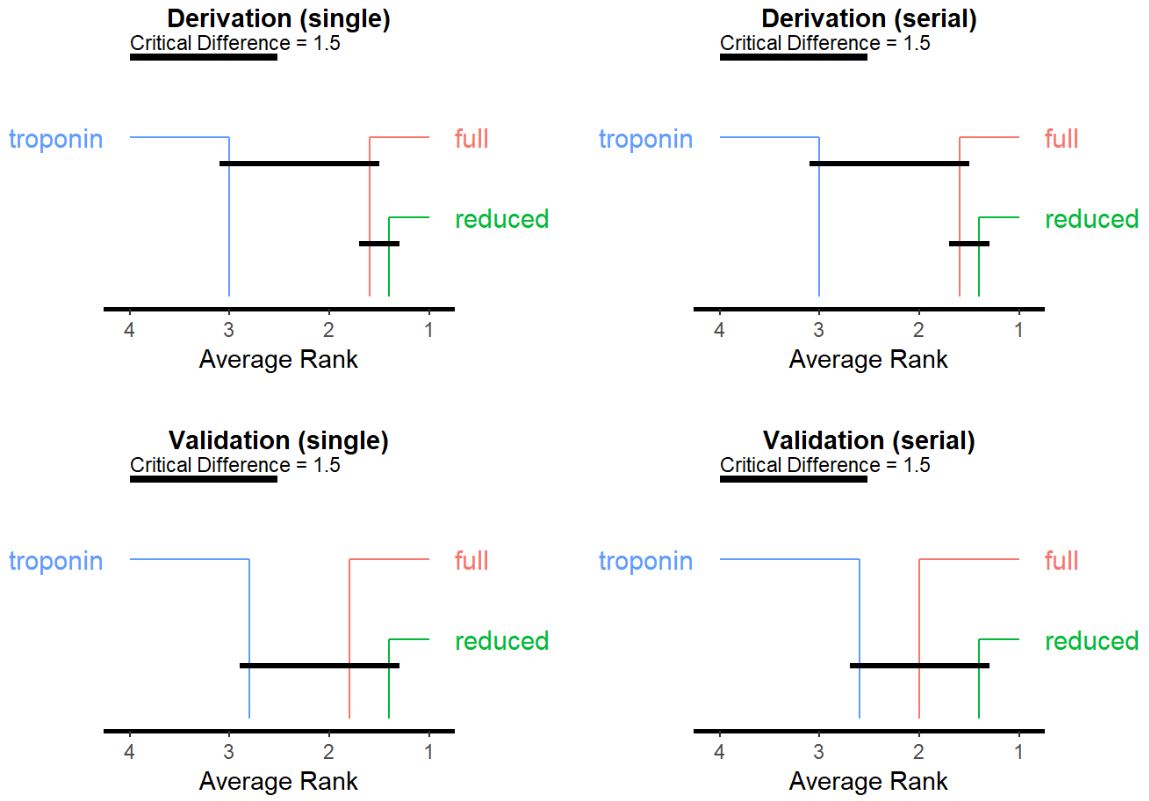

Comparison between models based on the LogLoss performance measure averaged over all troponin assays. In the validation cohort probability estimates were calibrated using logistic regression with restricted cubic splines. The super learner from the reduced model had the lowest average rank, i.e., the best performance, followed by the super learner from the full model. The super learner based on the troponin only model always performed worst. If the average rank difference between models was larger than the critical difference 1.5, the performance was significantly different at the 5% test-level using the Nemenyi post-hoc test, which adjusts for multiple testing.

Figure S3: Performance comparison of the super learner from the reduced model (slew) against single learning machines (lr, lrrcs, en, glmboost, mars, gbm, svm, rf) averaged across all hs-cTn assays in the derivation cohort (upper panel) and the validation cohort (lower panel).

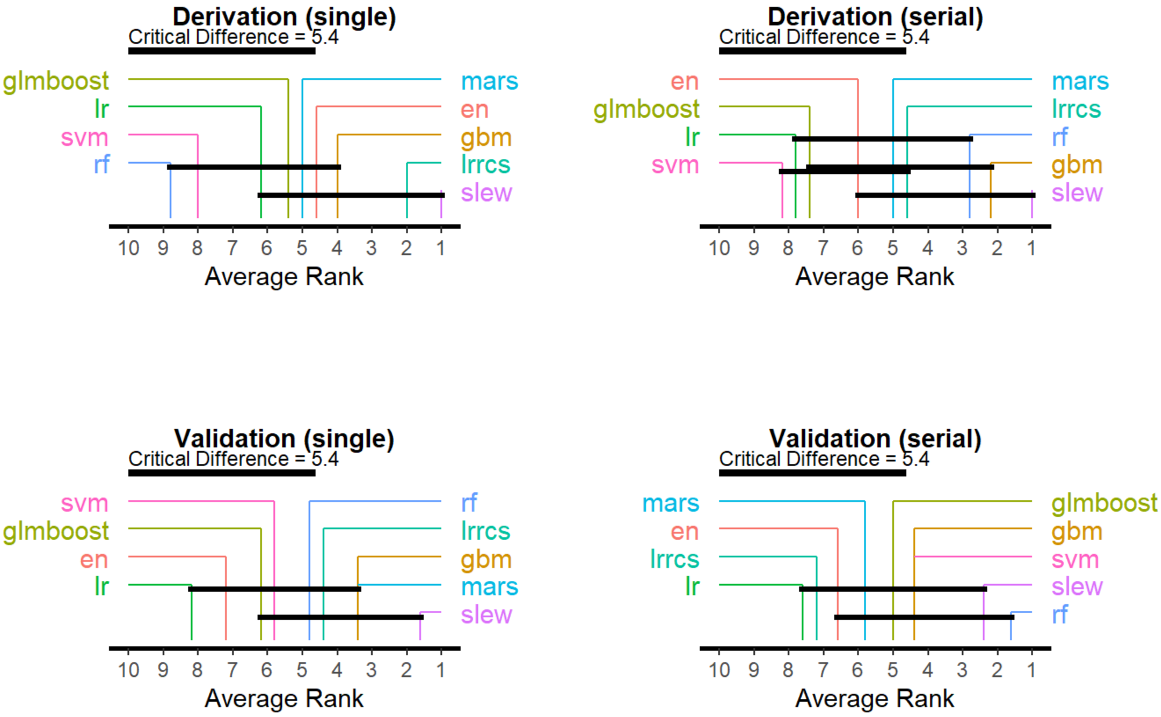

Comparison between models based on the LogLoss performance measure averaged over all troponin assays. In the validation cohort probability estimates were calibrated using logistic regression with restricted cubic splines. The super learner from the reduced model (slew) had the lowest average rank in three of four cases. The random forest (rf) performed best on the validation data for the model with serial measurements. If the average rank difference between models was larger than the critical difference 5.4, the performance was significantly different at the 5% test-level using the Nemenyi post-hoc test, which adjusts for multiple testing. svm: support vector machine; glmboost: logistic boosting, en: elastic net; lr: logistic regression; lrrcs: logistic regression with restricted cubic splines; gbm: gradient boosting; mars: multiple adaptive regression splines.

Figure S4: Performance comparison of the super learner from the reduced model (reduced) against the full model and the reduced model including eGFR (full egfr and reduced egfr, respectively) averaged across all hs-cTn assays in the derivation cohort (upper panel) and the validation cohort (lower panel).

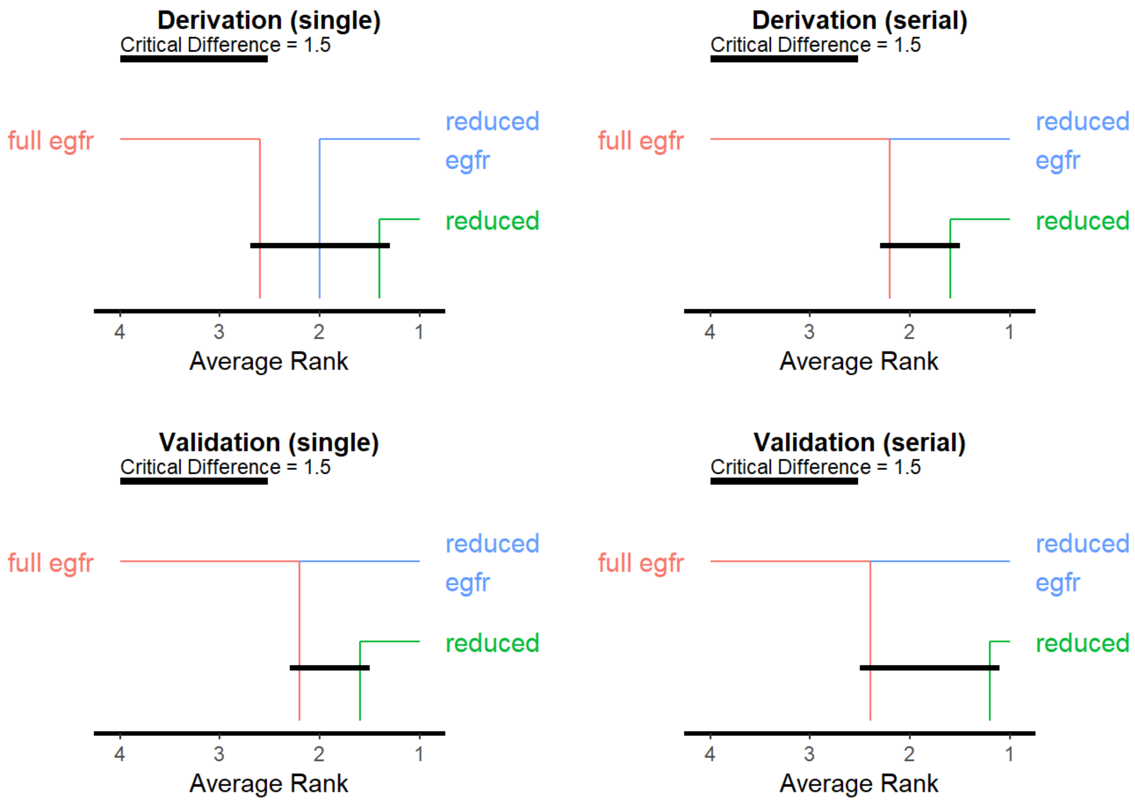

Comparison between models based on the LogLoss performance measure averaged over all troponin assays. In the validation cohort probability estimates were calibrated using logistic regression with restricted cubic splines. The super learner from the reduced model had the lowest average rank, i.e., the best performance, followed by the super learner from the reduced model that included eGFR. The super learner based on the full model including eGFR always performed worst. If the average rank difference between models was larger than the critical difference 1.5, the performance was significantly different at the 5% test-level using the Nemenyi post-hoc test, which adjusts for multiple testing.

Figure S5: Discrimination measures using the super-learner based on a single and on a serial hs-cTn measurement per assay

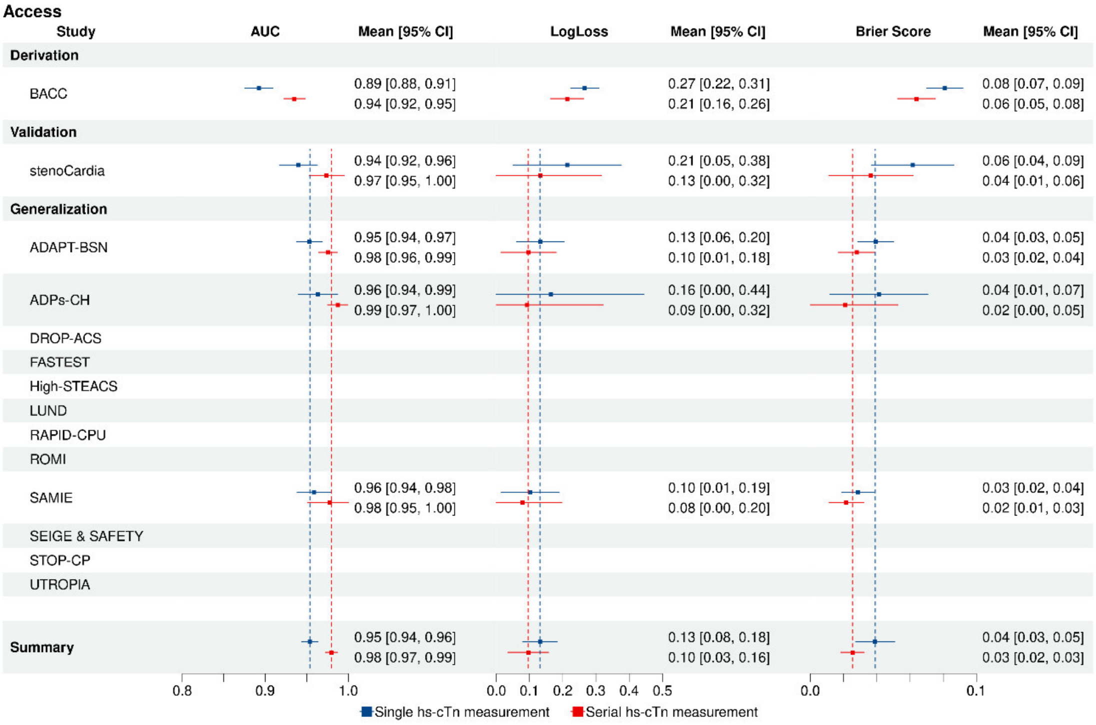

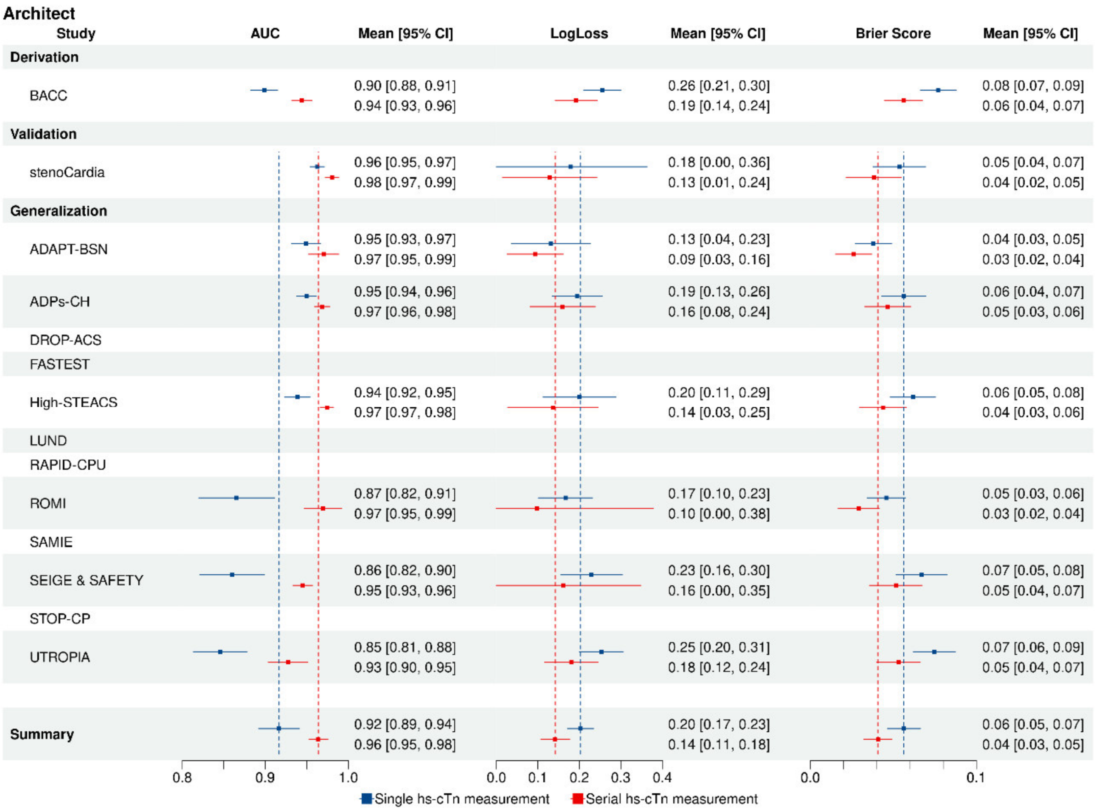

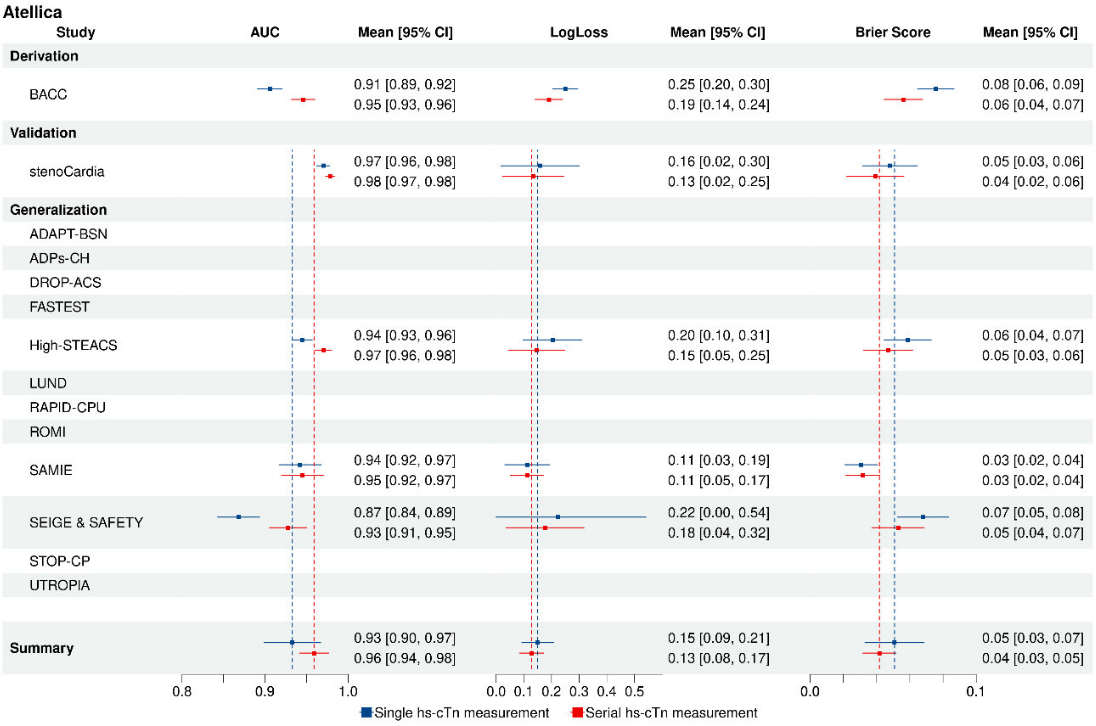
Atellica
Study
AUC
Mean [95% CI]
LogLoss
Mean [95% CI]
Brier Score
Mean [95% CI]
Derivation
BACC
0.91 [0.89, 0.92]
0.95 [0.93, 0.96]
0.25 [0.20, 0.30]
0.19 [0.14, 0.24]
0.08 [0.06, 0.09]
0.06 [0.04, 0.07]
Validation
stenoCardia
0.97 [0.96, 0.98]
0.98 [0.97, 0.98]
0.16 [0.02, 0.30]
0.13 [0.02, 0.25]
0.05 [0.03, 0.06]
0.04 [0.02, 0.06]
Generalization
ADAPT-BSN
ADPs-CH
DROP-ACS
FASTEST
High-STEACS
0.94 [0.93, 0.96]
0.97 [0.96, 0.98]
0.20 [0.10, 0.31]
0.15 [0.05, 0.25]
0.06 [0.04, 0.07]
0.05 [0.03, 0.06]
LUND
RAPID-CPU
ROMI
SAMIE
0.94 [0.92, 0.97]
0.95 [0.92, 0.97]
0.11 [0.03, 0.19]
0.11 [0.05, 0.17]
0.03 [0.02, 0.04]
0.03 [0.02, 0.04]
SEIGE & SAFETY
0.87 [0.84, 0.89]
0.93 [0.91, 0.95]
0.22 [0.00, 0.54]
0.18 [0.04, 0.32]
0.07 [0.05, 0.08]
0.05 [0.04, 0.07]
STOP-CP
UTROPIA
Summary
0.93 [0.90, 0.97]
0.96 [0.94, 0.98]
0.15 [0.09, 0.21]
0.13 [0.08, 0.17]
0.05 [0.03, 0.07]
0.04 [0.03, 0.05]
0.8
0.9
1.0
0.0
0.1
0.2
0.3
0.4
0.5
0.0
0.1
Single hs-cTn measurement
Serial hs-cTn measurement

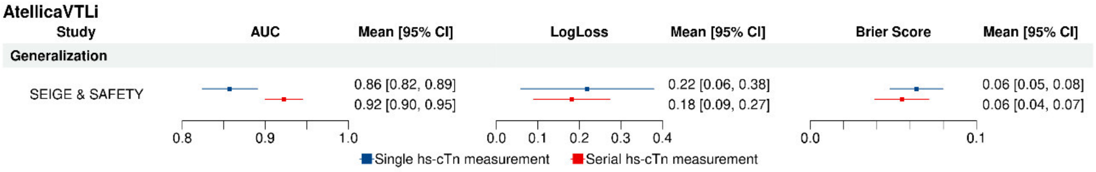
AtellicaVTLi
Study
AUC
Mean [95% CI]
LogLoss
Mean [95% CI]
Brier Score
Mean [95% CI]
Generalization
SEIGE & SAFETY
0.86 [0.82, 0.89]
0.92 [0.90, 0.95]
0.22 [0.06, 0.38]
0.18 [0.09, 0.27]
0.06 [0.05, 0.08]
0.06 [0.04, 0.07]
0.8
0.9
1.0
0.0
0.1
0.2
0.3
0.4
0.0
0.1
Single hs-cTn measurement
Serial hs-cTn measurement

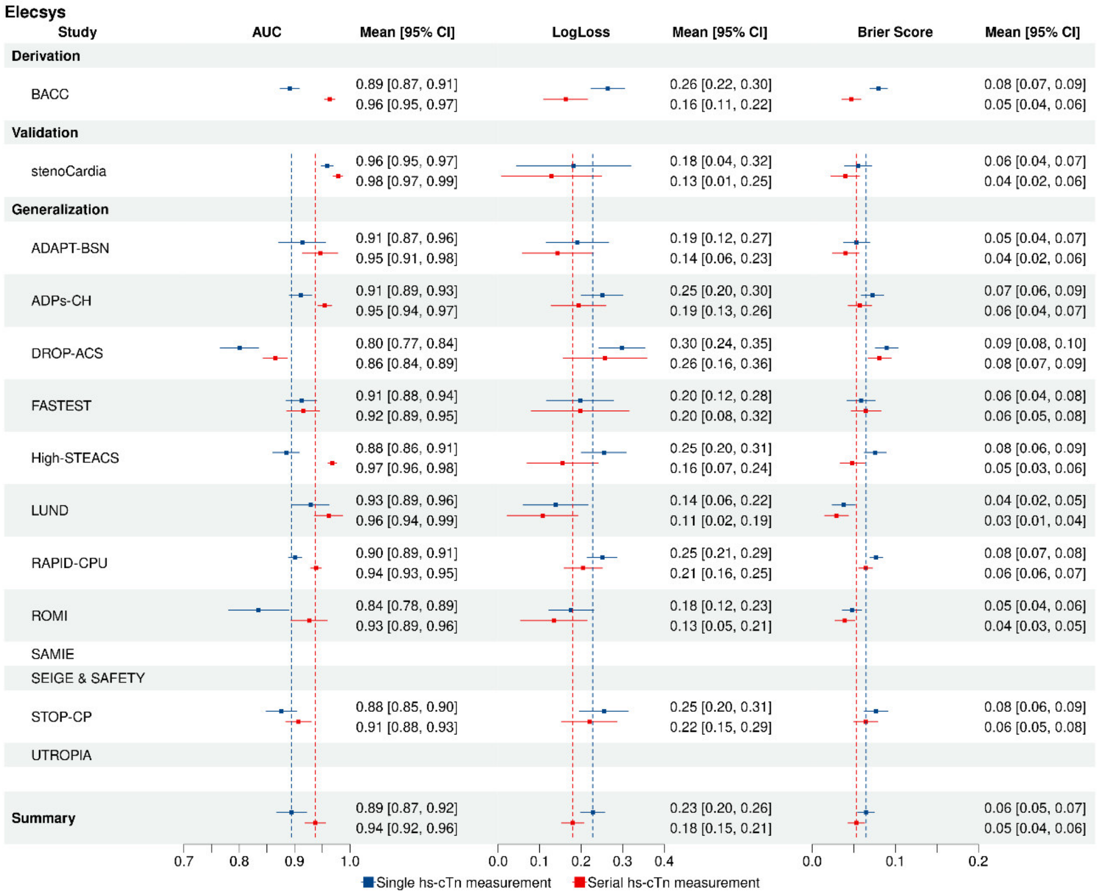
Elecsys
Study
AUC
Mean [95% CI]
LogLoss
Mean [95% CI]
Brier Score
Mean [95% CI]
Derivation
BACC
0.89 [0.87, 0.91]
0.96 [0.95, 0.97]
0.26 [0.22, 0.30]
0.16 [0.11, 0.22]
0.08 [0.07, 0.09]
0.05 [0.04, 0.06]
Validation
stenoCardia
0.96 [0.95, 0.97]
0.98 [0.97, 0.99]
0.18 [0.04, 0.32]
0.13 [0.01, 0.25]
0.06 [0.04, 0.07]
0.04 [0.02, 0.06]
Generalization
ADAPT-BSN
0.91 [0.87, 0.96]
0.95 [0.91, 0.98]
0.19 [0.12, 0.27]
0.14 [0.06, 0.23]
0.05 [0.04, 0.07]
0.04 [0.02, 0.06]
ADPs-CH
0.91 [0.89, 0.93]
0.95 [0.94, 0.97]
0.25 [0.20, 0.30]
0.19 [0.13, 0.26]
0.07 [0.06, 0.09]
0.06 [0.04, 0.07]
DROP-ACS
0.80 [0.77, 0.84]
0.86 [0.84, 0.89]
0.30 [0.24, 0.35]
0.26 [0.16, 0.36]
0.09 [0.08, 0.10]
0.08 [0.07, 0.09]
FASTEST
0.91 [0.88, 0.94]
0.92 [0.89, 0.95]
0.20 [0.12, 0.28]
0.20 [0.08, 0.32]
0.06 [0.04, 0.08]
0.06 [0.05, 0.08]
High-STEACS
0.88 [0.86, 0.91]
0.97 [0.96, 0.98]
0.25 [0.20, 0.31]
0.16 [0.07, 0.24]
0.08 [0.06, 0.09]
0.05 [0.03, 0.06]
LUND
0.93 [0.89, 0.96]
0.96 [0.94, 0.99]
0.14 [0.06, 0.22]
0.11 [0.02, 0.19]
0.04 [0.02, 0.05]
0.03 [0.01, 0.04]
RAPID-CPU
0.90 [0.89, 0.91]
0.94 [0.93, 0.95]
0.25 [0.21, 0.29]
0.21 [0.16, 0.25]
0.08 [0.07, 0.08]
0.06 [0.06, 0.07]
ROMI
0.84 [0.78, 0.89]
0.93 [0.89, 0.96]
0.18 [0.12, 0.23]
0.13 [0.05, 0.21]
0.05 [0.04, 0.06]
0.04 [0.03, 0.05]
SAMIE
SEIGE & SAFETY
STOP-CP
0.88 [0.85, 0.90]
0.91 [0.88, 0.93]
0.25 [0.20, 0.31]
0.22 [0.15, 0.29]
0.08 [0.06, 0.09]
0.06 [0.05, 0.08]
UTROPIA
Summary
0.89 [0.87, 0.92]
0.94 [0.92, 0.96]
0.23 [0.20, 0.26]
0.18 [0.15, 0.21]
0.06 [0.05, 0.07]
0.05 [0.04, 0.06]
0.7 0.8 0.9 1.0
0.0 0.1 0.2 0.3 0.4
0.0 0.1 0.2
Single hs-cTn measurement
Serial hs-cTn measurement

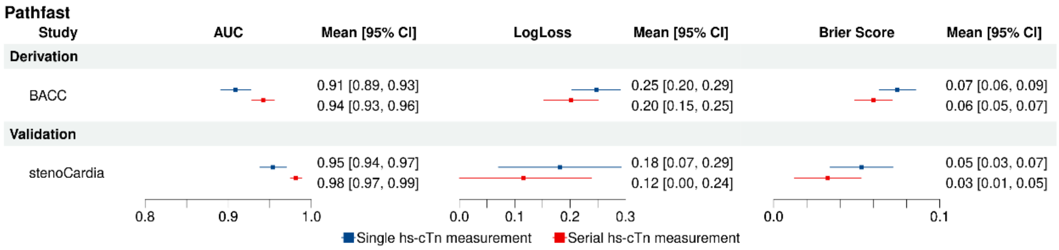
Pathfast
Study
AUC
Mean [95% CI]
LogLoss
Mean [95% CI]
Brier Score
Mean [95% CI]
Derivation
BACC
0.91 [0.89, 0.93]
0.94 [0.93, 0.96]
0.25 [0.20, 0.29]
0.20 [0.15, 0.25]
0.07 [0.06, 0.09]
0.06 [0.05, 0.07]
Validation
stenoCardia
0.95 [0.94, 0.97]
0.98 [0.97, 0.99]
0.18 [0.07, 0.29]
0.12 [0.00, 0.24]
0.05 [0.03, 0.07]
0.03 [0.01, 0.05]
0.8 0.9 1.0
0.0 0.1 0.2 0.3
0.0 0.1
Single hs-cTn measurement
Serial hs-cTn measurement

Figure S6: Calibration plot for single and serial hs-cTn measurement per assay and for derivation and validation datasets

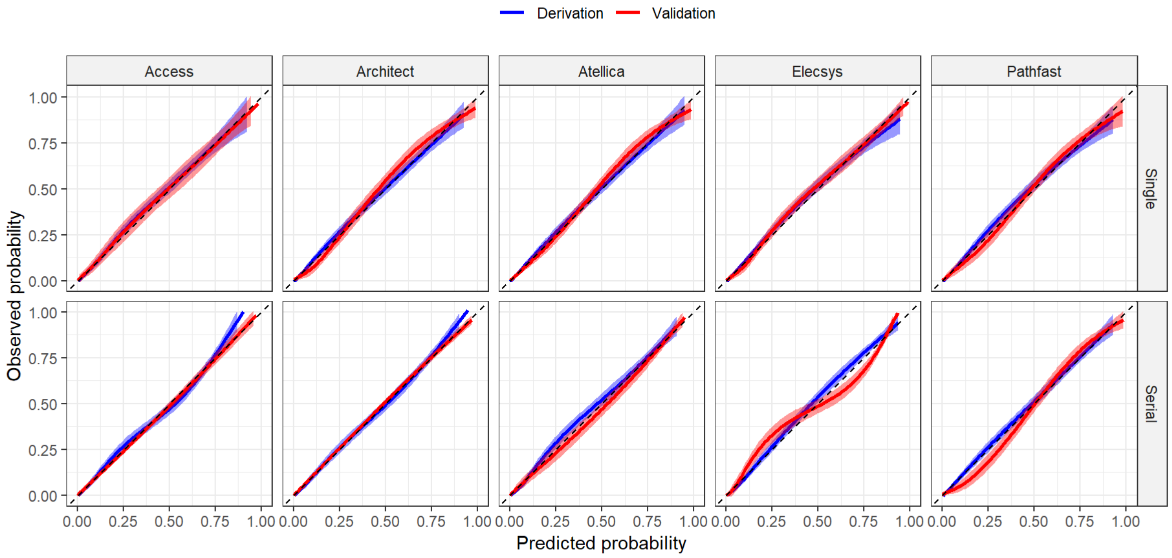

Predicted probabilities of myocardial infarction (x-axis) and observed probabilities of myocardial infarction (y-axis) obtained via smoothing techniques. The dashed line is the identity line (y = x), perfectly calibrated predictions would be at this line. In the validation cohort. probability estimates were calibrated using logistic regression with restricted cubic splines.

Figure S7: Calibration plot for single and serial hs-cTn measurement per assay and the generalization datasets

Access

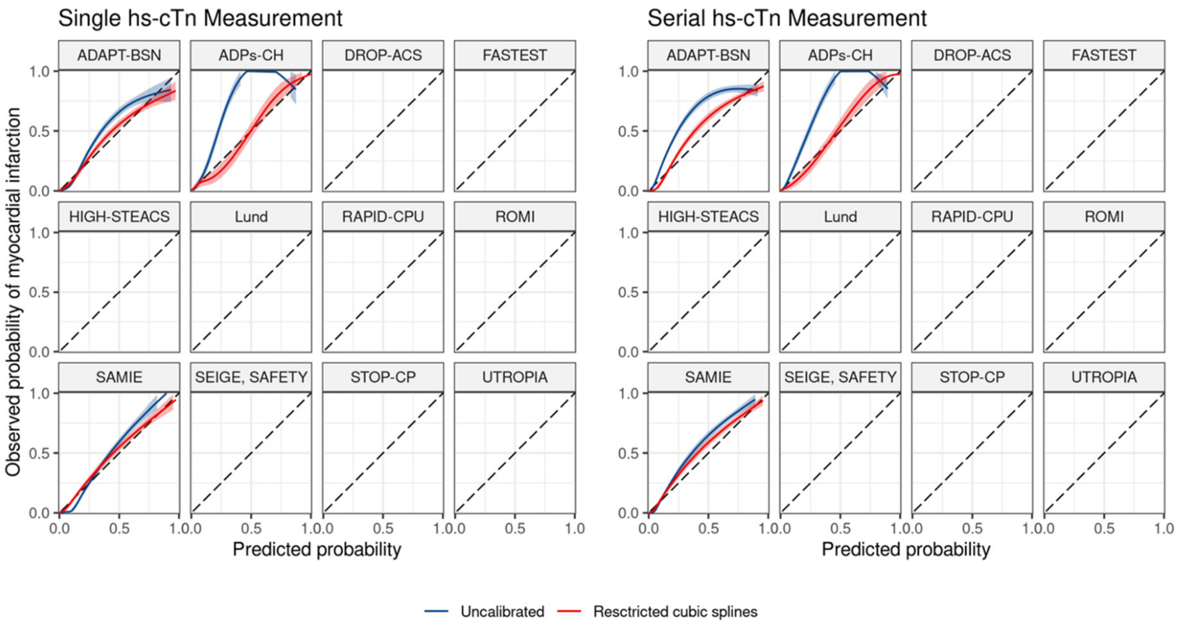

Architect

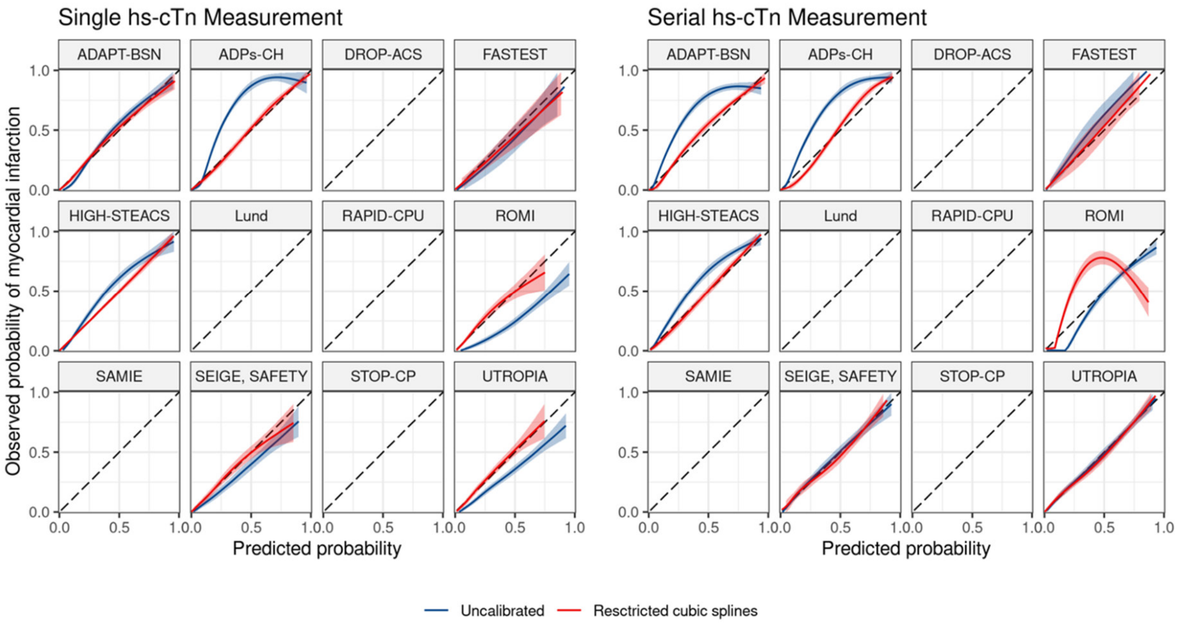

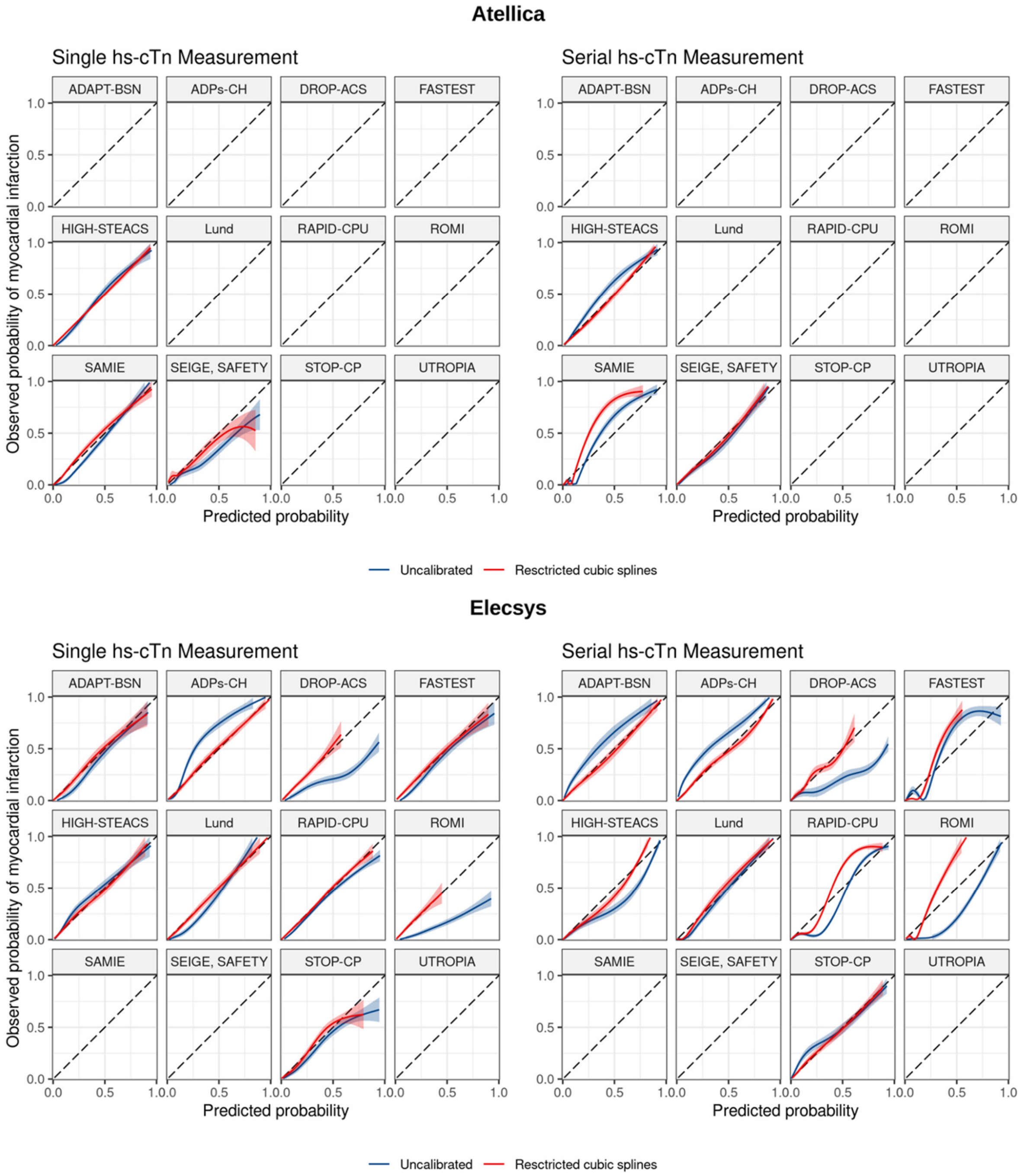

Predicted probabilities of myocardial infarction (x-axis) and observed probabilities of myocardial infarction (y-axis) obtained via smoothing techniques. The dashed line is the identity line (y = x), perfectly calibrated predictions would be at this line.

Figure S8: Diagnostic accuracy using the myocardial infarction probability as threshold

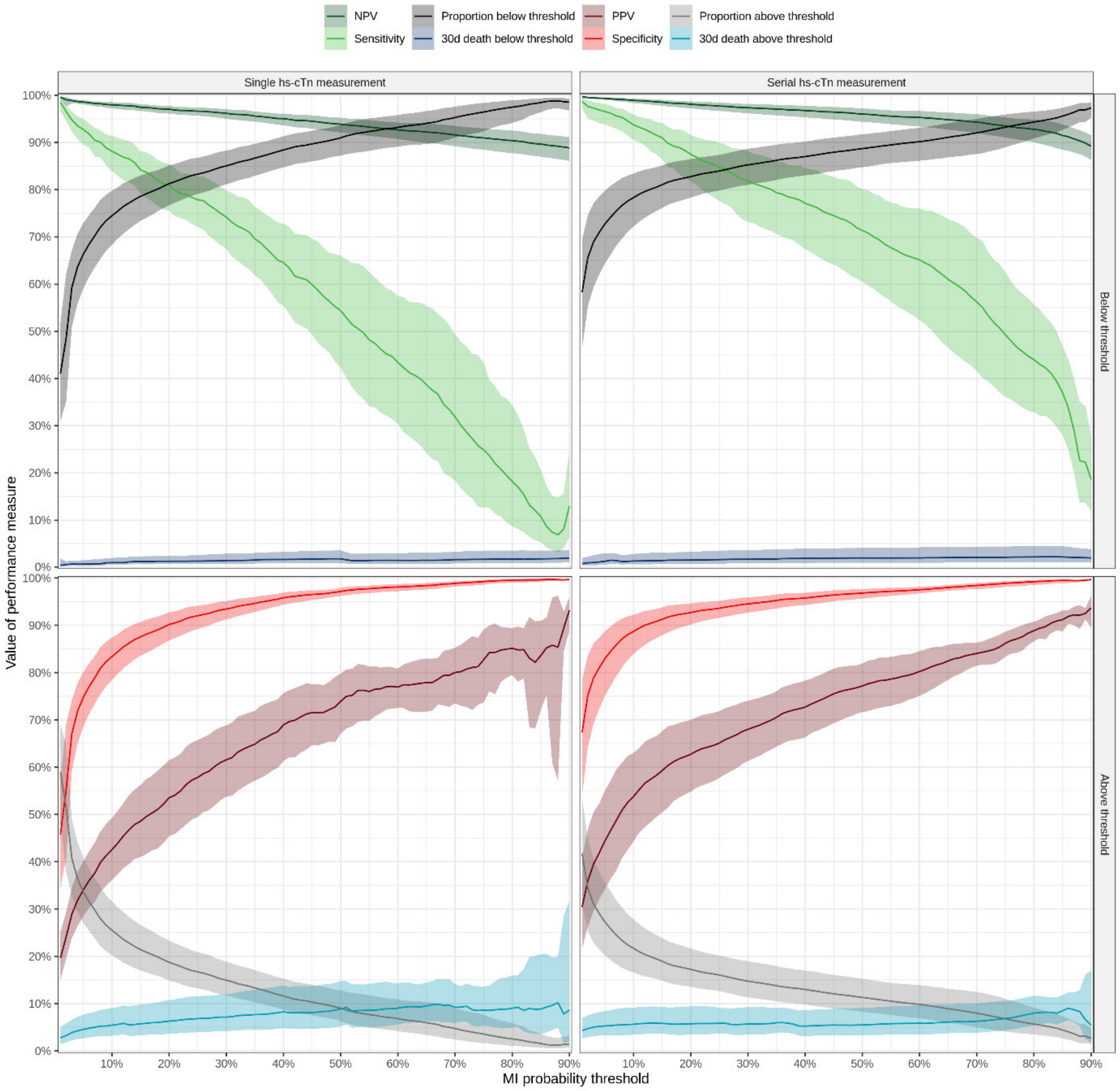

This figure displays the diagnostic performance measures (negative predictive value (NPV), positive predictive value (PPV), sensitivity, specificity, and proportion of individuals) using the myocardial infarction probability as threshold. Detailed diagnostic measures are displayed in Tables S7 and S8 for each hs-cTn assay. As an example, this figure is based on hs-cTnT concentrations using the Elecsys assay, as this assay had the highest availability. This figure is based on data from the validation cohort stenocardia as well as the generalization cohorts ADAPT-BSN, ADPs-CH, FASTEST, LUND, RAPID-CPU, SAMIE, SEIGE & SAFETY, STOP-CP and UTROPIA.

Figure S9: Scatterplot and diagnostic performance using Atellica VTLi

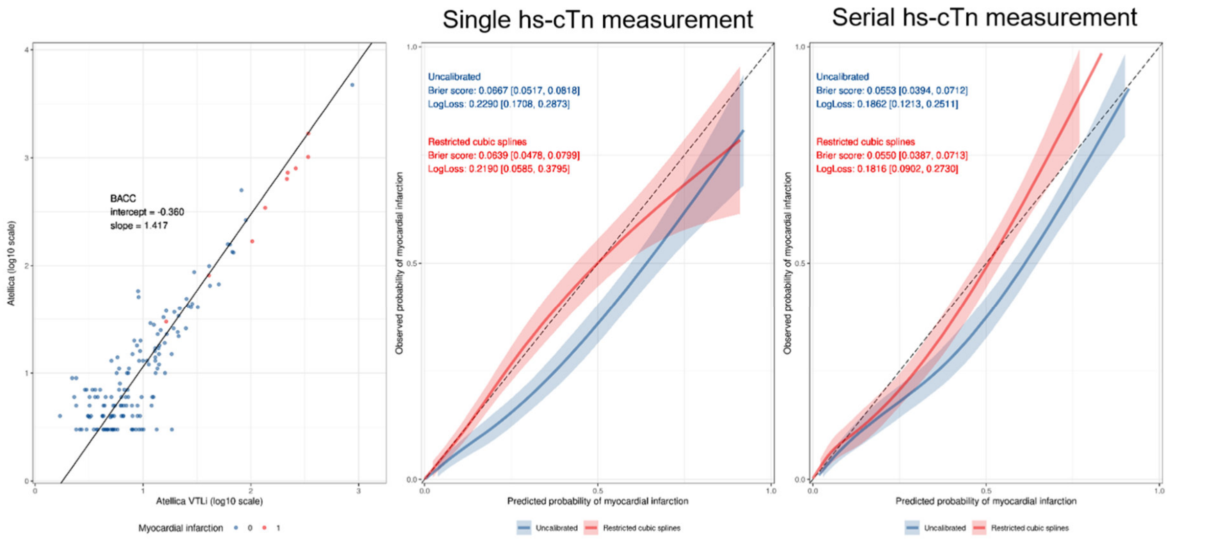

Calculation of the TLS in the BACC cohort

Calibration curves for Atellica VTLi in Seige & Safety (RCS calibration for the SL-EW)

The left-most figure shows the scatterplot for the Atellica hs-cTn measurements versus the Atellica VTLi hs-cTn measurements together with the regression line obtained from total least squares (TLS) regression for 135 patients from BACC. Subjects from without myocardial infarction (MI) are displayed in blue, patients with MI are displayed in red. Calibration curves are shown for restricted cubic spline re-calibration (red line; red shaded area shows 95% confidence intervals) and no calibration (blue line; blue shaded area shows 95% confidence intervals) for the Atellica VTLi hs-cTn in SIEGE & Safety. The black dashed line shows the ideal relationship between observed MI probabilities (y-axis) and predicted MI probabilities (x-axis). In both cases, the rcs-calibrated (red) curves are closer to the black dotted line the uncalibrated (blue) curves.

Figure S10: Clinical use cases

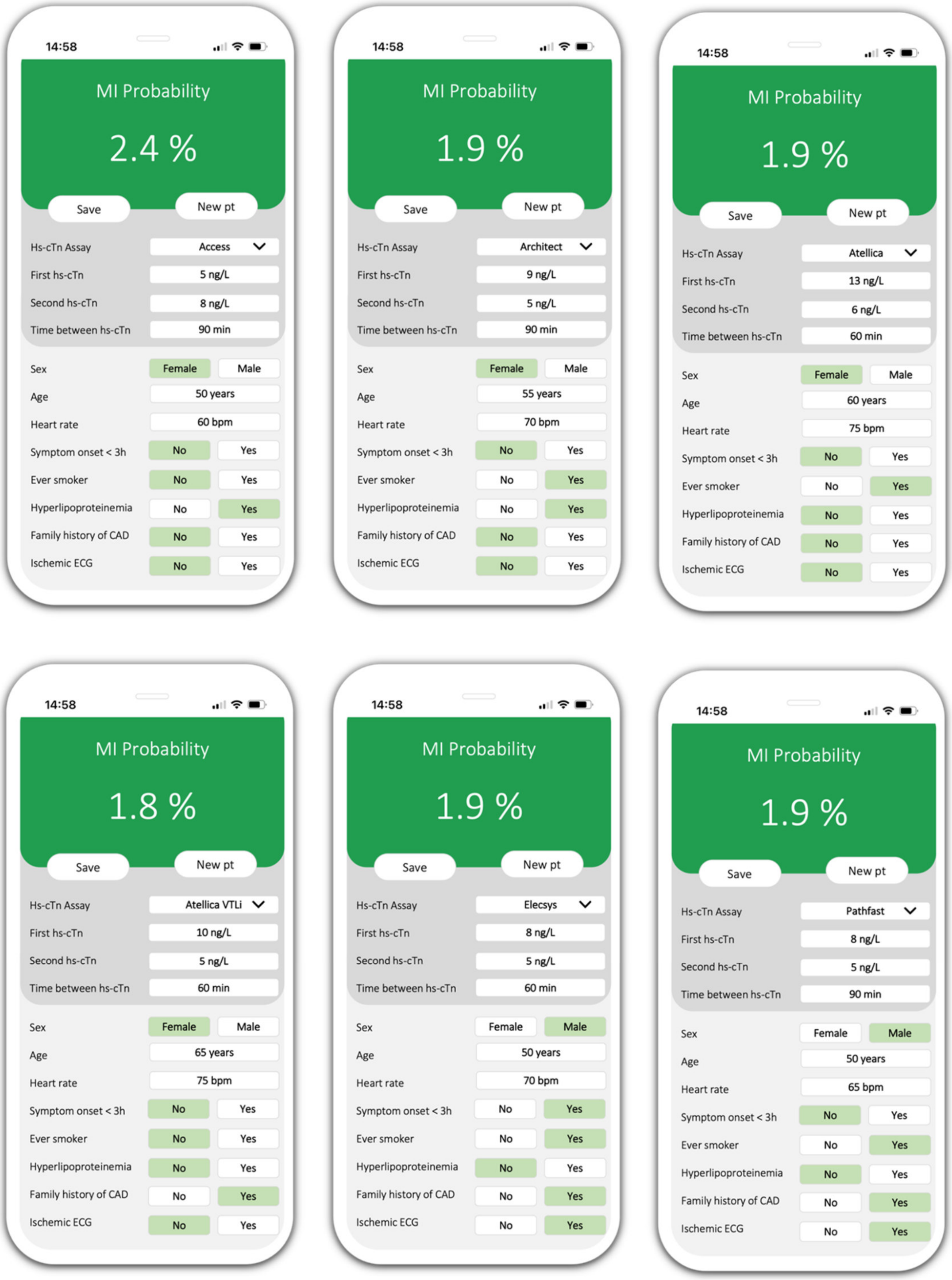

This figure illustrates the clinical application of the machine-learning algorithm to determine the individual probability of having an acute MI.

2. Supplemental Tables

Table S1: TRIPOD Checklist

Section/Topic			Checklist Item	Page
**Title and abstract**				
Title	1	D;V	Identify the study as developing and/or validating a multivariable prediction model, the target population, and the outcome to be predicted.	1
Abstract	2	D;V	Provide a summary of objectives, study design, setting, participants, sample size, predictors, outcome, statistical analysis, results, and conclusions.	3
**Introduction**				
Background and objectives	3a	D;V	Explain the medical context (including whether diagnostic or prognostic) and rationale for developing or validating the multivariable prediction model, including references to existing models.	5
	3b	D;V	Specify the objectives, including whether the study describes the development or validation of the model or both.	5
**Methods**				
Source of data	4a	D;V	Describe the study design or source of data (e.g., randomized trial, cohort, or registry data), separately for the development and validation data sets, if applicable.	6-7
	4b	D;V	Specify the key study dates, including start of accrual; end of accrual; and, if applicable, end of follow-up.	S5-10
Participants	5a	D;V	Specify key elements of the study setting (e.g., primary care, secondary care, general population) including number and location of centres.	6
	5b	D;V	Describe eligibility criteria for participants.	6, S5-10
	5c	D;V	Give details of treatments received, if relevant.	n/a
Outcome	6a	D;V	Clearly define the outcome that is predicted by the prediction model, including how and when assessed.	6
	6b	D;V	Report any actions to blind assessment of the outcome to be predicted.	n/a
Predictors	7a	D;V	Clearly define all predictors used in developing or validating the multivariable prediction model, including how and when they were measured.	7, S11
	7b	D;V	Report any actions to blind assessment of predictors for the outcome and other predictors.	n/a
Sample size	8	D;V	Explain how the study size was arrived at.	S19
Missing data	9	D;V	Describe how missing data were handled (e.g., complete-case analysis, single imputation, multiple imputation) with details of any imputation method.	S18-19
Statistical analysis methods	10a	D	Describe how predictors were handled in the analyses.	S12-17
	10b	D	Specify type of model, all model-building procedures (including any predictor selection), and method for internal validation.	S12-17
	10c	V	For validation, describe how the predictions were calculated.	S12-17
	10d	D;V	Specify all measures used to assess model performance and, if relevant, to compare multiple models.	S12-17
	10e	V	Describe any model updating (e.g., recalibration) arising from the validation, if done.	S12-17
Risk groups	11	D;V	Provide details on how risk groups were created, if done.	n/a
Development vs. validation	12	V	For validation, identify any differences from the development data in setting, eligibility criteria, outcome, and predictors.	S5-10

**Results**				
Participants	13a	D;V	Describe the flow of participants through the study, including the number of participants with and without the outcome and, if applicable, a summary of the follow-up time. A diagram may be helpful.	Figure S1
	13b	D;V	Describe the characteristics of the participants (basic demographics, clinical features, available predictors), including the number of participants with missing data for predictors and outcome.	Table 1, Tables S2-4
	13c	V	For validation, show a comparison with the development data of the distribution of important variables (demographics, predictors and outcome).	Tables S2-4
Model development	14a	D	Specify the number of participants and outcome events in each analysis.	Tables S2-4
	14b	D	If done, report the unadjusted association between each candidate predictor and outcome.	n/a
Model specification	15a	D	Present the full prediction model to allow predictions for individuals (i.e., all regression coefficients, and model intercept or baseline survival at a given time point).	n/a
	15b	D	Explain how to the use the prediction model.	S12-17
Model performance	16	D;V	Report performance measures (with CIs) for the prediction model.	Table S7
Model-updating	17	V	If done, report the results from any model updating (i.e., model specification, model performance).	n/a
**Discussion**				
Limitations	18	D;V	Discuss any limitations of the study (such as nonrepresentative sample, few events per predictor, missing data).	12-14
Interpretation	19a	V	For validation, discuss the results with reference to performance in the development data, and any other validation data.	12-14
	19b	D;V	Give an overall interpretation of the results, considering objectives, limitations, results from similar studies, and other relevant evidence.	12-14
Implications	20	D;V	Discuss the potential clinical use of the model and implications for future research.	12-14
**Other information**				
Supplementary information	21	D;V	Provide information about the availability of supplementary resources, such as study protocol, Web calculator, and data sets.	Supplement
Funding	22	D;V	Give the source of funding and the role of the funders for the present study.	16

Table S2: Baseline characteristics for derivation, validation and generalization cohorts including information on missingness

	All Patients		Derivation		Validation		Global generalization		
		# missing		*# missing*		*# missing*	**Total**	*# missing*	**(lowest – highest per cohort)**
**Sample size**	27674		2575		1688		23411		1033 - 4938
**Age (years)**	61 [50, 73]	*12*	64 [51, 75]	*0*	63 [52, 72]	*0*	61 [50, 73]	*12*	51 [43, 61] - 72 [60, 81]
**Sex (male) (%)**	15451 (55.8)	*8*	1638 (63.6)	*0*	1108 (65.6)	*0*	12705 (54.3)	*8*	1178 (37.8) - 859 (60.5)
**Heart rate (bpm)**	76 [66, 88]	*2315*	77 [67, 88.5]	*20*	70 [62, 81]	*15*	76 [66, 88]	*2280*	70 [62, 81] - 88 [74, 103]
**Systolic BP (mmHg)**	143 [128, 160]	*2581*	147 [131, 163]	*25*	140 [129, 160]	*11*	143 [128, 160]	*2545*	130 [113, 147] - 150 [138, 165]
**eGFR (mL/min for 1.73m²)**	82.9 [63.4, 96.9]	*1344*	76.9 [58.5, 92.3]	*20*	84.2 [69.0, 95.2]	*6*	83.4 [63.7, 97.4]	*1318*	65.3 [53.4, 83.0] - 94.3 [78.9, 104.9]
**History of CAD (%)**	8203 (29.8)	*178*	372 (33.9)	*0*	606 (36.9)	*44*	6725 (28.9)	*134*	63 (2.7) - 259 (19.5)
**History of heart failure (%)**	2588 (11.5)	*5085*	394 (15.3)	*0*	120 (7.5)	*85*	2074 (11.3)	*5000*	139 (13.5) - 647 (37.4)
**History of atrial fibrillation (%)**	1859 (13.0)	*13351*	395 (15.3)	*1*	162 (9.8)	*39*	1302 (12.9)	*13311*	213 (20.6) - 692 (84.6)
**Hypertension (%)**	16127 (59.0)	*336*	1681 (65.5)	*7*	1256 (74.4)	*0*	13190 (57.1)	*329*	82 (6.7) - 318 (24.2)
**Hyperlipoproteinemia (%)**	12837 (48.4)	*1162*	904 (35.1)	*0*	1236 (73.2)	*0*	10697 (48.1)	*1162*	691 (40.7) - 957 (70.9)
**Diabetes (%)**	5404 (19.8)	*403*	326 (12.8)	*22*	303 (18.2)	*23*	4775 (20.7)	*358*	262 (22.5) - 946 (66.9)
**Ever smoker (%)**	10796 (43.1)	*2653*	1187 (46.8)	*36*	865 (52.6)	*44*	8744 (42.0)	*2573*	280 (12.1) - 361 (34.9)
**Family history of CAD (%)**	8476 (40.0)	*6491*	478 (19.3)	*95*	540 (33.2)	*62*	7458 (43.7)	*6334*	460 (14.8) - 821 (63.2)
**Ischaemic signs ECG (%)**	4428 (18.4)	*3607*	520 (20.8)	*69*	872 (52.1)	*15*	3036 (15.3)	*3523*	20 (0.9) - 588 (36.1)
**Symptom onset < 3h (%)**	11122 (46.1)	*3574*	713 (29.4)	*147*	631 (37.4)	*0*	9778 (48.9)	*3427*	643 (27.5) - 2263 (98.1)
**Time between serial samples (min)**	80 [60, 155]	*2152*	60 [60, 63]	*38*	180 [162, 190]	*50*	90 [60, 148]	*2064*	60 [60, 60] - 204 [173, 264]
**Final diagnosis of MI (%)**	3249 (11.7)	*0*	368 (14.3)	*0*	283 (16.8)	*0*	2598 (11.1)	*0*	109 (5.5) - 519 (16.6)
**30d incident all-cause death (%)**	643 (2.7)	*3964*	74 (3.4)	*389*	38 (2.3)	*30*	531 (2.7)	*3545*	2 (0.1) - 7 (12.3)
**30d incident MI (%)**	1007 (4.8)	*6708*	24 (1.1)	*389*	47 (2.8)	*30*	936 (5.5)	*6289*	5 (0.3) - 433 (13.9)
**Hs-cTnI Access- First meas. (ng/L)**	3.5 [2.3, 8.3]	*20728*	5.3 [2.9, 15.8]	*696*	5.2 [2.3, 23.1]	*1018*	3.0 [2.3, 6.0]	*19014*	2.5 [2.3, 4.6] - 3.5 [2.3, 9.3]
**Hs-cTnI Access - Second meas. (ng/L)**	4.0 [2.3, 10.3]	*20664*	5.8 [3.0, 19.7]	*239*	7.2 [3.2, 38.6]	*1213*	3.0 [2.3, 6.5]	*19212*	2.6 [2.3, 4.9] - 4.0 [2.3, 8.0]
**Hs-cTnI Architect- First meas. (ng/L)**	4.5 [2.0, 14.0]	*13419*	5.7 [2.6, 16.1]	*81*	6.9 [3.5, 28.8]	*90*	4.0 [2.0, 12.0]	*13248*	2.3 [1.9, 4.4] - 7.0 [4.0, 21.0]
**Hs-cTnI Architect - Second meas. (ng/L)**	5.0 [2.2, 16.3]	*13732*	5.9 [2.6, 19.4]	*21*	7.8 [3.6, 35.8]	*217*	4.1 [2.0, 14.0]	*13494*	2.4 [1.9, 4.8] - 8.0 [4.0, 24.0]
**Hs-cTnI Atellica - First meas. (ng/L)**	5.3 [2.5, 17.4]	*19342*	5.7 [2.5, 19.9]	*391*	6.4 [3.0, 31.2]	*197*	4.8 [2.5, 14.6]	*18754*	3.5 [2.5, 8.5] - 10.7 [4.4, 29.1]
**Hs-cTnI Atellica- Second meas. (ng/L)**	6.4 [2.9, 22.0]	*19841*	6.2 [2.7, 23.9]	*168*	8.0 [3.5, 41.2]	*281*	6.0 [2.8, 18.8]	*19392*	4.5 [2.5, 11.2] - 11.9 [4.7, 32.6]
**Hs-cTnI Atellica VTLi- First meas. (ng/L)**	7.6 [4.1, 16.0]	*26586*	6.3 [3.8, 11.5]	*2459*	-	-	7.8 [4.1, 16.8]	*22439*	7.8 [4.1, 16.8]
**Hs-cTnI Atellica VTLi- Second meas. (ng/L)**	7.9 [4.1, 17.2]	*26592*	6.0 [3.8, 12.5]	*2462*	-	-	8.2 [4.2, 18.4]	*22442*	8.2 [4.2, 18.4]
**Hs-cTnT Elecsys - First meas. (ng/L)**	9.0 [5.0, 20.0]	*7673*	9.0 [5.0, 21.0]	*14*	9.2 [5.0, 20.3]	*140*	8.6 [5.0, 20.0]	*7519*	6.0 [4.0, 13.0] - 16.0 [8.0, 36.0]
**Hs-cTnT Elecsys - Second meas. (ng/L)**	9.0 [5.0, 22.0]	*9148*	9.0 [5.0, 23.0]	*5*	8.1 [4.1, 23.3]	*162*	8.8 [5.0, 22.0]	*8981*	3.0 [3.0, 12.0] - 18.0 [8.2, 42.0]
**Hs-cTnI Pathfast- First meas. (ng/L)**	4.0 [2.3, 12.4]	*24428*	3.7 [2.3, 12.2]	*315*	4.2 [2.3, 12.8]	*702*	-	-	-
**Hs-cTnI Pathfast- Second meas. (ng/L)**	4.3 [2.3, 15.5]	*24360*	4.0 [2.3, 14.4]	*124*	5.1 [2.6, 20.3]	*825*	-	-	-

Data are presented as median [Q1, Q3] or number (proportion). * VTLi measurements were performed in a separate population of patients recruited to the BACC study. Detailed characteristics of these patients is provided in Table S2. Abbreviations: BP = blood pressure, eGFR = estimated glomerular filtration rate, CAD = coronary artery disease, ECG = electrocardiogram, MI = myocardial infarction, hs-cTn = high-sensitivity cardiac troponin, meas. = measurement.

Table S3: Baseline characteristics for all generalization cohorts

	ADAPT-BSN	ADPs-CH	BACC VTli	DROP-ACS	FASTEST	High-STEACS	Lund
**Sample size**	2315	3119	125	1420	1233	1750	1164
**Age (years)**	51.0 [43.0, 61.0]	62.1 [53.0, 72.4]	57 [50, 72]	72.0 [60.0, 81.0]	64.0 [53.0, 73.0]	60.0 [51.0, 72.0]	63.0 [49.0, 74.0]
**Sex (male) (%)**	1386 (59.9)	1178 (37.8)	73 (60.8)	859 (60.5)	677 (55.0)	1048 (59.9)	636 (54.6)
**Heart rate (bpm)**	75.0 [65.0, 86.0]	70.0 [62.0, 81.0]	77 [64, 90]	79.0 [68.0, 91.0]	70.0 [61.0, 82.0]	75.0 [64.0, 87.0]	79.0 [70.0, 90.0]
**Systolic BP (mmHg)**	136.0 [122.0, 150.0]	143.0 [128.0, 162.0]	142 [128, 164]	148.0 [130.0, 167.0]	145.0 [130.0, 163.0]	137.0 [124.0, 153.0]	143.0 [128.0, 160.0]
**eGFR (mL/min for 1.73m²)**	94.3 [78.9, 104.9]	65.3 [53.4, 83.0]	87.0 [68.1, 99.0]	83.8 [62.4, 94.5]	86.7 [72.0, 96.9]	89.1 [74.7, 98.9]	83.8 [67.8, 95.8]
**History of CAD (%)**	383 (16.5)	1122 (36.0)	38 (30.4)	393 (27.7)	384 (31.2)	647 (37.4)	291 (25.0)
**History of heart failure (%)**	63 (2.7)	206 (6.6)	2 (6.2)	-	55 (4.5)	53 (3.2)	130 (11.2)
**History of atrial fibrillation (%)**	201 (8.7)	-	5 (15.6)	215 (15.1)	82 (6.7)	-	186 (16.0)
**Hypertension (%)**	1005 (43.4)	1708 (54.8)	21 (65.6)	911 (64.5)	546 (44.4)	691 (40.7)	504 (43.3)
**Hyperlipoproteinemia (%)**	915 (44.7)	1767 (56.7)	29 (23.2)	946 (66.9)	378 (30.8)	687 (41.0)	262 (22.5)
**Diabetes (%)**	280 (12.1)	477 (15.3)	-	397 (28.1)	162 (13.2)	252 (14.7)	162 (13.9)
**Ever smoker (%)**	1413 (61.0)	460 (14.8)	49 (39.2)	740 (59.8)	624 (50.7)	990 (58.5)	656 (56.5)
**Family history of CAD (%)**	945 (40.8)	1707 (54.8)	14 (13.1)	-	692 (84.6)	843 (51.8)	265 (23.1)
**Ischaemic signs ECG (%)**	20 (0.9)	321 (10.3)	18 (14.9)	150 (11.2)	252 (20.9)	193 (11.3)	69 (6.0)
**Symptom onset < 3h (%)**	2263 (98.1)	643 (27.5)	8 (21.6)	694 (60.4)	614 (50.8)	734 (46.4)	391 (34.2)
**Time between serial samples (min)**	135.0 [125.0, 150.0]	120.0 [90.0, 120.0]	66 (60, 80)	60.0 [60.0, 60.0]	71.0 [62.0, 105.0]	125.0 [67.0, 167.0]	60.0 [60.0, 60.0]
**Final diagnosis of MI (%)**	165 (7.1)	519 (16.6)	5 (4.0)	167 (11.8)	128 (10.4)	230 (13.1)	81 (7.0)
**30d incident all-cause death (%)**	2 (0.1)	108 (3.5)	-	-	3 (0.2)	44 (2.5)	6 (0.5)
**30d incident MI (%)**	114 (4.9)	433 (13.9)	-	-	8 (0.6)	5 (0.3)	11 (0.9)
**Hs-cTnI Access- First measurement (ng/L)**	2.5 [2.3, 4.6]	3.5 [2.3, 9.3]	-	-	-	-	-
**Hs-cTnI Access - Second measurement (ng/L)**	2.6 [2.3, 4.9]	3.5 [2.3, 8.9]	-	-	-	-	-
**Hs-cTnI Architect- First measurement (ng/L)**	2.3 [1.9, 4.4]	4.3 [2.2, 13.7]	-	-	4.3 [2.0, 8.8]	3.0 [1.9, 9.0]	-
**Hs-cTnI Architect - Second measurement (ng/L)**	2.4 [1.9, 4.8]	4.6 [2.3, 15.4]	-	-	4.0 [2.0, 9.8]	3.0 [1.9, 12.0]	-
**Hs-cTnI Atellica - First measurement (ng/L)**	-	-	4.0 [2.5, 13.0]	-	-	4.7 [2.5, 14.6]	-
**Hs-cTnI Atellica- Second measurement (ng/L)**	-	-	4.0 [2.5, 13.0]	-	-	5.0 [2.5, 17.9]	-
**Hs-cTnI Atellica VTLi- First measurement (ng/L)**	-	-	6.3 [3.8, 11.5]	-	-	-	-
**Hs-cTnI Atellica VTLi- Second measurement (ng/L)**	-	-	6.0 [3.8, 12.5]	-	-	-	-
**Hs-cTnT Elecsys - First measurement (ng/L)**	6.7 [4.3, 11.8]	7.9 [5.0, 18.4]	-	16.0 [8.0, 36.0]	7.0 [5.0, 14.0]	6.0 [4.0, 13.0]	7.0 [4.0, 15.0]
**Hs-cTnT Elecsys - Second measurement (ng/L)**	6.5 [4.1, 11.6]	7.5 [5.0, 18.1]	-	18.0 [8.2, 42.0]	7.0 [5.0, 15.0]	3.0 [3.0, 12.0]	7.0 [4.0, 16.0]

Data are presented as median [Q1, Q3] or number (proportion). Abbreviations: BP = blood pressure, eGFR = estimated glomerular filtration rate, CAD = coronary artery disease, ECG = electrocardiogram, MI = myocardial infarction, hs-cTn = high-sensitivity cardiac troponin.

Table S3: Baseline characteristics for all generalization cohorts (continued)

	RAPID-CPU	ROMI	SAMIE	SEIGE, SAFETY	STOP-CP	UTROPIA
**Sample size**	4938	1366	1985	1033	1457	1631
**Age (years)**	64.0 [51.0, 77.0]	68.0 [55.0, 80.0]	56.0 [46.0, 68.0]	58.0 [48.0, 69.0]	57.5 [49.1, 66.0]	57.0 [48.0, 67.0]
**Sex (male) (%)**	2867 (58.1)	650 (47.7)	1091 (55.0)	621 (60.1)	781 (53.6)	911 (55.9)
**Heart rate (bpm)**	77.0 [67.0, 87.0]	-	75.0 [66.0, 86.5]	88.0 [75.0, 102.0]	76.0 [66.0, 89.0]	88.0 [74.0, 103.0]
**Systolic BP (mmHg)**	150.0 [138.0, 165.0]	-	135.0 [123.0, 152.0]	134.0 [118.0, 152.0]	130.0 [113.0, 147.0]	142.0 [125.0, 158.0]
**eGFR (mL/min for 1.73m²)**	84.1 [64.8, 98.2]	76.9 [52.0, 91.2]	88.6 [73.1, 100.6]	72.6 [47.6, 93.0]	82.2 [63.3, 95.6]	79.3 [53.6, 98.0]
**History of CAD (%)**	1795 (36.4)	357 (27.2)	477 (24.3)	139 (13.5)	472 (32.4)	265 (16.5)
**History of heart failure (%)**	909 (18.4)	259 (19.5)	-	168 (16.3)	-	231 (14.3)
**History of atrial fibrillation (%)**	-	318 (24.2)	-	118 (11.4)	-	182 (11.2)
**Hypertension (%)**	3241 (68.8)	957 (70.9)	938 (47.6)	644 (62.3)	971 (66.6)	1074 (66.3)
**Hyperlipoproteinemia (%)**	2196 (52.1)	802 (60.3)	901 (46.0)	449 (43.5)	698 (47.9)	696 (43.1)
**Diabetes (%)**	1019 (21.8)	405 (30.4)	350 (17.6)	361 (34.9)	414 (28.4)	496 (30.7)
**Ever smoker (%)**	1010 (24.2)	821 (63.2)	444 (22.4)	617 (59.7)	-	969 (60.1)
**Family history of CAD (%)**	1236 (31.0)	714 (58.9)	843 (46.3)	213 (20.6)	-	-
**Ischaemic signs ECG (%)**	1319 (26.7)	-	-	35 (3.4)	89 (6.1)	588 (36.1)
**Symptom onset < 3h (%)**	2136 (43.3)	325 (45.8)	659 (52.2)	285 (27.6)	515 (35.5)	519 (60.3)
**Time between serial samples (min)**	60.0 [60.0, 60.0]	181.2 [178.2, 190.2]	185.0 [169.0, 198.0]	125.0 [115.8, 140.0]	54.0 [43.0, 65.0]	204.0 [172.8, 263.7]
**Final diagnosis of MI (%)**	674 (13.6)	81 (5.9)	109 (5.5)	98 (9.5)	176 (12.1)	170 (10.4)
**30d incident all-cause death (%)**	292 (6.0)	46 (3.4)	-	16 (1.6)	7 (0.5)	7 (0.4)
**30d incident MI (%)**	127 (2.9)	221 (16.2)	-	3 (3.3)	-	14 (0.9)
**Hs-cTnI Access- First measurement (ng/L)**	-	-	3.0 [2.3, 6.0]	-	-	-
**Hs-cTnI Access - Second measurement (ng/L)**	-	-	4.0 [2.3, 8.0]	-	-	-
**Hs-cTnI Architect- First measurement (ng/L)**	-	5.1 [2.0, 17.0]	-	7.0 [4.0, 21.0]	-	5.0 [1.9, 15.9]
**Hs-cTnI Architect - Second measurement (ng/L)**	-	6.0 [3.0, 20.0]	-	8.0 [4.0, 24.0]	-	6.4 [2.5, 21.0]
**Hs-cTnI Atellica - First measurement (ng/L)**	-	-	3.5 [2.5, 8.5]	10.7 [4.4, 29.1]	-	-
**Hs-cTnI Atellica- Second measurement (ng/L)**	-	-	4.5 [2.5, 11.2]	11.9 [4.7, 32.6]	-	-
**Hs-cTnI Atellica VTLi- First measurement (ng/L)**	-	-	-	7.8 [4.1, 16.8]	-	-
**Hs-cTnI Atellica VTLi- Second measurement (ng/L)**	-	-	-	8.2 [4.2, 18.4]	-	-
**Hs-cTnT Elecsys - First measurement (ng/L)**	9.0 [5.0, 21.0]	12.0 [6.0, 26.8]	-	-	9.2 [4.9, 21.1]	-
**Hs-cTnT Elecsys - Second measurement (ng/L)**	10.0 [6.0, 25.0]	12.0 [6.0, 26.0]	-	-	9.1 [5.0, 21.3]	-

Data are presented as median [Q1, Q3] or number (proportion). Abbreviations: BP = blood pressure, eGFR = estimated glomerular filtration rate, CAD = coronary artery disease, ECG = electrocardiogram, MI = myocardial infarction, hs-cTn = high-sensitivity cardiac troponin.

Table S4: Baseline characteristics for all generalization cohorts stratified by MI diagnosis

	ADAPT-BSN		ADPs-CH		DROP-ACS		FASTEST	
	**No MI**	**MI**	**No MI**	**MI**	**No MI**	**MI**	**No MI**	**MI**
**Sample size**	2150	165	2600	519	1253	167	1105	128
**Age (years)**	51.0 [42.0, 60.0]	64.0 [54.0, 76.0]	61.0 [52.0, 71.0]	69.2 [59.0, 77.5]	73.0 [60.0, 81.0]	69.0 [60.0, 78.0]	63.0 [53.0, 72.0]	70.0 [60.8, 78.0]
**Sex (male) (%)**	1281 (59.6)	105 (63.6)	1044 (40.2)	134 (25.8)	728 (58.1)	131 (78.4)	587 (53.3)	90 (70.3)
**Heart rate (bpm)**	75.0 [65.0, 85.0]	80.0 [68.0, 93.2]	70.0 [62.0, 81.0]	72.0 [62.0, 82.0]	79.0 [68.0, 92.0]	80.0 [69.0, 90.0]	70.0 [61.0, 81.0]	73.0 [61.0, 88.0]
**Systolic BP (mmHg)**	135.0 [122.0, 150.0]	139.0 [120.0, 155.0]	142.0 [128.0, 162.0]	146.0 [129.0, 166.0]	146.0 [130.0, 166.0]	153.0 [139.2, 174.0]	145.0 [130.0, 162.0]	150.0 [134.8, 170.0]
**eGFR (mL/min for 1.73m²)**	95.0 [80.9, 105.3]	76.8 [56.6, 93.8]	67.4 [55.5, 86.0]	57.0 [45.9, 69.4]	83.9 [62.9, 94.6]	82.0 [60.0, 93.6]	87.1 [73.2, 97.2]	81.4 [61.0, 95.0]
**History of CAD (%)**	317 (14.7)	66 (40.0)	903 (34.8)	219 (42.2)	326 (26.1)	67 (40.1)	340 (30.8)	44 (34.4)
**History of heart failure (%)**	50 (2.3)	13 (7.9)	166 (6.4)	40 (7.7)	-	0 (NaN)	51 (4.6)	4 (3.1)
**History of atrial fibrillation (%)**	168 (7.8)	33 (20.0)	-	0 (NaN)	203 (16.2)	12 (7.2)	68 (6.2)	14 (10.9)
**Hypertension (%)**	903 (42.0)	102 (61.8)	1390 (53.5)	318 (61.3)	783 (62.8)	128 (77.1)	474 (43.0)	72 (56.2)
**Hyperlipoproteinemia (%)**	830 (43.8)	85 (56.7)	1462 (56.3)	305 (58.8)	808 (64.7)	138 (83.6)	334 (30.3)	44 (34.6)
**Diabetes (%)**	245 (11.4)	35 (21.2)	372 (14.3)	105 (20.2)	329 (26.4)	68 (41.0)	140 (12.7)	22 (17.2)
**Ever smoker (%)**	1307 (60.8)	106 (64.2)	375 (14.4)	85 (16.4)	628 (58.5)	112 (68.7)	554 (50.3)	70 (54.7)
**Family history of CAD (%)**	868 (40.4)	77 (46.7)	1407 (54.2)	300 (57.8)	-	0 (NaN)	615 (84.6)	77 (84.6)
**Ischaemic signs ECG (%)**	16 (0.7)	4 (2.4)	166 (6.4)	155 (29.9)	110 (9.3)	40 (25.8)	206 (19.1)	46 (36.5)
**Symptom onset < 3h (%)**	2102 (98.1)	161 (97.6)	560 (28.9)	83 (20.8)	623 (60.3)	71 (61.7)	542 (50.0)	72 (58.5)
**Time between serial samples (min)**	135.0 [125.0, 150.0]	135.0 [125.0, 155.0]	120.0 [90.0, 120.0]	120.0 [102.2, 120.0]	60.0 [60.0, 60.0]	60.0 [60.0, 60.0]	70.0 [61.0, 95.0]	90.0 [64.8, 145.2]
**Final diagnosis of MI (%)**	0 (0.0)	165 (100.0)	0 (0.0)	519 (100.0)	0 (0.0)	167 (100.0)	0 (0.0)	128 (100.0)
**30d incident all-cause death (%)**	0 (0.0)	2 (1.2)	58 (2.2)	50 (9.6)	-	0 (NaN)	1 (0.1)	2 (1.6)
**30d incident MI (%)**	1 (0.0)	113 (68.5)	277 (10.7)	156 (30.1)	-	0 (NaN)	3 (0.3)	5 (3.9)
**Hs-cTnI Access- First measurement (ng/L)**	2.4 [2.3, 4.0]	29.7 [14.8, 148.5]	2.7 [2.3, 5.3]	112.0 [41.7, 410.5]	-	-	-	-
**Hs-cTnI Access - Second measurement (ng/L)**	2.4 [2.3, 4.0]	64.1 [28.9, 355.1]	2.8 [2.3, 5.1]	192.3 [66.5, 891.4]	-	-	-	-
**Hs-cTnI Architect- First measurement (ng/L)**	2.1 [1.9, 3.7]	40.5 [15.2, 225.4]	3.5 [2.0, 6.8]	109.2 [40.2, 641.8]	-	-	3.4 [2.0, 6.9]	91.5 [25.5, 582.0]
**Hs-cTnI Architect - Second measurement (ng/L)**	2.2 [1.9, 4.0]	75.3 [31.5, 366.4]	3.6 [2.1, 7.2]	212.4 [64.3, 1091.7]	-	-	3.3 [2.0, 7.4]	81.0 [39.0, 1635.0]
**Hs-cTnI Atellica - First measurement (ng/L)**	-	-	-	-	-	-	-	-
**Hs-cTnI Atellica- Second measurement (ng/L)**	-	-	-	-	-	-	-	-
**Hs-cTnI Atellica VTLi- First measurement (ng/L)**	-	-	-	-	-	-	-	-
**Hs-cTnI Atellica VTLi- Second measurement (ng/L)**	-	-	-	-	-	-	-	-
**Hs-cTnT Elecsys - First measurement (ng/L)**	6.2 [4.1, 9.5]	36.1 [21.4, 101.6]	6.3 [5.0, 11.2]	53.0 [26.5, 151.1]	13.5 [7.0, 30.0]	45.0 [21.5, 181.5]	6.0 [5.0, 11.0]	37.0 [20.5, 91.0]
**Hs-cTnT Elecsys - Second measurement (ng/L)**	5.9 [4.0, 9.4]	51.8 [27.6, 129.3]	6.1 [5.0, 10.6]	65.6 [32.3, 199.1]	15.0 [7.8, 33.0]	68.0 [28.5, 233.0]	6.0 [5.0, 11.0]	36.0 [24.0, 114.0]

Data are presented as median [Q1, Q3] or number (proportion). Abbreviations: BP = blood pressure, eGFR = estimated glomerular filtration rate, CAD = coronary artery disease, ECG = electrocardiogram, MI = myocardial infarction hs-cTn = high-sensitivity cardiac troponin.

Table S4: Baseline characteristics for all generalization cohorts stratified by MI diagnosis (continued)

	High-STEACS		Lund		RAPID-CPU		ROMI	
	**No MI**	**MI**	**No MI**	**MI**	**No MI**	**MI**	**No MI**	**MI**
**Sample size**	1520	230	1083	81	4264	674	1285	81
**Age (years)**	59.0 [51.0, 70.0]	68.0 [58.0, 79.0]	63.0 [48.0, 73.0]	69.0 [61.0, 76.0]	63.0 [49.0, 76.0]	74.0 [62.0, 81.0]	67.0 [55.0, 80.0]	75.0 [58.0, 82.0]
**Sex (male) (%)**	896 (58.9)	152 (66.1)	583 (53.8)	53 (65.4)	2407 (56.4)	460 (68.2)	606 (47.2)	44 (54.3)
**Heart rate (bpm)**	75.0 [65.0, 87.0]	71.0 [62.0, 84.0]	79.0 [70.0, 90.0]	76.0 [67.0, 90.0]	76.0 [66.0, 86.0]	80.0 [70.0, 93.8]	-	-
**Systolic BP (mmHg)**	137.0 [124.2, 152.0]	138.0 [124.0, 153.0]	143.0 [128.0, 160.0]	148.0 [130.0, 175.0]	150.0 [138.0, 164.0]	152.0 [139.0, 166.0]	-	-
**eGFR (mL/min for 1.73m²)**	89.9 [76.0, 99.2]	84.5 [63.1, 95.4]	84.8 [68.4, 96.4]	73.8 [53.1, 88.8]	85.6 [67.5, 99.5]	72.8 [50.2, 88.5]	77.2 [52.3, 91.2]	69.3 [50.0, 88.0]
**History of CAD (%)**	555 (36.9)	92 (40.9)	259 (23.9)	32 (39.5)	1477 (34.7)	318 (47.2)	334 (26.9)	23 (31.1)
**History of heart failure (%)**	50 (3.4)	3 (1.4)	118 (10.9)	12 (14.8)	744 (17.4)	165 (24.5)	232 (18.6)	27 (35.5)
**History of atrial fibrillation (%)**	-	0 (NaN)	171 (15.8)	15 (18.5)	-	0 (NaN)	295 (23.8)	23 (30.3)
**Hypertension (%)**	579 (39.3)	112 (50.0)	452 (41.7)	52 (64.2)	2731 (66.9)	510 (80.7)	894 (70.4)	63 (78.8)
**Hyperlipoproteinemia (%)**	590 (40.5)	97 (44.1)	234 (21.6)	28 (34.6)	1828 (50.1)	368 (65.0)	747 (59.7)	55 (71.4)
**Diabetes (%)**	208 (13.9)	44 (19.6)	133 (12.3)	29 (35.8)	807 (20.0)	212 (33.4)	377 (30.0)	28 (36.8)
**Ever smoker (%)**	845 (57.4)	145 (65.9)	599 (55.5)	57 (70.4)	871 (24.1)	139 (25.2)	767 (62.6)	54 (73.0)
**Family history of CAD (%)**	730 (51.3)	113 (54.9)	245 (22.9)	20 (25.0)	1074 (30.9)	162 (31.7)	672 (58.8)	42 (60.9)
**Ischaemic signs ECG (%)**	126 (8.4)	67 (30.3)	39 (3.7)	30 (37.5)	1027 (24.1)	292 (43.3)	-	0 (NaN)
**Symptom onset < 3h (%)**	634 (46.1)	100 (48.3)	356 (33.5)	35 (43.8)	1780 (41.7)	356 (52.8)	297 (44.8)	28 (59.6)
**Time between serial samples (min)**	121.0 [65.0, 162.2]	149.0 [103.0, 194.5]	60.0 [60.0, 60.0]	60.0 [60.0, 60.0]	60.0 [60.0, 60.0]	60.0 [60.0, 60.0]	181.2 [177.0, 190.2]	184.8 [180.0, 197.2]
**Final diagnosis of MI (%)**	0 (0.0)	230 (100.0)	0 (0.0)	81 (100.0)	0 (0.0)	674 (100.0)	0 (0.0)	81 (100.0)
**30d incident all-cause death (%)**	26 (1.7)	18 (7.8)	5 (0.5)	1 (1.2)	193 (4.6)	99 (14.8)	39 (3.0)	7 (8.6)
**30d incident MI (%)**	1 (0.1)	4 (1.7)	7 (0.6)	4 (4.9)	80 (2.1)	47 (7.6)	208 (16.2)	13 (16.0)
**Hs-cTnI Access- First meas. (ng/L)**	-	-	-	-	-	-	-	-
**Hs-cTnI Access - Second meas. (ng/L)**	-	-	-	-	-	-	-	-
**Hs-cTnI Architect- First meas. (ng/L)**	2.0 [1.9, 5.0]	74.0 [24.0, 418.0]	-	-	-	-	5.0 [2.0, 14.5]	69.0 [20.5, 204.5]
**Hs-cTnI Architect - Second meas. (ng/L)**	3.0 [1.9, 6.0]	186.0 [61.0, 946.0]	-	-	-	-	5.0 [2.1, 16.0]	323.5 [102.5, 814.0]
**Hs-cTnI Atellica - First meas. (ng/L)**	3.8 [2.5, 8.1]	127.6 [51.4, 682.6]	-	-	-	-	-	-
**Hs-cTnI Atellica- Second meas. (ng/L)**	4.2 [2.5, 8.8]	311.5 [109.9, 1281.3]	-	-	-	-	-	-
**Hs-cTnI Atellica VTLi- First meas. (ng/L)**	-	-	-	-	-	-	-	-
**Hs-cTnI Atellica VTLi- Second meas. (ng/L)**	-	-	-	-	-	-	-	-
**Hs-cTnT Elecsys - First meas. (ng/L)**	5.0 [4.0, 10.0]	34.0 [17.0, 97.0]	6.0 [4.0, 13.0]	73.0 [24.0, 136.0]	8.0 [5.0, 15.0]	57.0 [24.0, 148.8]	11.0 [6.0, 25.0]	50.0 [27.0, 90.0]
**Hs-cTnT Elecsys - Second meas. (ng/L)**	3.0 [3.0, 6.0]	186.0 [61.0, 946.0]	6.0 [4.0, 13.0]	79.0 [31.0, 170.0]	9.0 [5.0, 16.0]	78.0 [39.0, 193.0]	11.0 [6.0, 23.0]	96.0 [57.5, 198.0]

Data are presented as median [Q1, Q3] or number (proportion). Abbreviations: BP = blood pressure, eGFR = estimated glomerular filtration rate, CAD = coronary artery disease, ECG = electrocardiogram, MI = myocardial infarction hs-cTn = high-sensitivity cardiac troponin, meas. = measurement.

Table S4: Baseline characteristics for all generalization cohorts stratified by MI diagnosis (continued)

	SAMIE		SEIGE, SAFETY		STOP-CP		UTROPIA	
	**No MI**	**MI**	**No MI**	**MI**	**No MI**	**MI**	**No MI**	**MI**
**Sample size**	1876	109	935	98	1281	176	1461	170
**Age (years)**	56.0 [45.0, 67.0]	68.0 [57.0, 77.0]	58.0 [48.0, 69.0]	58.0 [50.0, 67.8]	56.7 [48.7, 65.0]	62.4 [53.6, 70.6]	56.0 [47.0, 66.0]	60.0 [51.2, 70.5]
**Sex (male) (%)**	1016 (54.2)	75 (68.8)	560 (59.9)	61 (62.2)	660 (51.5)	121 (68.8)	826 (56.5)	85 (50.0)
**Heart rate (bpm)**	75.0 [66.0, 86.0]	79.0 [67.0, 91.0]	87.0 [74.0, 101.0]	96.5 [80.2, 115.0]	76.0 [66.0, 88.0]	78.0 [66.0, 89.8]	87.0 [74.0, 102.0]	95.0 [76.0, 115.0]
**Systolic BP (mmHg)**	135.0 [123.0, 152.0]	142.0 [123.0, 156.0]	133.0 [117.8, 151.0]	139.0 [121.8, 157.8]	130.0 [113.0, 147.0]	130.5 [117.0, 145.5]	141.0 [124.0, 157.0]	150.0 [126.0, 173.0]
**eGFR (mL/min for 1.73m²)**	89.6 [74.2, 101.3]	74.6 [61.1, 88.4]	73.4 [49.3, 93.4]	58.8 [31.6, 84.2]	83.4 [65.3, 97.0]	73.5 [46.7, 88.8]	81.2 [55.3, 99.9]	61.9 [39.2, 85.0]
**History of CAD (%)**	429 (23.1)	48 (45.3)	110 (11.8)	29 (29.6)	383 (29.9)	89 (50.6)	225 (15.6)	40 (24.1)
**History of heart failure (%)**	-	0 (NaN)	140 (15.0)	28 (28.6)	-	0 (NaN)	201 (13.9)	30 (18.0)
**History of atrial fibrillation (%)**	-	0 (NaN)	101 (10.8)	17 (17.3)	-	0 (NaN)	152 (10.5)	30 (18.0)
**Hypertension (%)**	865 (46.4)	73 (67.0)	576 (61.6)	68 (69.4)	837 (65.3)	134 (76.1)	947 (65.2)	127 (75.6)
**Hyperlipoproteinemia (%)**	834 (45.1)	67 (62.6)	392 (41.9)	57 (58.2)	597 (46.6)	101 (57.4)	612 (42.3)	84 (50.3)
**Diabetes (%)**	315 (16.8)	35 (32.1)	323 (34.5)	38 (38.8)	347 (27.1)	67 (38.1)	436 (30.1)	60 (35.7)
**Ever smoker (%)**	421 (22.5)	23 (21.1)	555 (59.4)	62 (63.3)	-	0 (NaN)	866 (60.1)	103 (60.6)
**Family history of CAD (%)**	804 (46.7)	39 (39.0)	191 (20.4)	22 (22.4)	-	0 (NaN)	-	0 (NaN)
**Ischaemic signs ECG (%)**	-	0 (NaN)	22 (2.4)	13 (13.3)	67 (5.2)	22 (12.5)	480 (32.9)	108 (63.5)
**Symptom onset < 3h (%)**	621 (52.2)	38 (52.1)	256 (27.4)	29 (29.6)	447 (35.1)	68 (38.9)	459 (60.5)	60 (58.8)
**Time between serial samples (min)**	185.0 [167.0, 198.8]	181.0 [176.0, 195.0]	125.0 [115.0, 140.0]	125.0 [120.0, 140.8]	54.0 [42.8, 64.2]	54.0 [43.0, 65.0]	205.2 [172.8, 262.3]	200.1 [169.3, 268.7]
**Final diagnosis of MI (%)**	0 (0.0)	109 (100.0)	0 (0.0)	98 (100.0)	0 (0.0)	176 (100.0)	0 (0.0)	170 (100.0)
**30d incident all-cause death (%)**	-	0 (NaN)	13 (1.4)	3 (3.3)	2 (0.2)	5 (2.8)	5 (0.4)	2 (1.2)
**30d incident MI (%)**	-	0 (NaN)	3 (3.8)	0 (0.0)	-	0 (NaN)	11 (0.8)	3 (1.8)
**Hs-cTnI Access- First measurement (ng/L)**	3.0 [2.3, 6.0]	63.0 [25.0, 297.0]	-	-	-	-	-	-
**Hs-cTnI Access - Second measurement (ng/L)**	4.0 [2.3, 6.0]	155.0 [37.5, 644.0]	-	-	-	-	-	-
**Hs-cTnI Architect- First measurement (ng/L)**	-	-	6.0 [4.0, 16.8]	67.0 [24.5, 176.0]	-	-	4.1 [1.9, 11.4]	42.7 [17.3, 128.4]
**Hs-cTnI Architect - Second measurement (ng/L)**	-	-	7.0 [4.0, 17.0]	96.0 [42.0, 349.8]	-	-	5.2 [2.3, 13.7]	116.2 [44.0, 578.3]
**Hs-cTnI Atellica - First measurement (ng/L)**	3.3 [2.5, 7.1]	99.4 [29.7, 416.1]	8.9 [4.1, 22.7]	79.3 [36.3, 241.4]	-	-	-	-
**Hs-cTnI Atellica- Second measurement (ng/L)**	4.2 [2.5, 8.7]	242.5 [57.1, 959.4]	9.8 [4.3, 22.3]	126.7 [58.3, 499.8]	-	-	-	-
**Hs-cTnI Atellica VTLi- First measurement (ng/L)**	-	-	7.2 [4.0, 13.8]	36.9 [18.6, 118.2]	-	-	-	-
**Hs-cTnI Atellica VTLi- Second measurement (ng/L)**	-	-	7.2 [3.9, 14.2]	64.2 [28.4, 184.0]	-	-	-	-
**Hs-cTnT Elecsys - First measurement (ng/L)**	-	-	-	-	8.0 [4.6, 15.5]	52.9 [28.4, 109.4]	-	-
**Hs-cTnT Elecsys - Second measurement (ng/L)**	-	-	-	-	7.8 [4.8, 15.2]	56.3 [32.0, 122.5]	-	-

Data are presented as median [Q1, Q3] or number (proportion). Abbreviations: BP = blood pressure, eGFR = estimated glomerular filtration rate, CAD = coronary artery disease, ECG = electrocardiogram, MI = myocardial infarction hs-cTn = high-sensitivity cardiac troponin.

Table S5: Detailed baseline characteristics for all cohorts of parameters used in the models

	ADAPT-BSN	ADPs-CH	BACC	BACC VTLi	FASTEST	High-STEACS	Lund	RAPID-CPU	ROMI	SAMIE	SEIGE, SAFETY	stenoCardia	STOP-CP	UTROPIA
**Sample size**	2315	3119	2575	125	1233	1750	1164	4938	1366	1985	1033	1688	1457	1631
**Sex (male)**	N:1386<br>%:59.9	N:1178<br>%:37.8	N:1638<br>%:63.6	N:73<br>%:60.8	N:677<br>%:55.0	N:1048<br>%:59.9	N:636<br>%:54.6	N:2867<br>%:58.1	N:650<br>%:47.7	N:1091<br>%:55.0	N:621<br>%:60.1	N:1108<br>%:65.6	N:781<br>%:53.6	N:911<br>%:55.9
**Age (years)**	Mean:52.6<br>Standard deviation:14.0<br>Skewness:0.4<br>Kurtosis:2.9<br>Q_1:43.0<br>Q_2:51.0<br>Q_3:61.0	Mean:62.6<br>Standard deviation:12.9<br>Skewness:0.0<br>Kurtosis:2.4<br>Q_1:53.0<br>Q_2:62.1<br>Q_3:72.4	Mean:62.7<br>Standard deviation:15.6<br>Skewness:-0.4<br>Kurtosis:2.5<br>Q_1:51.0<br>Q_2:64.0<br>Q_3:75.0	Mean:58.9<br>Standard deviation:17.8<br>Skewness:-0.2<br>Kurtosis:2.5<br>Q_1:49.8<br>Q_2:57.0<br>Q_3:72.2	Mean:63.1<br>Standard deviation:14.2<br>Skewness:-0.2<br>Kurtosis:2.7<br>Q_1:53.0<br>Q_2:64.0<br>Q_3:73.0	Mean:61.1<br>Standard deviation:14.0<br>Skewness:0.0<br>Kurtosis:2.5<br>Q_1:51.0<br>Q_2:60.0<br>Q_3:72.0	Mean:60.5<br>Standard deviation:17.5<br>Skewness:-0.4<br>Kurtosis:2.5<br>Q_1:49.0<br>Q_2:63.0<br>Q_3:74.0	Mean:62.6<br>Standard deviation:17.1<br>Skewness:-0.5<br>Kurtosis:2.5<br>Q_1:51.0<br>Q_2:64.0<br>Q_3:77.0	Mean:66.2<br>Standard deviation:16.8<br>Skewness:-0.5<br>Kurtosis:2.6<br>Q_1:55.0<br>Q_2:68.0<br>Q_3:80.0	Mean:56.2<br>Standard deviation:15.6<br>Skewness:-0.1<br>Kurtosis:2.5<br>Q_1:46.0<br>Q_2:56.0<br>Q_3:68.0	Mean:58.2<br>Standard deviation:15.9<br>Skewness:-0.1<br>Kurtosis:2.7<br>Q_1:48.0<br>Q_2:58.0<br>Q_3:69.0	Mean:61.4<br>Standard deviation:13.6<br>Skewness:-0.5<br>Kurtosis:2.8<br>Q_1:52.0<br>Q_2:63.0<br>Q_3:72.0	Mean:57.6<br>Standard deviation:12.8<br>Skewness:0.0<br>Kurtosis:2.9<br>Q_1:49.1<br>Q_2:57.5<br>Q_3:66.0	Mean:57.5<br>Standard deviation:15.3<br>Skewness:0.1<br>Kurtosis:2.9<br>Q_1:48.0<br>Q_2:57.0<br>Q_3:67.0
**Heart rate (bpm)**	Mean:77.3<br>Standard deviation:17.2<br>Skewness:1.4<br>Kurtosis:7.9<br>Q_1:65.0<br>Q_2:75.0<br>Q_3:86.0	Mean:72.7<br>Standard deviation:16.5<br>Skewness:1.4<br>Kurtosis:7.6<br>Q_1:62.0<br>Q_2:70.0<br>Q_3:81.0	Mean:80.2<br>Standard deviation:19.7<br>Skewness:1.4<br>Kurtosis:6.4<br>Q_1:67.0<br>Q_2:77.0<br>Q_3:88.5	Mean:79.1<br>Standard deviation:19.4<br>Skewness:1.6<br>Kurtosis:7.7<br>Q_1:64.0<br>Q_2:77.0<br>Q_3:90.0	Mean:73.1<br>Standard deviation:17.1<br>Skewness:1.5<br>Kurtosis:7.1<br>Q_1:61.0<br>Q_2:70.0<br>Q_3:82.0	Mean:77.0<br>Standard deviation:17.6<br>Skewness:1.2<br>Kurtosis:6.8<br>Q_1:64.0<br>Q_2:75.0<br>Q_3:87.0	Mean:81.5<br>Standard deviation:17.8<br>Skewness:1.1<br>Kurtosis:5.4<br>Q_1:70.0<br>Q_2:79.0<br>Q_3:90.0	Mean:78.9<br>Standard deviation:18.5<br>Skewness:1.4<br>Kurtosis:6.5<br>Q_1:67.0<br>Q_2:77.0<br>Q_3:87.0	-	Mean:77.9<br>Standard deviation:17.3<br>Skewness:1.3<br>Kurtosis:6.0<br>Q_1:66.0<br>Q_2:75.0<br>Q_3:86.5	Mean:89.9<br>Standard deviation:21.1<br>Skewness:0.7<br>Kurtosis:4.0<br>Q_1:75.0<br>Q_2:88.0<br>Q_3:102.0	Mean:73.0<br>Standard deviation:15.8<br>Skewness:1.1<br>Kurtosis:4.9<br>Q_1:62.0<br>Q_2:70.0<br>Q_3:81.0	Mean:78.7<br>Standard deviation:17.1<br>Skewness:0.9<br>Kurtosis:4.0<br>Q_1:66.0<br>Q_2:76.0<br>Q_3:89.0	Mean:89.3<br>Standard deviation:23.5<br>Skewness:0.4<br>Kurtosis:4.3<br>Q_1:74.0<br>Q_2:88.0<br>Q_3:103.0
**Hyperlipoproteinemia**	N:915<br>%:44.7	N:1767<br>%:56.7	N:904<br>%:35.1	N:29<br>%:23.2	N:378<br>%:30.8	N:687<br>%:41.0	N:262<br>%:22.5	N:2196<br>%:52.1	N:802<br>%:60.3	N:901<br>%:46.0	N:449<br>%:43.5	N:1236<br>%:73.2	N:698<br>%:47.9	N:696<br>%:43.1
**Ever smoker**	N:1413<br>%:61.0	N:460<br>%:14.8	N:1187<br>%:46.8	N:49<br>%:39.2	N:624<br>%:50.7	N:990<br>%:58.5	N:656<br>%:56.5	N:1010<br>%:24.2	N:821<br>%:63.2	N:444<br>%:22.4	N:617<br>%:59.7	N:865<br>%:52.6	-	N:969<br>%:60.1
**Family history of CAD**	N:945<br>%:40.8	N:1707<br>%:54.8	N:478<br>%:19.3	N:14<br>%:13.1	N:692<br>%:84.6	N:843<br>%:51.8	N:265<br>%:23.1	N:1236<br>%:31.0	N:714<br>%:58.9	N:843<br>%:46.3	N:213<br>%:20.6	N:540<br>%:33.2	-	-
**Ischaemic signs ECG**	N:20<br>%:0.9	N:321<br>%:10.3	N:520<br>%:20.8	N:18<br>%:14.9	N:252<br>%:20.9	N:193<br>%:11.3	N:69<br>%:6.0	N:1319<br>%:26.7	-	-	N:35<br>%:3.4	N:872<br>%:52.1	N:89<br>%:6.1	N:588<br>%:36.1
**Symptom onset ≥ 3h**	N:45<br>%:1.9	N:1698<br>%:72.5	N:1715<br>%:70.6	N:29<br>%:78.4	N:594<br>%:49.2	N:848<br>%:53.6	N:752<br>%:65.8	N:2802<br>%:56.7	N:385<br>%:54.2	N:604<br>%:47.8	N:747<br>%:72.4	N:1057<br>%:62.6	N:934<br>%:64.5	N:342<br>%:39.7
**log-transformed hs-cTnI Access - First measurement (ng/L)**	Mean:1.4<br>Standard deviation:1.1<br>Skewness:3.3<br>Kurtosis:16.3<br>Q_1:0.8<br>Q_2:0.9<br>Q_3:1.5	Mean:1.9<br>Standard deviation:1.7<br>Skewness:2.1<br>Kurtosis:7.4<br>Q_1:0.8<br>Q_2:1.2<br>Q_3:2.2	Mean:2.3<br>Standard deviation:1.7<br>Skewness:1.7<br>Kurtosis:5.4<br>Q_1:1.1<br>Q_2:1.7<br>Q_3:2.8	-	-	-	-	-	-	Mean:1.6<br>Standard deviation:1.1<br>Skewness:2.9<br>Kurtosis:14.7<br>Q_1:0.8<br>Q_2:1.1<br>Q_3:1.8	-	Mean:2.4<br>Standard deviation:2.1<br>Skewness:1.5<br>Kurtosis:4.7<br>Q_1:0.8<br>Q_2:1.6<br>Q_3:3.1	-	-
**log-transformed hs-cTnI Access - Second measurement (ng/L)**	Mean:1.5<br>Standard deviation:1.3<br>Skewness:3.2<br>Kurtosis:14.7<br>Q_1:0.8<br>Q_2:1.0<br>Q_3:1.6	Mean:2.0<br>Standard deviation:1.8<br>Skewness:2.2<br>Kurtosis:7.4<br>Q_1:0.8<br>Q_2:1.2<br>Q_3:2.2	Mean:2.4<br>Standard deviation:1.8<br>Skewness:1.6<br>Kurtosis:5.0<br>Q_1:1.1<br>Q_2:1.8<br>Q_3:3.0	-	-	-	-	-	-	Mean:1.8<br>Standard deviation:1.3<br>Skewness:2.7<br>Kurtosis:11.5<br>Q_1:0.8<br>Q_2:1.4<br>Q_3:2.1	-	Mean:2.8<br>Standard deviation:2.2<br>Skewness:1.4<br>Kurtosis:4.3<br>Q_1:1.2<br>Q_2:2.0<br>Q_3:3.7	-	-

**Fifth root of hs-cTnI Access rate ((ng/L)/hours)**	Mean:0.2<br>Standard deviation:0.8<br>Skewness:1.3<br>Kurtosis:8.1<br>Q_1:0<br>Q_2:0<br>Q_3:0.7	Mean:0.5<br>Standard deviation:1.1<br>Skewness:1.3<br>Kurtosis:5.9<br>Q_1:0<br>Q_2:0.5<br>Q_3:0.9	Mean:0.3<br>Standard deviation:1.4<br>Skewness:-0.1<br>Kurtosis:3.7<br>Q_1:-0.8<br>Q_2:0.6<br>Q_3:1.0	-	-	-	-	-	-	Mean:0.2<br>Standard deviation:0.8<br>Skewness:1.4<br>Kurtosis:8.3<br>Q_1:0<br>Q_2:0<br>Q_3:0.8	-	Mean:0.8<br>Standard deviation:1.3<br>Skewness:0.3<br>Kurtosis:5.9<br>Q_1:0<br>Q_2:0.9<br>Q_3:1.2	-	-
**log-transformed hs-cTnI Architect - First measurement (ng/L)**	Mean:1.3<br>Standard deviation:1.2<br>Skewness:3.1<br>Kurtosis:14.9<br>Q_1:0.6<br>Q_2:0.8<br>Q_3:1.5	Mean:2.1<br>Standard deviation:1.8<br>Skewness:1.9<br>Kurtosis:6.9<br>Q_1:0.8<br>Q_2:1.5<br>Q_3:2.6	Mean:2.2<br>Standard deviation:1.8<br>Skewness:1.8<br>Kurtosis:6.1<br>Q_1:1.0<br>Q_2:1.7<br>Q_3:2.8	-	Mean:1.8<br>Standard deviation:1.5<br>Skewness:2.1<br>Kurtosis:8.1<br>Q_1:0.7<br>Q_2:1.5<br>Q_3:2.2	Mean:1.8<br>Standard deviation:1.6<br>Skewness:2.1<br>Kurtosis:7.7<br>Q_1:0.6<br>Q_2:1.1<br>Q_3:2.2	-	-	Mean:2.1<br>Standard deviation:1.4<br>Skewness:1.5<br>Kurtosis:6.4<br>Q_1:0.7<br>Q_2:1.6<br>Q_3:2.8	-	Mean:2.4<br>Standard deviation:1.3<br>Skewness:1.7<br>Kurtosis:6.6<br>Q_1:1.4<br>Q_2:1.9<br>Q_3:3.0	Mean:2.7<br>Standard deviation:2.1<br>Skewness:1.6<br>Kurtosis:5.3<br>Q_1:1.3<br>Q_2:1.9<br>Q_3:3.4	-	Mean:2.0<br>Standard deviation:1.4<br>Skewness:1.4<br>Kurtosis:5.6<br>Q_1:0.6<br>Q_2:1.6<br>Q_3:2.8
**log-transformed hs-cTnI Architect - Second measurement (ng/L)**	Mean:1.4<br>Standard deviation:1.3<br>Skewness:3.1<br>Kurtosis:14.3<br>Q_1:0.6<br>Q_2:0.9<br>Q_3:1.6	Mean:2.2<br>Standard deviation:1.9<br>Skewness:1.8<br>Kurtosis:6.1<br>Q_1:0.8<br>Q_2:1.5<br>Q_3:2.7	Mean:2.3<br>Standard deviation:1.9<br>Skewness:1.6<br>Kurtosis:5.5<br>Q_1:1.0<br>Q_2:1.8<br>Q_3:3.0	-	Mean:2.0<br>Standard deviation:1.8<br>Skewness:2.2<br>Kurtosis:8.0<br>Q_1:0.7<br>Q_2:1.4<br>Q_3:2.3	Mean:2.0<br>Standard deviation:1.9<br>Skewness:2.0<br>Kurtosis:6.9<br>Q_1:0.6<br>Q_2:1.1<br>Q_3:2.5	-	-	Mean:2.2<br>Standard deviation:1.6<br>Skewness:1.7<br>Kurtosis:7.0<br>Q_1:1.1<br>Q_2:1.8<br>Q_3:3.0	-	Mean:2.5<br>Standard deviation:1.4<br>Skewness:1.7<br>Kurtosis:6.4<br>Q_1:1.4<br>Q_2:2.1<br>Q_3:3.2	Mean:2.9<br>Standard deviation:2.3<br>Skewness:1.4<br>Kurtosis:4.3<br>Q_1:1.3<br>Q_2:2.1<br>Q_3:3.6	-	Mean:2.3<br>Standard deviation:1.7<br>Skewness:1.6<br>Kurtosis:6.2<br>Q_1:0.9<br>Q_2:1.9<br>Q_3:3.0
**Fifth root of hs-cTnI Architect rate ((ng/L)/hours)**	Mean:0.2<br>Standard deviation:0.8<br>Skewness:1.3<br>Kurtosis:9.3<br>Q_1:0<br>Q_2:0<br>Q_3:0.7	Mean:0.4<br>Standard deviation:1.2<br>Skewness:0.7<br>Kurtosis:4.9<br>Q_1:-0.6<br>Q_2:0.6<br>Q_3:0.9	Mean:0.2<br>Standard deviation:1.3<br>Skewness:0.3<br>Kurtosis:3.7<br>Q_1:-0.9<br>Q_2:0<br>Q_3:1.1	-	Mean:0.3<br>Standard deviation:1.0<br>Skewness:1.1<br>Kurtosis:5.5<br>Q_1:0<br>Q_2:0<br>Q_3:0.9	Mean:0.3<br>Standard deviation:1.0<br>Skewness:1.1<br>Kurtosis:6.5<br>Q_1:0<br>Q_2:0<br>Q_3:0.9	-	-	Mean:0.3<br>Standard deviation:0.9<br>Skewness:1.1<br>Kurtosis:7.3<br>Q_1:0<br>Q_2:0<br>Q_3:0.9	-	Mean:0.2<br>Standard deviation:1.0<br>Skewness:0.2<br>Kurtosis:4.2<br>Q_1:0<br>Q_2:0<br>Q_3:0.9	Mean:0.6<br>Standard deviation:1.4<br>Skewness:0.7<br>Kurtosis:4.7<br>Q_1:-0.6<br>Q_2:0.7<br>Q_3:1.1	-	Mean:0.4<br>Standard deviation:1.0<br>Skewness:0.8<br>Kurtosis:8.0<br>Q_1:0<br>Q_2:0.7<br>Q_3:0.9
**log-transformed hs-cTnI Atellica - First measurement (ng/L)**	-	-	Mean:2.4<br>Standard deviation:1.8<br>Skewness:1.7<br>Kurtosis:5.7<br>Q_1:0.9<br>Q_2:1.7<br>Q_3:3.0	Mean:2.0<br>Standard deviation:1.5<br>Skewness:2.0<br>Kurtosis:7.1<br>Q_1:0.9<br>Q_2:1.4<br>Q_3:2.6	-	Mean:2.2<br>Standard deviation:1.7<br>Skewness:1.9<br>Kurtosis:6.8<br>Q_1:0.9<br>Q_2:1.5<br>Q_3:2.7	-	-	-	Mean:1.8<br>Standard deviation:1.2<br>Skewness:2.5<br>Kurtosis:11.5<br>Q_1:0.9<br>Q_2:1.3<br>Q_3:2.1	Mean:2.6<br>Standard deviation:1.5<br>Skewness:1.2<br>Kurtosis:4.8<br>Q_1:1.5<br>Q_2:2.4<br>Q_3:3.4	Mean:2.6<br>Standard deviation:2.0<br>Skewness:1.5<br>Kurtosis:4.8<br>Q_1:1.1<br>Q_2:1.9<br>Q_3:3.4	-	-
**log-transformed hs-cTnI Atellica - Second measurement (ng/L)**	-	-	Mean:2.5<br>Standard deviation:1.9<br>Skewness:1.6<br>Kurtosis:5.0<br>Q_1:1.0<br>Q_2:1.8<br>Q_3:3.2	-	-	Mean:2.4<br>Standard deviation:1.9<br>Skewness:1.8<br>Kurtosis:5.8<br>Q_1:0.9<br>Q_2:1.6<br>Q_3:2.9	-	-	-	Mean:2.0<br>Standard deviation:1.5<br>Skewness:2.3<br>Kurtosis:9.5<br>Q_1:0.9<br>Q_2:1.5<br>Q_3:2.4	Mean:2.7<br>Standard deviation:1.5<br>Skewness:1.2<br>Kurtosis:4.9<br>Q_1:1.6<br>Q_2:2.5<br>Q_3:3.5	Mean:2.9<br>Standard deviation:2.3<br>Skewness:1.4<br>Kurtosis:4.0<br>Q_1:1.2<br>Q_2:2.1<br>Q_3:3.7	-	-
**Fifth root of hs-cTnI Atellica rate ((ng/L)/hours)**	-	-	Mean:0.3<br>Standard deviation:1.3<br>Skewness:0.2<br>Kurtosis:5.8<br>Q_1:-0.7	-	-	Mean:0.4<br>Standard deviation:1.1<br>Skewness:0.3<br>Kurtosis:7.9<br>Q_1:-0.4	-	-	-	Mean:0.3<br>Standard deviation:0.9<br>Skewness:1.1<br>Kurtosis:7.7<br>Q_1:0	Mean:0.2<br>Standard deviation:1.1<br>Skewness:0.1<br>Kurtosis:3.2<br>Q_1:-0.8	Mean:0.7<br>Standard deviation:1.3<br>Skewness:0.8<br>Kurtosis:4.9<br>Q_1:0	-	-

			Q_2:0<br>Q_3:1.0			Q_2:0.4<br>Q_3:0.9				Q_2:0.5<br>Q_3:0.8	Q_2:0.6<br>Q_3:1.0	Q_2:0.8<br>Q_3:1.2		
**log-transformed hs-cTnI Atellica VTLi - First measurement (ng/L)**	-	-	-	Mean:2.0<br>Standard deviation:1.1<br>Skewness:1.5<br>Kurtosis:6.3<br>Q_1:1.3<br>Q_2:1.8<br>Q_3:2.4	-	-	-	-	-	-	Mean:2.2<br>Standard deviation:1.2<br>Skewness:1.1<br>Kurtosis:5.0<br>Q_1:1.4<br>Q_2:2.1<br>Q_3:2.8	-	-	-
**log-transformed hs-cTnI Atellica VTLi - Second measurement (ng/L)**	-	-	-	Mean:2.0<br>Standard deviation:1.1<br>Skewness:1.3<br>Kurtosis:4.9<br>Q_1:1.3<br>Q_2:1.8<br>Q_3:2.5	-	-	-	-	-	-	Mean:2.3<br>Standard deviation:1.3<br>Skewness:1.0<br>Kurtosis:4.6<br>Q_1:1.4<br>Q_2:2.1<br>Q_3:2.9	-	-	-
**Fifth root of hs-cTnI Atellica VTLi rate ((ng/L)/hours)**	-	-	-	Mean:0.0<br>Standard deviation:1.0<br>Skewness:0.2<br>Kurtosis:1.5<br>Q_1:-1.0<br>Q_2:0<br>Q_3:1.0	-	-	-	-	-	-	Mean:0.1<br>Standard deviation:1.0<br>Skewness:0.1<br>Kurtosis:1.8<br>Q_1:-0.9<br>Q_2:0.6<br>Q_3:1.0	-	-	-
**log-transformed hs-cTnT Elecsys - First measurement (ng/L)**	Mean:2.1<br>Standard deviation:0.9<br>Skewness:1.6<br>Kurtosis:6.2<br>Q_1:1.5<br>Q_2:1.9<br>Q_3:2.5	Mean:2.5<br>Standard deviation:1.1<br>Skewness:1.8<br>Kurtosis:6.4<br>Q_1:1.6<br>Q_2:2.1<br>Q_3:2.9	Mean:2.5<br>Standard deviation:1.2<br>Skewness:1.3<br>Kurtosis:4.7<br>Q_1:1.6<br>Q_2:2.2<br>Q_3:3.0	-	Mean:2.3<br>Standard deviation:0.9<br>Skewness:2.2<br>Kurtosis:9.8<br>Q_1:1.6<br>Q_2:1.9<br>Q_3:2.6	Mean:2.1<br>Standard deviation:1.1<br>Skewness:1.6<br>Kurtosis:6.3<br>Q_1:1.4<br>Q_2:1.8<br>Q_3:2.6	Mean:2.2<br>Standard deviation:1.0<br>Skewness:1.6<br>Kurtosis:6.6<br>Q_1:1.4<br>Q_2:1.9<br>Q_3:2.7	Mean:2.5<br>Standard deviation:1.1<br>Skewness:1.7<br>Kurtosis:6.1<br>Q_1:1.6<br>Q_2:2.2<br>Q_3:3.0	Mean:2.6<br>Standard deviation:1.1<br>Skewness:0.8<br>Kurtosis:3.6<br>Q_1:1.8<br>Q_2:2.5<br>Q_3:3.3	-	-	Mean:2.5<br>Standard deviation:1.3<br>Skewness:1.5<br>Kurtosis:5.1<br>Q_1:1.6<br>Q_2:2.2<br>Q_3:3.0	Mean:2.5<br>Standard deviation:1.1<br>Skewness:1.2<br>Kurtosis:4.7<br>Q_1:1.6<br>Q_2:2.2<br>Q_3:3.0	-
**log-transformed hs-cTnT Elecsys - Second measurement (ng/L)**	Mean:2.1<br>Standard deviation:1.0<br>Skewness:1.8<br>Kurtosis:7.1<br>Q_1:1.4<br>Q_2:1.9<br>Q_3:2.4	Mean:2.5<br>Standard deviation:1.2<br>Skewness:1.8<br>Kurtosis:6.2<br>Q_1:1.6<br>Q_2:2.0<br>Q_3:2.9	Mean:2.5<br>Standard deviation:1.3<br>Skewness:1.2<br>Kurtosis:4.4<br>Q_1:1.6<br>Q_2:2.2<br>Q_3:3.1	-	Mean:2.3<br>Standard deviation:1.0<br>Skewness:2.4<br>Kurtosis:10.7<br>Q_1:1.6<br>Q_2:1.9<br>Q_3:2.7	Mean:2.1<br>Standard deviation:1.8<br>Skewness:2.2<br>Kurtosis:7.8<br>Q_1:1.1<br>Q_2:1.1<br>Q_3:2.5	Mean:2.3<br>Standard deviation:1.0<br>Skewness:1.7<br>Kurtosis:6.9<br>Q_1:1.4<br>Q_2:1.9<br>Q_3:2.8	Mean:2.7<br>Standard deviation:1.2<br>Skewness:1.5<br>Kurtosis:5.5<br>Q_1:1.8<br>Q_2:2.3<br>Q_3:3.2	Mean:2.6<br>Standard deviation:1.1<br>Skewness:1.0<br>Kurtosis:4.2<br>Q_1:1.8<br>Q_2:2.5<br>Q_3:3.3	-	-	Mean:2.6<br>Standard deviation:1.5<br>Skewness:1.4<br>Kurtosis:4.7<br>Q_1:1.4<br>Q_2:2.1<br>Q_3:3.1	Mean:2.5<br>Standard deviation:1.1<br>Skewness:1.2<br>Kurtosis:4.8<br>Q_1:1.6<br>Q_2:2.2<br>Q_3:3.1	-
**Fifth root of hs-cTnT Elecsys rate ((ng/L)/hours)**	Mean:0.0<br>Standard deviation:0.8<br>Skewness:0.6<br>Kurtosis:2.9<br>Q_1:-0.7<br>Q_2:-0.3<br>Q_3:0.7	Mean:0.2<br>Standard deviation:1.0<br>Skewness:0.3<br>Kurtosis:2.5<br>Q_1:-0.8<br>Q_2:0<br>Q_3:0.9	Mean:0.1<br>Standard deviation:1.1<br>Skewness:0.2<br>Kurtosis:2.2<br>Q_1:-1.0<br>Q_2:0<br>Q_3:1.0	-	Mean:0.0<br>Standard deviation:0.8<br>Skewness:0.8<br>Kurtosis:5.4<br>Q_1:0<br>Q_2:0<br>Q_3:0	Mean:-0.1<br>Standard deviation:1.5<br>Skewness:1.9<br>Kurtosis:10.6<br>Q_1:-1.1<br>Q_2:-0.8<br>Q_3:0.8	Mean:0.0<br>Standard deviation:0.8<br>Skewness:0.5<br>Kurtosis:3.5<br>Q_1:-0.9<br>Q_2:0<br>Q_3:0	Mean:0.1<br>Standard deviation:1.1<br>Skewness:0.2<br>Kurtosis:2.4<br>Q_1:-1.0<br>Q_2:0<br>Q_3:1.0	Mean:0.0<br>Standard deviation:0.9<br>Skewness:0.5<br>Kurtosis:3.0<br>Q_1:-0.8<br>Q_2:0<br>Q_3:0.8	-	-	Mean:-0.1<br>Standard deviation:1.1<br>Skewness:0.9<br>Kurtosis:3.2<br>Q_1:-0.9<br>Q_2:-0.7<br>Q_3:0.8	Mean:0.0<br>Standard deviation:1.0<br>Skewness:0.2<br>Kurtosis:1.7<br>Q_1:-1.0<br>Q_2:-0.5<br>Q_3:1.0	-
**log-transformed hs-cTnI Pathfast - First measurement (ng/L)**	-	-	Mean:2.1<br>Standard deviation:1.8<br>Skewness:2.0	-	-	-	-	-	-	-	-	Mean:2.2<br>Standard deviation:1.9<br>Skewness:2.0	-	-

			Kurtosis:6.5<br>Q_1:0.8<br>Q_2:1.3<br>Q_3:2.5									Kurtosis:7.0<br>Q_1:0.8<br>Q_2:1.4<br>Q_3:2.5		
**log-transformed hs-cTnI Pathfast - Second measurement (ng/L)**	-	-	Mean:2.2<br>Standard deviation:1.9<br>Skewness:1.8<br>Kurtosis:5.8<br>Q_1:0.8<br>Q_2:1.4<br>Q_3:2.7	-	-	-	-	-	-	-	-	Mean:2.6<br>Standard deviation:2.2<br>Skewness:1.7<br>Kurtosis:5.0<br>Q_1:0.9<br>Q_2:1.6<br>Q_3:3.0	-	-
**Fifth root of hs-cTnI Pathfast rate ((ng/L)/hours)**	-	-	Mean:0.2<br>Standard deviation:1.3<br>Skewness:0.2<br>Kurtosis:4.5<br>Q_1:-0.8<br>Q_2:0<br>Q_3:1.0	-	-	-	-	-	-	-	-	Mean:0.5<br>Standard deviation:1.4<br>Skewness:0.3<br>Kurtosis:6.1<br>Q_1:-0.6<br>Q_2:0.7<br>Q_3:1.1	-	-

For continuous variables mean, standard deviation, skewness, kurtosis and quartiles (Q_1, Q_2, Q_3) are presented. For binary variables absolute (N) and relative frequencies (%). Continuous variables are presented in the form that is expected by the ARTEMIS model (i.e., in some cases after a transformation). The first and second troponin measurements were log-transformed (the natural logarithm was used). The troponin rate is defined as the difference of the second minus the first measurement divided by the time between measurements. The troponin rate was used after taking its fifth root. Abbreviations: CAD = coronary artery disease, ECG = electrocardiogram, hs-cTn = high-sensitivity cardiac troponin.

Table S6: Variables and machines selected for the single and the serial hs-cTn measurement models

Single hs-cTn measurement model		Serial hs-cTn measurement model	
**Variables included**	**Machines included**	**Variables included**	**Machines included**
Hs-cTn at baseline (0h)	Logistic regression with restricted cubic splines	Hs-cTn at baseline (0h)	Logistic regression with restricted cubic splines
Age	Gradient boosting machine	Serial hs-cTn measurement	Gradient boosting machine
Sex	Multivariate adaptive regression spline	Time between serial sampling (minutes)	Multivariate adaptive regression spline
Heart rate	Elastic net	Age	Random forest
Ever smoker		Sex	
Hyperlipoproteinemia		Heart rate	
Family history of CAD		Hyperlipoproteinemia	
Ischemic signs in ECG		Symptom onset ≥ 3 hours	
Symptom onset ≥ 3 hours			

Table S7: Discrimination measures (logLoss, AUC, BS) for single and serial hs-cTn measurement for each cohort and the global dataset per assay, uncalibrated and calibrated using logistic regression, calibration using logistic regression with restricted cubic splines and by Elkan calibration.

- Attached as Excel-file -

Table S8: Diagnostic performance measures for using different MI probabilities as thresholds for the single hs-cTn measurement model

- Attached as Excel-file -

This table displays the diagnostic performance measures using the MI probability as threshold. These analyses are based on data from the validation cohort stenoCardia as well as the generalization cohorts ADAPT-BSN, ADPs-CH, DROP-ACS, FASTEST, LUND, RAPID-CPU, ROMI, SAMI, SEIGE & SAFETY, STOP-CP and UTROPIA. Only for the model performance using the Elecsys and Architect assay, DROP-ACS and ROMI cohorts were excluded, as they did not represent the entire spectrum of MI probability.

Table S9: Diagnostic performance measures for using different MI probabilities as thresholds for the serial hs-cTn measurement model

- Attached as Excel-file -

This table displays the diagnostic performance measures using the MI probability as threshold. These analyses are based on data from the validation cohort stenoCardia as well as the generalization cohorts ADAPT-BSN, ADPs-CH, DROP-ACS, FASTEST, LUND, RAPID-CPU, ROMI, SAMI, SEIGE & SAFETY, STOP-CP and UTROPIA. Only for the model performance using the Elecsys and Architect assay, DROP-ACS and ROMI cohorts were excluded, as they did not represent the entire spectrum of MI probability.

Table S10: Comparison of ESC 0h, 0/1h and 0/2h algorithms with the ARTEMIS pathway using a single or a serial hs-cTn measurement.

		ESC 0h Algorithm					
		Elecsys	Architect	Atellica	Access	Pathfast	AtellicaVTLi
		Mean [95% CI]	Mean [95% CI]	Mean [95% CI]	Mean [95% CI]	Mean [95% CI]	Mean [95% CI]
Rule-In	NPV	93.70 [91.70, 95.25]	94.81 [93.19, 96.07]	95.42 [93.22, 96.92]	95.95 [94.05, 97.26]	96.18 [94.99, 97.38]	92.85 [91.23, 94.47]
	PPV	76.59 [65.62, 84.87]	75.83 [58.02, 87.69]	66.60 [45.64, 82.56]	80.88 [72.37, 87.23]	71.07 [68.24, 73.90]	60.00 [56.92, 63.08]
	Sensitivity	53.31 [46.41, 60.09]	58.95 [43.66, 72.69]	53.33 [32.34, 73.20]	61.89 [43.14, 77.67]	72.27 [69.47, 75.06]	23.86 [21.18, 26.54]
	Specificity	97.70 [96.32, 98.57]	97.54 [94.50, 98.91]	97.28 [94.13, 98.76]	98.44 [94.39, 99.58]	95.96 [94.73, 97.19]	98.42 [97.63, 99.20]
Rule-Out	NPV	99.16 [93.94, 99.89]	99.33 [98.92, 99.59]	99.87 [98.83, 99.99]	99.55 [98.43, 99.87]	100.00 [100.00, 100.00]	100.00 [100.00, 100.00]
	PPV	14.71 [10.94, 19.51]	14.67 [9.22, 22.54]	11.08 [6.04, 19.47]	13.51 [6.99, 24.49]	14.27 [12.09, 16.45]	10.59 [8.66, 12.52]
	Sensitivity	99.23 [98.65, 99.57]	98.77 [98.25, 99.14]	99.50 [96.65, 99.93]	98.84 [97.48, 99.47]	100.00 [100.00, 100.00]	100.00 [100.00, 100.00]
	Specificity	15.66 [11.65, 20.73]	16.42 [8.65, 28.95]	15.23 [10.01, 22.50]	16.44 [6.59, 35.44]	17.53 [15.16, 19.91]	15.95 [13.65, 18.25]
		n (% [95% CI])	n (% [95% CI])	n (% [95% CI])	n (% [95% CI])	n (% [95% CI])	n (% [95% CI])
Proportions	Rule-out	1457 (13.74% [10.35, 18.02])	1009 (14.16% [7.71, 24.58])	616 (13.67% [8.36, 21.55])	724 (14.29% [5.94, 30.56])	152 (15.42% [13.16, 17.67])	141 (14.51% [12.29, 16.72])
	Observe	8138 (76.74% [70.33, 82.12])	5264 (73.87% [58.03, 85.26])	3466 (76.96% [73.51, 80.09])	3750 (74.00% [50.77, 88.70])	713 (72.31% [69.52, 75.11])	796 (81.89% [79.47, 84.31])
	Rule-in	933 (8.80% [6.87, 11.21])	665 (9.33% [5.59, 15.17])	345 (7.65% [3.24, 17.01])	401 (7.91% [3.33, 17.61])	121 (12.27% [10.22, 14.32])	35 (3.60% [2.43, 4.77])

Rule-out threshold: 0.5% MI Rule-in threshold: 50% MI		ARTEMIS single (0h) hs-cTn measurement model, rule-out: 0.5% MI probability, rule-in: 50% MI probability					
		Elecsys	Architect	Atellica	Access	Pathfast	AtellicaVTLi
		Mean [95% CI]	Mean [95% CI]	Mean [95% CI]	Mean [95% CI]	Mean [95% CI]	Mean [95% CI]
Rule-In	NPV	93.86 [92.14, 95.22]	94.51 [93.10, 95.64]	95.13 [91.74, 97.17]	96.00 [94.73, 96.96]	95.75 [94.59, 96.90]	92.56 [90.91, 94.21]
	PPV	73.85 [66.91, 79.77]	74.54 [67.34, 80.61]	70.55 [52.99, 83.58]	78.44 [68.79, 85.72]	76.51 [74.08, 78.93]	58.06 [54.96, 61.17]
	Sensitivity	54.29 [42.08, 66.00]	52.24 [32.57, 71.24]	45.29 [14.40, 80.30]	62.83 [48.28, 75.38]	74.71 [72.22, 77.19]	20.45 [17.92, 22.99]
	Specificity	97.15 [96.18, 97.88]	97.76 [96.38, 98.62]	98.24 [95.79, 99.27]	98.07 [96.68, 98.89]	96.13 [95.02, 97.23]	98.53 [97.77, 99.29]
Rule-Out	NPV	99.61 [99.23, 99.80]	99.65 [99.45, 99.78]	99.83 [99.59, 99.93]	99.69 [99.39, 99.84]	99.68 [99.36, 100.00]	99.69 [99.34, 100.00]
	PPV	16.66 [12.44, 21.94]	17.79 [12.35, 24.93]	18.92 [8.74, 36.25]	18.64 [14.27, 23.98]	19.63 [17.36, 21.90]	13.41 [11.27, 15.55]
	Sensitivity	99.05 [97.86, 99.58]	99.26 [98.17, 99.70]	99.09 [95.75, 99.81]	98.76 [98.31, 99.09]	99.41 [98.97, 99.85]	98.86 [98.20, 99.53]
	Specificity	34.91 [23.22, 48.75]	34.97 [17.91, 56.99]	54.70 [42.42, 66.42]	49.41 [37.21, 61.68]	31.28 [28.63, 33.93]	36.45 [33.41, 39.48]
		n (% [95% CI])	n (% [95% CI])	n (% [95% CI])	n (% [95% CI])	n (% [95% CI])	n (% [95% CI])

		Elecsys	Architect	Atellica	Access	Pathfast	AtellicaVTLi
Proportions	Rule-out	3266 (30.80% [21.06, 42.62])	2171 (30.47% [15.73, 50.70])	2193 (48.70% [40.71, 56.76])	2239 (44.18% [31.58, 57.59])	265 (26.85% [24.32, 29.38])	323 (33.25% [30.28, 36.22])
	Observe	7052 (66.50% [49.29, 80.22])	4207 (59.04% [41.06, 74.89])	1967 (43.67% [27.98, 60.74])	2330 (45.99% [37.74, 54.47])	582 (59.05% [56.24, 61.86])	618 (63.56% [60.53, 66.59])
	Rule-in	954 (9.00% [6.27, 12.77])	549 (7.70% [4.34, 13.31])	249 (5.52% [1.57, 17.63])	411 (8.11% [4.21, 15.06])	139 (14.10% [12.12, 16.09])	31 (3.19% [2.08, 4.29])

ESC 0/1h Algorithm

		Elecsys	Architect	Atellica	Access	Pathfast	AtellicaVTLi
		Mean [95% CI]	Mean [95% CI]	Mean [95% CI]	Mean [95% CI]	Mean [95% CI]	Mean [95% CI]
Rule-In	NPV	97.62 [96.08, 98.56]	97.80 [96.26, 98.72]				
	PPV	65.78 [60.30, 70.87]	73.30 [47.14, 89.42]				
	Sensitivity	81.49 [76.55, 85.59]	82.92 [67.74, 91.82]				
	Specificity	94.75 [92.32, 96.45]	96.64 [94.84, 97.83]				
Rule-Out	NPV	99.52 [98.39, 99.86]	99.68 [98.59, 99.93]				
	PPV	29.99 [20.93, 40.94]	28.42 [15.81, 45.63]				
	Sensitivity	97.55 [93.48, 99.10]	98.77 [97.39, 99.42]				
	Specificity	71.63 [63.69, 78.42]	67.86 [63.99, 71.50]				
		n (% [95% CI])	n (% [95% CI])	n (% [95% CI])	n (% [95% CI])	n (% [95% CI])	n (% [95% CI])
Proportions	Rule-out	2239 (63.67% [56.11, 70.60])	345 (58.27% [54.26, 62.19])				
	Observe	788 (22.41% [17.83, 27.76])	156 (26.36% [22.97, 30.06])				
	Rule-in	479 (13.63% [9.74, 18.75])	87 (14.75% [10.41, 20.48])				

ARTEMIS serial (0/1h) hs-cTn measurement model, rule-out: 0.5% MI probability, rule-in: 50% MI probability

Rule-out threshold: 0.5% MI Rule-in threshold: 50% MI		Elecsys	Architect	Atellica	Access	Pathfast	AtellicaVTLi
		Mean [95% CI]	Mean [95% CI]	Mean [95% CI]	Mean [95% CI]	Mean [95% CI]	Mean [95% CI]
Rule-In	NPV	95.90 [94.72, 96.83]	96.27 [94.41, 97.53]				
	PPV	75.35 [68.86, 80.85]	68.60 [33.02, 90.64]				
	Sensitivity	66.72 [58.70, 73.88]	66.56 [35.21, 87.94]				
	Specificity	97.28 [95.66, 98.31]	96.84 [95.07, 97.98]				

		Elecsys	Architect	Atellica	Access	Pathfast	AtellicaVTLi
Rule-Out	NPV	99.36 [94.70, 99.93]	99.59 [98.48, 99.89]				
	PPV	19.16 [15.74, 23.13]	23.77 [16.08, 33.68]				
	Sensitivity	98.71 [98.28, 99.04]	98.77 [97.39, 99.42]				
	Specificity	34.09 [15.03, 60.20]	52.95 [48.91, 56.96]				
		n (% [95% CI])	n (% [95% CI])	n (% [95% CI])	n (% [95% CI])	n (% [95% CI])	n (% [95% CI])
Proportions	Rule-out	1054 (29.98% [13.58, 53.83])	281 (47.47% [38.78, 56.31])				
	Observe	1872 (53.25% [30.17, 75.01])	240 (40.57% [36.67, 44.59])				
	Rule-in	344 (9.78% [6.42, 14.61])	75 (12.62% [7.63, 20.17])				

Rule-out threshold: 1% MI Rule-in threshold: 50% MI		ARTEMIS serial (0/1h) hs-cTn measurement model, rule-out: 1% MI probability, rule-in: 50% MI probability					
		Elecsys	Architect	Atellica	Access	Pathfast	AtellicaVTLi
		Mean [95% CI]	Mean [95% CI]	Mean [95% CI]	Mean [95% CI]	Mean [95% CI]	Mean [95% CI]
Rule-In	NPV	95.90 [94.72, 96.83]	96.27 [94.41, 97.53]				
	PPV	75.35 [68.86, 80.85]	68.60 [33.02, 90.64]				
	Sensitivity	66.72 [58.70, 73.88]	66.56 [35.21, 87.94]				
	Specificity	97.28 [95.66, 98.31]	96.84 [95.07, 97.98]				
Rule-Out	NPV	99.57 [99.18, 99.78]	99.06 [93.07, 99.88]				
	PPV	23.98 [19.94, 28.55]	27.31 [17.70, 39.63]				
	Sensitivity	98.36 [97.53, 98.91]	93.90 [38.12, 99.74]				
	Specificity	58.05 [37.28, 76.32]	66.85 [54.01, 77.60]				
		n (% [95% CI])	n (% [95% CI])	n (% [95% CI])	n (% [95% CI])	n (% [95% CI])	n (% [95% CI])
Proportions	Rule-out	1808 (51.41% [32.59, 69.84])	361 (60.90% [42.79, 76.44])				
	Observe	1314 (37.37% [22.78, 54.68])	169 (28.60% [19.23, 40.25])				
	Rule-in	344 (9.78% [6.42, 14.61])	75 (12.62% [7.63, 20.17])				

Rule-out threshold: 2% MI		ARTEMIS serial (0/1h) hs-cTn measurement model, rule-out: 2% MI probability, rule-in: 50% MI probability					
		Elecsys	Architect	Atellica	Access	Pathfast	AtellicaVTLi

Rule-in threshold: 50% MI		Mean [95% CI]	Mean [95% CI]	Mean [95% CI]	Mean [95% CI]	Mean [95% CI]	Mean [95% CI]
Rule-In	NPV	95.90 [94.72, 96.83]	96.27 [94.41, 97.53]				
	PPV	75.35 [68.86, 80.85]	68.60 [33.02, 90.64]				
	Sensitivity	66.72 [58.70, 73.88]	66.56 [35.21, 87.94]				
	Specificity	97.28 [95.66, 98.31]	96.84 [95.07, 97.98]				
Rule-Out	NPV	99.22 [98.68, 99.54]	98.81 [84.92, 99.92]				
	PPV	29.74 [26.30, 33.44]	32.19 [18.89, 49.19]				
	Sensitivity	95.40 [92.61, 97.17]	90.61 [13.12, 99.84]				
	Specificity	71.89 [63.80, 78.78]	77.76 [63.10, 87.73]				
		n (% [95% CI])	n (% [95% CI])	n (% [95% CI])	n (% [95% CI])	n (% [95% CI])	n (% [95% CI])
Proportions	Rule-out	2264 (64.41% [54.58, 73.15])	425 (71.78% [48.42, 87.33])				
	Observe	894 (25.44% [20.48, 31.14])	103 (17.34% [7.46, 35.33])				
	Rule-in	344 (9.78% [6.42, 14.61])	75 (12.62% [7.63, 20.17])				

ESC 0/2h Algorithm

		Elecsys	Architect	Atellica	Access	Pathfast	AtellicaVTLi
		Mean [95% CI]	Mean [95% CI]	Mean [95% CI]	Mean [95% CI]	Mean [95% CI]	Mean [95% CI]
Rule-In	NPV	96.71 [94.52, 98.05]	97.34 [95.99, 98.24]	97.95 [92.16, 99.49]	97.33 [96.19, 98.14]		
	PPV	80.05 [74.02, 84.97]	76.43 [60.15, 87.44]	64.24 [50.04, 76.32]	79.64 [64.31, 89.46]		
	Sensitivity	78.42 [70.83, 84.47]	80.51 [73.64, 85.94]	70.06 [59.87, 78.58]	77.56 [64.19, 86.95]		
	Specificity	97.01 [95.33, 98.10]	96.64 [93.22, 98.37]	95.61 [91.19, 97.87]	98.01 [88.60, 99.68]		
Rule-Out	NPV	99.18 [98.81, 99.43]	99.73 [98.54, 99.95]	99.46 [97.07, 99.90]	99.78 [98.95, 99.95]		
	PPV	37.84 [29.13, 47.42]	29.49 [20.47, 40.46]	19.54 [10.31, 33.92]	24.34 [15.66, 35.79]		
	Sensitivity	96.31 [93.86, 97.80]	98.50 [97.71, 99.02]	87.50 [82.72, 91.10]	98.58 [97.49, 99.20]		
	Specificity	76.30 [69.15, 82.21]	66.60 [46.16, 82.26]	64.56 [39.03, 83.83]	69.61 [55.01, 81.10]		
		n (% [95% CI])	n (% [95% CI])	n (% [95% CI])	n (% [95% CI])	n (% [95% CI])	n (% [95% CI])
Proportions	Rule-out	2820 (66.53% [59.07, 73.24])	2557 (57.89% [39.16, 74.60])	736 (58.74% [34.10, 79.66])	1250 (62.86% [48.76, 75.07])		

		Elecsys	Architect	Atellica	Access	Pathfast	AtellicaVTLi
	Observe	873 (20.62% [16.80, 25.04])	1232 (27.90% [15.86, 44.28])	361 (28.78% [12.86, 52.54])	505 (25.39% [17.27, 35.69])		
	Rule-in	531 (12.53% [9.19, 16.86])	557 (12.66% [8.13, 19.19])	130 (10.35% [4.93, 20.44])	186 (9.34% [4.07, 20.02])		

ARTEMIS serial (0/2h) hs-cTn measurement model, rule-out: 0.5% MI probability, rule-in: 50% MI probability

Rule-out threshold: 0.5% MI Rule-in threshold: 50% MI		Elecsys	Architect	Atellica	Access	Pathfast	AtellicaVTLi
		Mean [95% CI]	Mean [95% CI]	Mean [95% CI]	Mean [95% CI]	Mean [95% CI]	Mean [95% CI]
Rule-In	NPV	95.84 [94.40, 96.92]	96.63 [94.49, 97.96]	97.12 [91.93, 99.01]	97.45 [96.41, 98.19]		
	PPV	80.61 [77.00, 83.78]	75.32 [66.40, 82.49]	76.74 [63.77, 86.09]	78.95 [71.25, 85.02]		
	Sensitivity	72.07 [65.86, 77.53]	72.86 [47.12, 89.00]	61.91 [34.59, 83.33]	77.43 [59.84, 88.77]		
	Specificity	97.32 [96.20, 98.12]	97.72 [96.10, 98.68]	98.45 [97.55, 99.03]	98.20 [97.27, 98.82]		
Rule-Out	NPV	98.37 [94.60, 99.52]	99.48 [96.75, 99.92]	99.00 [95.19, 99.80]	98.75 [68.50, 99.97]		
	PPV	20.00 [16.97, 23.41]	24.15 [14.94, 36.57]	18.33 [5.82, 44.88]	20.88 [9.86, 38.91]		
	Sensitivity	98.87 [96.71, 99.62]	98.27 [96.88, 99.05]	95.11 [89.85, 97.71]	96.65 [90.61, 98.85]		
	Specificity	24.31 [9.07, 50.85]	59.90 [47.84, 70.87]	59.89 [32.81, 82.04]	56.71 [35.13, 76.01]		
Proportions		n (% [95% CI])	n (% [95% CI])	n (% [95% CI])	n (% [95% CI])	n (% [95% CI])	n (% [95% CI])
	Rule-out	890 (20.99% [8.11, 44.42])	2367 (53.58% [42.52, 64.31])	682 (54.42% [35.91, 71.78])	1034 (52.00% [35.30, 68.26])		
	Observe	2450 (57.80% [35.14, 77.60])	1552 (35.13% [24.45, 47.53])	489 (39.01% [14.22, 71.15])	715 (35.95% [18.71, 57.78])		
	Rule-in	486 (11.46% [8.14, 15.90])	420 (9.50% [4.52, 18.90])	71 (5.70% [1.90, 15.90])	164 (8.24% [3.90, 16.58])		

ARTEMIS serial (0/2h) hs-cTn measurement model, rule-out: 1% MI probability, rule-in: 50% MI probability

Rule-out threshold: 1% MI Rule-in threshold: 50% MI		Elecsys	Architect	Atellica	Access	Pathfast	AtellicaVTLi
		Mean [95% CI]	Mean [95% CI]	Mean [95% CI]	Mean [95% CI]	Mean [95% CI]	Mean [95% CI]
Rule-In	NPV	95.84 [94.40, 96.92]	96.63 [94.49, 97.96]	97.12 [91.93, 99.01]	97.45 [96.41, 98.19]		
	PPV	80.61 [77.00, 83.78]	75.32 [66.40, 82.49]	76.74 [63.77, 86.09]	78.95 [71.25, 85.02]		
	Sensitivity	72.07 [65.86, 77.53]	72.86 [47.12, 89.00]	61.91 [34.59, 83.33]	77.43 [59.84, 88.77]		
	Specificity	97.32 [96.20, 98.12]	97.72 [96.10, 98.68]	98.45 [97.55, 99.03]	98.20 [97.27, 98.82]		
Rule-Out	NPV	99.51 [98.75, 99.81]	99.48 [96.75, 99.92]	99.60 [98.59, 99.89]	99.32 [94.58, 99.92]		
	PPV	27.01 [24.48, 29.70]	24.15 [14.94, 36.57]	23.59 [8.11, 51.93]	26.95 [15.10, 43.35]		

		Elecsys	Architect	Atellica	Access	Pathfast	AtellicaVTLi
	Sensitivity	98.22 [97.09, 98.92]	98.27 [96.88, 99.05]	97.63 [91.94, 99.34]	96.60 [92.17, 98.56]		
	Specificity	60.79 [44.68, 74.85]	59.90 [47.84, 70.87]	71.18 [56.43, 82.49]	72.78 [56.84, 84.44]		
		n (% [95% CI])	n (% [95% CI])	n (% [95% CI])	n (% [95% CI])	n (% [95% CI])	n (% [95% CI])
Proportions	Rule-out	2243) (52.91% [37.08, 68.17])	2367 (53.58% [42.52, 64.31])	792 (63.24% [52.98, 72.42])	1313 (66.05% [52.68, 77.27])		
	Observe	1464 (34.53% [24.08, 46.73])	1552 (35.13% [24.45, 47.53])	338 (26.97% [14.74, 44.10])	467 (23.49% [14.24, 36.23])		
	Rule-in	486 (11.46% [8.14, 15.90])	420 (9.50% [4.52, 18.90])	71 (5.70% [1.90, 15.90])	164 (8.24% [3.90, 16.58])		

Rule-out threshold: 2% MI Rule-in threshold: 50% MI		ARTEMIS serial (0/2h) hs-cTn measurement model, rule-out: 2% MI probability, rule-in: 50% MI probability					
		Elecsys	Architect	Atellica	Access	Pathfast	AtellicaVTLi
		Mean [95% CI]	Mean [95% CI]	Mean [95% CI]	Mean [95% CI]	Mean [95% CI]	Mean [95% CI]
Rule-In	NPV	95.84 [94.40, 96.92]	96.63 [94.49, 97.96]	97.12 [91.93, 99.01]	97.45 [96.41, 98.19]		
	PPV	80.61 [77.00, 83.78]	75.32 [66.40, 82.49]	76.74 [63.77, 86.09]	78.95 [71.25, 85.02]		
	Sensitivity	72.07 [65.86, 77.53]	72.86 [47.12, 89.00]	61.91 [34.59, 83.33]	77.43 [59.84, 88.77]		
	Specificity	97.32 [96.20, 98.12]	97.72 [96.10, 98.68]	98.45 [97.55, 99.03]	98.20 [97.27, 98.82]		
Rule-Out	NPV	99.34 [98.90, 99.61]	99.46 [98.92, 99.74]	99.53 [98.68, 99.83]	99.52 [98.62, 99.83]		
	PPV	36.27 [31.66, 41.16]	37.44 [26.08, 50.38]	30.37 [11.65, 59.05]	38.90 [24.27, 55.86]		
	Sensitivity	97.21 [96.34, 97.87]	96.67 [94.74, 97.91]	95.05 [76.03, 99.15]	95.18 [87.50, 98.23]		
	Specificity	74.87 [65.91, 82.12]	81.72 [74.29, 87.37]	81.23 [64.71, 91.08]	86.09 [82.83, 88.80]		
		n (% [95% CI])	n (% [95% CI])	n (% [95% CI])	n (% [95% CI])	n (% [95% CI])	n (% [95% CI])
Proportions	Rule-out	2768 (65.28% [54.74, 74.51])	3230 (73.13% [64.12, 80.57])	919 (73.38% [58.37, 84.42])	1565 (78.72% [73.56, 83.10])		
	Observe	963 (22.71% [16.83, 29.91])	695 (15.74% [9.79, 24.34])	218 (17.40% [7.34, 35.90])	242 (12.19% [8.77, 16.69])		
	Rule-in	486 (11.46% [8.14, 15.90])	420 (9.50% [4.52, 18.90])	71 (5.70% [1.90, 15.90])	164 (8.24% [3.90, 16.58])		

This table compares the diagnostic performance of the ESC 0h, 0/1h and 0/2h algorithm to the ARTEMIS pathway using either a single hs-cTn (0h) or a serial hs-cTn (based on 0/1h or 0/2h samples) measurement. For the ARTEMIS pathway the performance is displayed using either an MI probability threshold of <0.5%, <1% or <2%, while the rule-in threshold was always set to >50%. Due to the meta-analytic background of the analyses, the proportions of rule-out, observe and rule-in zone due not sum up to 100%.
